# Hamster PIWI proteins bind to piRNAs with stage-specific size variations during oocyte maturation

**DOI:** 10.1093/nar/gkab059

**Published:** 2021-02-15

**Authors:** Kyoko Ishino, Hidetoshi Hasuwa, Jun Yoshimura, Yuka W Iwasaki, Hidenori Nishihara, Naomi M Seki, Takamasa Hirano, Marie Tsuchiya, Hinako Ishizaki, Harumi Masuda, Tae Kuramoto, Kuniaki Saito, Yasubumi Sakakibara, Atsushi Toyoda, Takehiko Itoh, Mikiko C Siomi, Shinichi Morishita, Haruhiko Siomi

**Affiliations:** Department of Molecular Biology, Keio University School of Medicine, Tokyo 160-8582, Japan; Department of Molecular Biology, Keio University School of Medicine, Tokyo 160-8582, Japan; Department of Computational Biology and Medical Sciences, Graduate School of Frontier Sciences, The University of Tokyo, Tokyo 113-0032, Japan; Department of Molecular Biology, Keio University School of Medicine, Tokyo 160-8582, Japan; Japan Science and Technology Agency (JST), Precursory Research for Embryonic Science and Technology (PRESTO), Saitama, Japan; School of Life Science and Technology, Tokyo Institute of Technology, Kanagawa 226-8501, Japan; Department of Molecular Biology, Keio University School of Medicine, Tokyo 160-8582, Japan; Graduate School of Science, The University of Tokyo, Tokyo 113-0032, Japan; Department of Molecular Biology, Keio University School of Medicine, Tokyo 160-8582, Japan; National Institute of Genetics, Mishima 411-8540, Japan; Department of Molecular Biology, Keio University School of Medicine, Tokyo 160-8582, Japan; National Institute of Genetics, Mishima 411-8540, Japan; Department of Molecular Biology, Keio University School of Medicine, Tokyo 160-8582, Japan; School of Life Science and Technology, Tokyo Institute of Technology, Kanagawa 226-8501, Japan; Department of Molecular Biology, Keio University School of Medicine, Tokyo 160-8582, Japan; National Institute of Genetics, Mishima 411-8540, Japan; Department of Biosciences and Informatics, Keio University, Yokohama 223-8522, Japan; National Institute of Genetics, Mishima 411-8540, Japan; School of Life Science and Technology, Tokyo Institute of Technology, Kanagawa 226-8501, Japan; Graduate School of Science, The University of Tokyo, Tokyo 113-0032, Japan; Department of Computational Biology and Medical Sciences, Graduate School of Frontier Sciences, The University of Tokyo, Tokyo 113-0032, Japan; Department of Molecular Biology, Keio University School of Medicine, Tokyo 160-8582, Japan

## Abstract

In animal gonads, transposable elements are actively repressed to preserve genome integrity through the PIWI-interacting RNA (piRNA) pathway. In mice, piRNAs are abundantly expressed in male germ cells, and form effector complexes with three distinct PIWIs. The depletion of individual *Piwi* genes causes male-specific sterility with no discernible phenotype in female mice. Unlike mice, most other mammals have four PIWI genes, some of which are expressed in the ovary. Here, purification of PIWI complexes from oocytes of the golden hamster revealed that the size of the PIWIL1-associated piRNAs changed during oocyte maturation. In contrast, PIWIL3, an ovary-specific PIWI in most mammals, associates with short piRNAs only in metaphase II oocytes, which coincides with intense phosphorylation of the protein. An improved high-quality genome assembly and annotation revealed that PIWIL1- and PIWIL3-associated piRNAs appear to share the 5′-ends of common piRNA precursors and are mostly derived from unannotated sequences with a diminished contribution from TE-derived sequences, most of which correspond to endogenous retroviruses. Our findings show the complex and dynamic nature of biogenesis of piRNAs in hamster oocytes, and together with the new genome sequence generated, serve as the foundation for developing useful models to study the piRNA pathway in mammalian oocytes.

## INTRODUCTION

Transposition of mobile DNA elements can cause severe damage by disrupting either the structural or regulatory regions on the host genome (1–3). To avoid such detrimental effects, many animals have a conserved adaptive immune system known as the piRNA pathway in gonads (4,5). piRNAs form effector complexes with PIWI proteins, a germline-specific class of Argonaute proteins, to guide recognition through complementary base-pairing, leading to silencing their target transposable elements (TEs) mainly in two ways: post-transcriptional silencing by PIWI-mediated cleavage of target transcripts in the cytoplasm and transcriptional silencing by PIWI-mediated chromatin modifications on target loci. Mutations in genes involved in the pathway can lead to sterility.

Although the mechanisms for generating piRNAs appear to largely differ among animals, the defined characteristics of piRNAs include a predominant length of 26–32 nucleotides (nt), a strong bias for uracil (U) at the 5′-ends (1U-bias), 2′-*O*-methylation of the 3′-ends, and clustering of reads to distinct genomic locations (4,5). The characterization of the piRNA populations in *Drosophila* and mouse has led to two models for the biogenesis mechanisms: the ping-pong cycle (6,7) and phased (8,9), which are intimately connected. Long single-stranded precursors, often >10 kb in size, are derived from discrete genomic loci that are now referred to as piRNA clusters or piRNA genes (6,10–13). Majority of piRNAs are derived from intergenic clusters. Intergenic piRNA clusters are often located in the heterochromatic regions and comprise various types of TEs that tend to be young and potentially active, suggesting that intergenic piRNA clusters provide the host with acquired and heritable memory systems to repress TEs (6,14). Some piRNAs also arise from 3′ untranslated regions (UTRs) of the protein-coding genes (15,16). The function of the genic piRNAs is not well understood, but some genic piRNAs show significant complementarity to protein-coding genes (15,17).

piRNA precursors are cleaved by either endonuclease Zucchini/MitoPLD or by the Slicer activity of PIWIs with pre-existing piRNAs to produce 5′ monophosphorylated piRNA intermediates that are loaded onto PIWI proteins (6–9,18–20). PIWI–piRNA complexes then initiate piRNA production that is formed by an amplification loop termed the ping-pong cycle in which reciprocal cleavage of TE and cluster transcripts by PIWI proteins generates new piRNA 5′-ends and amplifies piRNA populations while destroying TE mRNAs in the cytoplasm. The ping-pong cycle produces two classes of piRNAs overlapped by precisely 10 nt at their 5′-ends: one class shows a strong preference for U at their 5′-ends (1U) and the second class shows a preference for adenine at nucleotide 10 (10A). The ping-pong cycle is then accompanied by the phased production of piRNAs downstream of the cleavage site, which further creates a sequence diversity of piRNAs. The 3′-ends of piRNAs are determined either by direct cleavage of Zucchini/MitoPLD (mouse *Zucchini* homolog) or PIWIs or by trimming piRNA intermediates by resecting enzymes (Nibbler in *Drosophila*, Trimmer in silkworm, and PNLDC1 in mouse) (21–23). piRNA biogenesis is then concluded with 2′-*O*-methylation of the 3′-ends by HENMT1 methylase, which has been hypothesized to stabilize mature piRNAs (24–26). The extent of 3′-end cleaving/trimming and consequently piRNA length is determined by the footprint of the PIWI protein, possibly explaining the different size profiles of piRNAs associated with distinct PIWI proteins. Structural studies have shown that the 5′- and 3′-ends of the guide small RNAs, including piRNAs, are recognized by the MID-PIWI and PAZ domains of Argonaute/PIWI proteins, respectively (27–29).

Mammalian PIWI–piRNA pathways have been studied mainly in mice (5). Similar to *Drosophila*, mice express three PIWI proteins, MIWI (PIWIL1), MILI (PIWIL2) and MIWI2 (PIWIL4) in the testis, with varying spatiotemporal expression patterns during spermatogenesis. The non-redundant role of *Piwi* genes in the mouse testis is demonstrated by the fact that depletion of individual *Piwi* genes causes male-specific sterility owing to severe defects in sperm formation (30–32). Each PIWI protein associates with distinct piRNA populations; fetal prepachytene piRNAs and pachytene piRNAs. Prepachytene piRNAs are formed from TE- and other repeat-derived sequences. In contrast, pachytene piRNAs have a higher proportion of unannotated sequences with the diminished contribution of TE sequences, though they still function in TE silencing by guiding MILI and MIWI to cleave TE transcripts (33,34). A specific transcriptional factor, A-MYB, binds the promoter regions of pachytene piRNA clusters as well as core piRNA biogenesis factors, including MIWI, and initiates their transcription (35). Some pachytene piRNA clusters are divergently transcribed from bidirectional A-Myb-binding promoters (35).

Although PIWI genes in the fly and zebrafish are expressed in both testes and ovaries, mouse *Piwi* genes are expressed only weakly, if not at all, in the ovary, and depletion of these *Piwi* genes does not affect the female germline. Thus, these findings led to the assumption that the piRNA pathway does not play a role in mammalian oogenesis. Unlike mice, many other mammals possess four distinct *PIWI* genes (*PIWIL1*–*4*), suggesting that piRNA-mediated silencing may differ between mice and mammals with four *PIWI* genes (36,37). However, except for mice, little is known about mammalian piRNA pathways, particularly their roles in ovaries. Although recent studies have reported the presence of piRNA-like molecules in mammalian female germ cells, including humans (38–41), it is not yet known whether they play a role in the ovary because of the difficulty of technical and/or ethical application of genetic analysis. Although mice and rats belong to the *Muridae* family of rodents, both of which lack *Piwil3*, the golden Syrian hamster (golden hamster, *Mesocricetus auratus*) belongs to the *Cricetidae* family and has four PIWI genes. Golden hamsters have been used as an experimental rodent model for studying human diseases, particularly for cancer development and many different infectious diseases including COVID-19, because they display many features that resemble the physiological and pharmacological responses of humans (42,43). In addition, methods for manipulating gene expression, including the CRISPR/Cas9 system, have been recently employed in golden hamsters (44,45). Herein, we analyzed PIWI-associated piRNAs in oocytes and early embryos of golden hamsters, in the hope of applying genetic analysis to the piRNA pathway in the ovary. Our analyses revealed that the size of PIWIL1-associated piRNAs changes during oocyte maturation and that PIWIL3 binds short piRNAs only at the metaphase II (MII) stage of the oocyte, which coincides with phosphorylation of the protein. With an improved high-quality genome assembly and annotation of golden hamster, we showed that PIWIL1- and PIWIL3-associated piRNAs appear to share their 5′-ends. Their contents are similar to those observed with pachytene piRNAs in the mouse testis, but their targets in oocytes are mostly endogenous retroviruses. We further identified ovarian piRNA clusters, and motif search for the transcription start site regions of the piRNA clusters revealed no distinct binding motifs in their upstream regions, although A-Myb-binding motifs were enriched in the upstream regions of the testis piRNA clusters. Our study provides a basis for understanding the roles of the piRNA pathway in mammalian oocytes.

## MATERIALS AND METHODS

### Animals and tissue collection

Ovaries were collected from 4-week-old golden hamsters that were obtained from the Sankyo Labo Service Corporation, Inc. MII oocytes were collected from 8-week-old golden hamsters that were injected with 40 U of equine chorionic gonadotropin (Serotropin; ASUKA Pharmaceutical Co., Ltd., Tokyo, Japan) at estrus. Two-cells were collected from 8-week-old golden hamsters that were injected with equine chorionic gonadotropin at estrus and mated with adult male hamsters.

### 5′ RACE

Total RNA for *PIWIL3* 5′ RACE was extracted from the ovaries of 8-week-old golden hamsters using ISOGEN (Nippon Gene) and RNeasy (Qiagen). 5′ RACE was performed using the SMARTer RACE 5/3 kit (Takara Bio) according to the manufacturer's instructions. The PCR-amplified PIWIL3 5′ RACE fragments were cloned into the pRACE vector and sequenced.

### Construction of golden hamster PIWI expression vector

To construct the golden hamster *PIWI* gene expression vectors, *PIWI* genes were amplified by PCR using gene specific primers and 3-week-old hamster testis and ovary cDNA. The PCR products were digested with SpeI and NotI, and then cloned into pEF4–3xFlag vector.

### Generation of anti-hamster PIWIL3 monoclonal antibodies

Anti-PIWI monoclonal antibodies were produced as described previously (46,47) with some modifications. Monoclonal mouse antibodies against hamster PIWIL3 were generated by injecting mice with glutathione S-transferase (GST)-hamster PIWIL3 (20–260 amino acids from the N-terminal where contains repeat region) and then fusing lymph node and spleen cells with the myeloma cell line P3U1 by GenomONE™-CF EX Sendai virus (HVJ) Envelope Cell Fusion Kit (Ishihara Sangyo) to produce hybridomas. Polyclonal antibodies were selected using ELISA, immunostaining, western blotting, and immunoprecipitation. The hybridoma clone 3E12 used in this study is available for all these applications.

### Western blotting

Western blot analysis was performed as described previously (48). One-tenth of protein from ovaries, proteins from 15 oocytes and 2-cell embryos and one-tenth of protein from the testes were subjected to SDS-PAGE. Culture supernatants of anti-marmoset PIWIL1 (MARWI) (1A5-A7) hybridoma cells (37) and anti-hamster PIWIL3 (3E12) hybridoma cells (1:500) and mouse monoclonal anti-β-tubulin (1:5000, DSHB, E7) were used.

### Immunofluorescence

The ovaries from 8-week-old wild type female golden hamsters were fixed with 4% paraformaldehyde and embeded in paraffin. Paraffin blocks were sliced to 5 μm and treated with an anti-PIWIL1 monoclonal antibody (1A5) or anti-PIWIL3 monoclonal antibody (3E12). An Alexa488-conjugated anti-mouse IgG (Molecular Probes) was used as the secondary antibody. Fluorescence was observed with an IX72 fluorescence microscope (Olympus).

### Immunoprecipitation

Immunoprecipitation details have been described previously (15). Briefly, the ovaries were homogenized using TissueLyser II (QIAGEN) and oocytes and 2-cell embryos in which the zona pellucida were eliminated using Acidic Tyrode’s solution with 0.01% polyvinyl alcohol (PVA) and homogenized by pipetting in binding buffer (30 mM HEPES, pH 7.3, 150 mM potassium acetate, 2 mM magnesium acetate, 5 mM dithiothreitol (DTT), 1% Nonidet *P*-40 (Calbiochem, 492016), 2 mg/ml Leupeptin (Sigma, L9783), 2 mg/ml Pepstatin (Sigma, P5718), and 0.5% Aprotinin (Sigma, A6279)). PIWIL1 and PIWIL3 proteins were immunopurified using anti-MARWI (1A5) and anti-hamster PIWIL3 (3E12) immobilized on Dynabeads protein G (Life Technologies, 10004D) and anti-mouse IgG (IBL, 17314) were used as non-immune, negative controls. The reaction mixtures were incubated at 4°C overnight and the beads were rinsed three times with binding buffer.

### β-elimination

Periodate oxidation and β-elimination were performed as described previously (25,26,37,49,50). A 100 μl mixture consisting of purified RNAs and 10 mM NaIO4 (Wako, 199-02401) was incubated at 4°C for 40 min in the dark. The oxidized RNAs were then incubated with 1 M Lys-HCl at 45°C for 90 min to achieve β-elimination.

### ^32^P-labeling

The 5′-end of the RNAs were labeled with [gamma-^32^P] ATP (Perkin Elmer) and T4 polynucleotide kinase (ATP: 5-dephosphopolynucleotide 5′-phosphotransferase, EC 2.7.1.78). The labeled RNAs were cleaned using Micro Bio-Spin™ column 30 (Bio-Rad) and ethanol precipitation. The precipitated RNAs were subjected to SDS-PAGE with 15% urea.

### Summary of genome sequence and assembly of the golden hamster

Raw PacBio reads (Supplementary Table S1) were assembled into contigs using the FALCON genome assembler, which is widely used for processing long reads (51). To correct assembly errors in the FALCON contigs, we applied the Racon module (52) three times. To obtain a chromosome-scale genome assembly, we aligned all contigs to the 22 golden hamster chromosomes (MesAur1.0_HiC, DNA Zoo) (Supplementary Figure S7). We attempted to fill 3797 gaps in the reference chromosomes using the FALCON contigs and error-corrected reads. We generated error-corrected reads using the FALCON assembler, which aligned PacBio raw reads to each other, obtained the consensus sequence of aligned reads using multiple alignments, and then output the consensus sequences as error-corrected reads. To fill unsettled gaps, we aligned error-corrected reads to the gaps using the minimap2 program (53) and manually inspected the results to determine whether the gaps were spanned by more than one error-corrected read (Supplementary Table S2). Finally, we polished the draft assembly using the PacBio raw reads and Arrow program.

### PacBio read assembly

We used the FALCON genome assembler version 2018.08.08-21.41-py2.7-ucs4-beta (51) with default parameter settings to assemble PacBio reads. To correct errors in the assembly, we applied the RACON module version 1.4.10 (52) three times with default parameter settings.

### Genome assembly alignment

We aligned all contigs in the assemblies to the golden hamster reference assembly (DNA Zoo release MesAur1.0_HiC.fasta.gz) using MUMmer 4.0.0beta2 software (54) with the nucmer program, and the following parameters: mum min cluster = 100, max gap = 300. We also used the minimap2 version 2.13 (53) with default parameter settings.

### Effects of the Arrow program on the draft genome

We polished the draft genome using the Arrow program version 2.3.3 (https://github.com/PacificBiosciences/GenomicConsensus) with default parameter settings. We used the QUAST 5.0.2 tool (55) to calculate mismatch and indel ratios for our golden hamster genome with respect to the DNA Zoo Hi-C genome, both before and after genome polishing (Supplementary Table S3).

### Gene lift over from N2 to VC2010

We lifted gene structures and other genome annotations from the golden hamster reference assembly (MesAur1.0 release 100) to our golden hamster assembly. Such cross-assembly mapping typically requires an annotation file in a standard format (e.g. GFF3; https://github.com/The-Sequence-Ontology/Specifications/blob/master/gff3.md), chain alignment (56), and a program capable of mapping annotations based on chain alignment (e.g. liftOver) (57). The reference genome sequence was downloaded (ftp://ftp.ensembl.org/pub/release-100/fasta/mesocricetus_auratus/dna/Mesocricetus_auratus.MesAur1.0.dna.toplevel.fa.gz) as were the annotations of canonical golden hamster genes (ftp://ftp.ensembl.org/pub/release-100/gff3/mesocricetus_auratus/Mesocricetus_auratus.MesAur1.0.100.gff3.gz). Both the genome sequence and its annotations were obtained from the Ensembl database (release-100). To chain-align our golden hamster assembly (as the query) to the reference assembly (as the target), we used methods almost identical to those described by the University of California Santa Cruz (UCSC) for same-species genomic chain alignment (http://genomewiki.ucsc.edu/index.php/DoSameSpeciesLiftOver.pl). The liftOver protocol required several utility programs from UCSC, some of which were downloaded as precompiled binaries (http://hgdownload.cse.ucsc.edu/admin/exe/linux.x86_64). To map genome annotations, we used liftOver with the parameter -gff -minMatch = 0.90.

### Comparative genome analysis

We compared our golden hamster genome with the mouse reference genome (*Mus musculus* GRCm38.p6 release-100 in the Ensembl database; ftp://ftp.ensembl.org/pub/release-100/fasta/mus_musculus/dna/) and the rat reference genome (*Rattus norvegicus* Rnor_6.0 release-100 in the Ensembl database; ftp://ftp.ensembl.org/pub/release-100/fasta/rattus_norvegicus/dna/) using the Murasaki program (ver. 1.6.8) with a seed pattern weight of 76 and a length of 110 (58).

### Chromosomal evolution in Rodentia

First, we identified synteny blocks that were shared between two or three species, that is, hamster, mouse, and/or rat (details provided in Supplementary Figure S8). Next, we inferred ancestral karyotypes of *Muridae* and *Cricetidae* (Figure 3B) by integrating synteny blocks according to maximum parsimony, minimizing the total number of chromosomal rearrangements such as chromosome fusions, chromosome fissions, and translocations. We excluded inversions from chromosomal rearrangements, which were markedly more prevalent than the other rearrangements. We compared our nearly complete golden hamster genome with the mouse (*Mus musculus*) sand rat (*Rattus norvegicus*) reference genomes. Figure 4A shows conserved synteny blocks between the golden hamster, mouse, and rat genomes. We inferred ancestral karyotypes by integrating synteny blocks shared between two or three species according to maximum parsimony to minimize the amount of chromosomal rearrangement. We obtained a high-resolution ancestral karyotype of *Muridae*, including mice and rats, using the golden hamster genome as the outgroup as well as a species ancestral *Cricetidae* karyotype (Figure 3B).

### Characterization of TEs in the hamster genome

We first used the RepeatModeler ver. 2.0 (59) coupled with LTR_retriever ver. 2.8 (60) for *de novo* identification of repetitive elements in the hamster genome. Among the initial repeat candidates obtained, 282 elements, covering ∼37% of the genome in total, were used for subsequent in-depth characterization. In the accurate identification process, we conducted a BLASTN search and collected 80–100 sequences along with the 6-kbp flanking sequences of each element. The sequences were aligned with MAFFT ver. 7.427 (61) followed by manual curation, and a refined consensus sequence was constructed to be used for the next round of blastn search. This process was repeated for three rounds at maximum until the consensus sequence reached the 5′ and 3′ termini. We finally constructed 177 consensus sequences at the subfamily level and classified them based on the sequence structure and a comparison with known elements using CENSOR (62) and RepeatMasker ver. 4.1.0. We finally constructed a custom repeat library by adding 177 novel consensus sequences to the original rodent repeat library. RepeatMasker analysis was conducted for genome annotation using the cross-match engine with the sensitive mode (-s). Young (i.e. recently active) complete TEs were selected based on the <5.0 K2P divergence from the consensus sequence and the full-length insertions, although ignoring the lack of sequence homology in up to 50 bp of the 5-terminal of LINEs and 3 bp at the edge of other TEs.

### Small RNA-seq library preparation

PIWIL1 and PIWIL3-associated piRNAs were prepared as described previously (37). PIWI family associated small RNAs were extracted from immunopurified proteins using ISOGEN (Nippon Gene, 311-02501). Libraries were prepared using NEBNext Small RNA Library Sample Prep Set [New England BioLabs (NEB), E7330] with slight modifications. Small RNAs obtained and purified using SPRIselect (Beckman Coulter, B23317) underwent three ligation at 16°C for 18 h, then free three adaptors were degraded using five deadenylase (NEB, M0331) and RecJf (NEB, M0264). The 3′-ligated RNAs underwent five adaptor ligation at 25°C for 1 h. The RNAs were reverse-transcribed using SuperScriptIII (Life Technologies, 18080-044) and were PCR-amplified using Q5 Hot Start High-Fidelity DNA Polymerase (NEB, M0493) with 18 cycles.

### Small RNA sequencing and data processing

PIWIL1 and PIWIL3-associated small RNAs were sequenced using MiSeq (Illumina) with three replicates from different samples. A total of 32 478 862 reads were obtained and processed as described previously (63). The adaptors were trimmed from the reads using Cutadapt version 2.10 (64). The replicates were highly correlated (*R*^2^ ≧ 0.9), so the reads were merged. Each read was mapped to the reference genome (hamster.sequel.draft-20200302.arrow.fasta) using Bowtie version 1.2.3 (65) with the -v 0 option, which extracts the small RNA reads that were perfectly mapped. Genome mapped reads were selected by size using Seqkit(66). The PIWIL1-piRNAs were divided into two groups: Oocyte Short (OoS) group, which included 21–27 nt RNAs, and Oocyte Long (OoL) group with 28–31 nt RNAs. PIWIL3-piRNAs with 18–20 nt were selected for further analysis.

### RNA-seq data processing

The RNA-seq library in oocytes was prepared using the SMART-Seq® Stranded Kit (TaKaRa, 634442). Total RNAs obtained using NucleoSpin® RNA Plus XS (TaKaRa, 740990) from approximately 100 oocytes were fragmented at 85°C for 6 min. Sheared RNAs were processed under the ‘Low input category.’ PCR1 was performed with 5 cycles, followed by PCR2 with 13 cycles, and the final cleanup was performed once. RNA-seq libraries in the ovary and testis were prepared using the TruSeq Stranded mRNA Sample Prep kit. The libraries were sequenced using HiSeq2000 (Illumina) and the obtained reads were combined, resulting in a total of 79 152 300 pair-end reads for hamster testis and 81 979 150 pair-end reads for hamster ovary, respectively. Reads with trimming adaptors and quality filtering were mapped to the hamster reference genome (hamster.sequel.draft-20200302.arrow.fasta) using hisat2 version 2.2.0 (67) with the strandness option (–strandness FR). To calculate transcripts per kilobase million mapped (TPM) as expression levels of genes, we used StringTie version 2.1.3 (68).

### Sequence logo

Sequence logos were generated using the motifStack R package (http://www.bioconductor.org/packages/release/bioc/html/motifStack.html). Small RNA sequences were aligned at the 5′-end, and nucleotide bias was calculated per position.

### Annotation of reads

Annotation of genome mapped reads was determined as described previously (63) with some modifications. We examined the overlap between read-mapped genomic regions and feature track data. Each feature data was obtained from RpeatMasker (http://www.repeatmasker.org/) for transposons, repeats, tRNAs, rRNAs and snoRNAs, miRDeep2 version 2.0.1.2 (69) for miRNAs and UCSC LiftOff pipeline for protein-coding genes. Reads were assigned to a feature when the length of its overlap was longer than 90% of the small RNA. The priority of the feature assignment was defined to avoid any conflict of the assignment. For this study, the priority was in the following order: transposon, repeat, miRNA, rRNA, tRNA, snRNA, snoRNA and protein-coding genes (exons and introns). Any unassigned regions were regarded as unannotated regions.

### piRNA target TE prediction

Prediction of PIWI-piRNA targets derived from TE regions was determined as described previously (37,63) with some modifications. First, we eliminated piRNA reads that mapped to tRNAs or rRNAs. The extracted reads were aligned to consensus sequences of transposons (Rodentia custom library), allowing two mismatches. The alignment was performed using Bowtie because of the large number of obtained sequences.

### piRNA cluster prediction

Prediction of piRNA clusters was performed using proTRAC version 2.4.3 (70) under the following conditions as described previously (40): (i) >75% of the reads that were appropriate to the length of each piRNA; (ii) >75% of the reads exhibited the 1 U or 10 A preference; (iii) >75% of reads were derived from the main strand and (iv) -pimin option with 21, 28 and 18 for oocyte PIWIL1-piRNAs and PIWIL3-piRNAs, respectively.

### Motif search at the transcriptional start site of piRNA clusters

We analyzed the motif sites surrounding the transcription start sites of the testis and ovary piRNA clusters. We first extracted sequences surrounding transcriptional start sites of unidirectional and bidirectional piRNA clusters predicted from small RNA-seq and RNA-seq mapping data. For the detection of bidirectional piRNA clusters, we first determined the number of reads per base in the cluster based on the results of RNA-seq with TopHat version 2.1.1 (71). We next detected the direction of each base site and a region in which the same direction was contiguous by more than 200 bp was identified. If (+) or (−) occupies >75% of the cluster, the cluster is designated as the direction. If the number of reads was less than five, the direction was the same as the previous base site. The region between the switch of transcriptional direction was extracted along with 200bp upstream and downstream regions, as transcriptional start site of bidirectional clusters. The bidirectional cluster with multiple switching regions identified using these criteria was omitted. For the detection of unidirectional piRNA clusters, we extracted 300 bp upstream and 200 bp downstream of genomic regions where piRNA clusters overlapped with the transcript regions detected from Cufflinks version 2.2.1 (72). The direction of the sequence strands was the same as in the transcripts. We then used these sequences and performed motif searches using MEME version.5.1.0 (73). Tomtom version 5.1.1 (73) was used for motif comparison.

### Visualization of sequenced reads

To visualize the read density obtained from smRNA-seq snd RNA-seq, we created a BigWig file by using HOMER version 4.11 (74) and displayed the Integrative Genomics Viewer (IGV) version 2.4.1. (75). The normalized expression level of each sample was calculated using reads per million reads (RPM).

## RESULTS

### *PIWI* genes are expressed in the oocyte of the golden hamster

**Figure 1.**
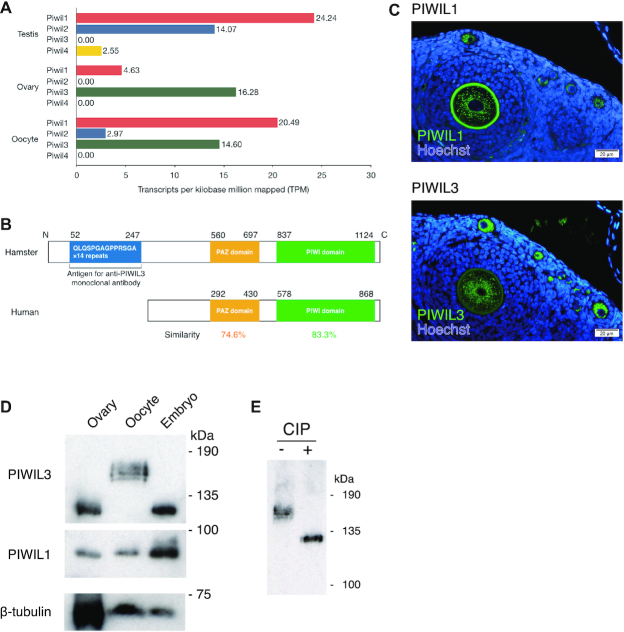
*PIWI*s are expressed in hamster oocytes. (**A**) Expression patterns of *PIWI* families in male and female hamsters. Transcriptome data were obtained from Illumina HiSeq2000. The x-axis suggests the normalized expression level (TPM). (**B**) Protein homology of PIWIL3 in hamster and human. Approximately 74.6% of the PAZ domain and 83.3% of the PIWI domain in hamster PIWIL3 are conserved in human PIWIL3. The large extension of the amino-terminal portion of the ORF, which contains 14 repeats of nucleotide sequences encoding the amino acid sequences QLQSPGAGPPRSGA is presented in hamster PIWIL3. (**C**) PIWIL1 (upper panel) and PIWIL3 (lower panel) levels from an ovary. Immunostaining with PIWIL1 and PIWIL3 are shown: (green) PIWIL1 and PIWIL3; (blue) Hoechst. The signal in the zona pellucida at upper panel is the autofluorescence. (**D**) Western blotting was performed from hamster whole ovaries, MII oocytes, and 2-cell embryos with anti-PIWIL3, anti-PIWIL1, and anti-β-TUBULIN antibodies. The size of the PIWIL3 protein largely shifted by approximately 40 kDa in MII oocytes. The anti-MARWI (PIWIL1) antibody (1A5) and anti-PIWIL3 antibody (3E12) detected a single band in each sample except for MII oocytes. (**E**) Western blotting was performed from hamster MII oocytes with (+) or without (−) CIP treatment. A discrete band migrated to the estimated molecular weight of 130 kDa in the CIP-treated sample, demonstrating that PIWIL3 is heavily phosphorylated in MII oocytes.

To examine the expression of *PIWI* genes in golden hamster gonads, we performed RNA-sequencing (RNA-seq) analysis using hamster ovary, oocyte, and testis samples and analyzed the expression level of *PIWI* genes by calculating transcripts per kilobase million mapped (TPM) (Figure 1A). The estimated expression levels of *Piwil1* and *Piwil2* were relatively high in the testis (TPM = 24.24 and TPM = 14.07, respectively). *Piwil1* was also expressed in the ovary (TPM = 4.63), while *Piwil2* is not detected in the whole ovary and appeared to be only weakly expressed in the oocyte. *Piwil4* appeared to be expressed only in the testis, consistent with previous transcriptome analysis in human oocytes (40). Interestingly, *Piwil3* was highly expressed in the oocyte (TPM = 14.60). In sharp contrast, the expression of *Piwil3* could not be detected in the testis. These results are consistent with previous analyses of bovine and human PIWIL3 (38,40). To analyze the expression levels of known PIWI–piRNA pathway factors other than PIWI genes, we also calculated the TPM values of the predicted homologous genes using RNA-seq data (Supplementary Figure S1A). Most of the known PIWI–piRNA pathway factor homologs, including *Mov10l1*, *Mael*, *MVH* (mouse *Vasa* homolog), *MitoPLD*, *Gtsf1*, *Henmt1* and Tudor domain families (4), were expressed in both testes and oocytes, suggesting that both testes and oocytes of hamsters have similar biogenesis pathways to produce piRNAs.

To confirm the expression of *PIWI*s in the ovary, we isolated their mRNAs from the ovary. Open reading frames (ORFs) of sequenced *Piwil1* and *Piwil2* cDNAs correspond well with the respective annotated gene products deposited in the Broad Institute database (MesAur1.0, Broad Institute data) (Supplementary Figure S1B). However, to our surprise, during the cloning of the *Piwil3* cDNA, we found that the large extension of the amino-terminal portion of the ORF contains 14 repeats of nucleotide sequences encoding the amino acid sequences QLQSPGAGPPRSGA (Figure 1B). To further confirm the expression of PIWIs in the ovary at the protein level, we produced specific monoclonal antibodies against PIWIL3 (Supplementary Figure S1C). We also found that a monoclonal antibody that recognizes marmoset PIWIL1 (37) cross-reacts with hamster PIWIL1 specifically among hamster PIWI proteins (Supplementary Figure S1C). Thus, we focused our analysis on PIWIL1 and PIWIL3. Immunostaining with the antibodies produced showed that both PIWIL1 and PIWIL3 were expressed in the cytoplasm of growing oocytes in the ovary (Figure 1C). Western blotting with anti-PIWIL1 antibody showed a discrete band at 90 kDa in the ovary, metaphase II (MII) oocytes, and 2-cell embryos (Figure 1D). Western blotting with anti-PIWIL3 antibody revealed a discrete band at 130 kDa in the ovary and 2-cell embryos, which is consistent with the calculated molecular weight of the protein with the large amino-terminal extension (Figure 1B and D). Intriguingly, the size of the protein largely shifted by approximately 40 kDa in MII oocytes (Figure 1D).

### PIWIL3 protein is highly phosphorylated in metaphase II oocytes

This large increase of the PIWIL3 protein in size, together with the broad and fuzzy nature of the band on the gel (Figure 1D), prompted us to test whether this size shift could be due to modification of the protein with phosphorylation. Treatment of MII oocyte lysates with calf intestinal alkaline phosphatase (CIP), an enzyme known to dephosphorylate proteins (76), caused a discrete band to migrate to the estimated molecular weight of 130 kDa, demonstrating that PIWIL3 is heavily phosphorylated in MII oocytes (Figure 1E). These results show that PIWIL1 and PIWIL3 are expressed in growing oocytes in the ovary as well as in early embryos and that PIWIL3 is modified with phosphorylation specifically at the MII stage of oocytes. Since the mouse genome lacks *Piwil3* and thus the characterization of PIWIL3 protein has been delayed, our findings indicate that *Piwil3* may have specific functions in female gonads.

### The size of piRNAs loaded onto PIWIL1 changes during oocyte maturation

**Figure 2.**
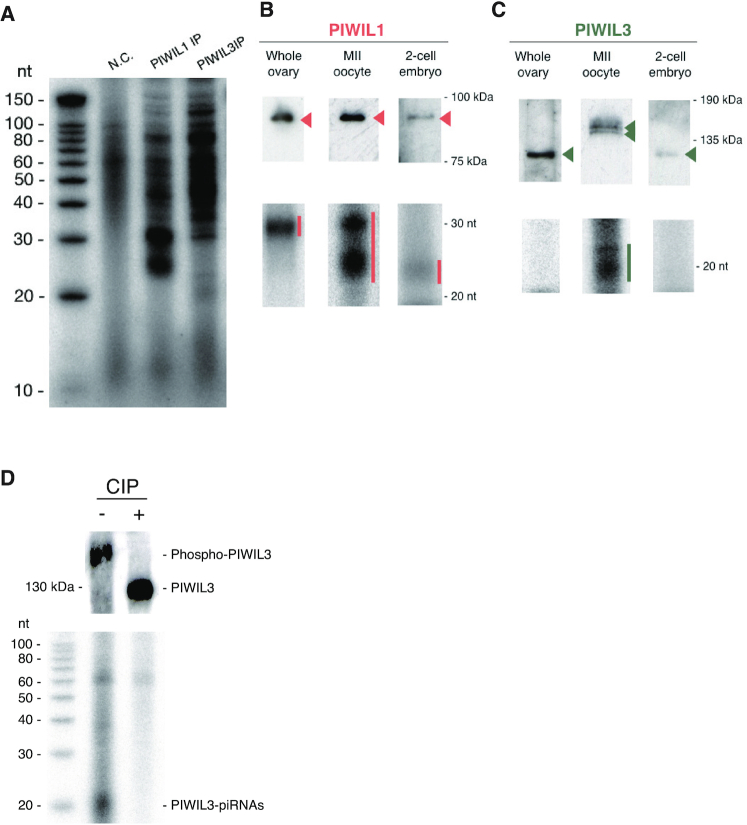
The different populations of piRNAs associate with each of the PIWI proteins at different developmental stages. (**A**) Isolated RNAs from PIWIL1 and PIWIL3 immunoprecipitates in MII oocytes were ^32^P-labeled and separated by a denaturing polyacrylamide gel. PIWIL1 and PIWIL3 proteins associate with ∼28–31-nt and ∼18–20-nt-long piRNAs, respectively. (**B**) Western blotting and ^32^P-labeling were performed from PIWIL1 immunoprecipitates in hamster whole ovaries, MII oocytes, and 2-cell embryos. Upper panel: Western blotting; lower panel: ^32^P-labeling. (**C**) The same experiments with (**B**) were performed from PIWIL3 immunoprecipitates. (**D**) Phosphorylation of the protein is required for PIWIL3 probably either to get loaded with piRNAs or hold them or both. PIWIL3 immunoprecipitates were treated with (+) or without (−) CIP. Upper panel: Western blotting; lower panel: ^32^P -labeling of RNAs, which were purified from each PIWIL3 immunoprecipitate.

To isolate PIWIL1- and PIWIL3-associated small RNAs from oocytes, we immunopurified the associated complexes from MII oocytes with specific monoclonal antibodies produced. We then isolated RNAs, ^32^P-labeled them, and analyzed them using a denaturing polyacrylamide gel (Figure 2A). Intriguingly, PIWIL1 was associated with two populations of small RNAs: one with 29–30 nt and the other with 22–23 nt in MII oocytes. We then immunopurified PIWIL1-associated complexes from whole ovaries (including growing oocytes) and 2-cell embryos. The sizes of PIWIL1-associated piRNAs in whole ovaries and 2-cell embryos were 29–30 nt and 22–23 nt, respectively (Figure 2B). The resistance of PIWIL1-associated piRNAs in MII oocytes to periodate oxidation (NaIO4) and β-elimination reactions show that they are modified at the 3′ terminal nucleotide with a 2′-*O*-methyl marker (Supplementary Figure S2A). We also isolated PIWIL1-associated small RNAs from whole testes and found that small RNAs (29–30 nt) were loaded onto PIWIL1 in the testis (Supplementary Figure S2B). These results show that piRNAs loaded onto PIWIL1 change their sizes during oocyte maturation, from the size equivalent to that observed in the testis to a mixture of long and short populations, and short piRNAs (22–23 nt). To our knowledge, this is the first study to show that the size of piRNAs loaded onto a distinct PIWI protein changes during germline development.

### Phosphorylated PIWIL3 appears associated with short piRNAs only in MII oocytes

In sharp contrast, PIWIL3 was found to bind to a class of small RNAs with 19 nt (Figure 2A), which is consistent with the recent finding that human PIWIL3 associates with small RNAs (∼20 nt) in human oocytes (40). 19–20 nt RNAs in oocytes almost completely disappeared after β-elimination reactions, indicating that PIWIL3-associated piRNAs lack 2′-*O*-methylation at their 3′ terminal (Supplementary Figure S2C). This is also consistent with human PIWIL3-associated piRNAs (40). We failed to detect small RNAs associated with PIWIL3 in whole ovaries and 2-cell embryos (Figure 2C). With the caveat that this could be because of technical reasons for immunoprecipitation with the antibodies and/or the buffer conditions used, our findings suggest that PIWIL3 may bind piRNAs with 19 nt exclusively in MII oocytes but be freed from piRNAs as ‘empty’ PIWIL3 at the early stages of oocyte maturation and in early embryos. PIWIL3 is heavily phosphorylated only in MII oocytes, raising the possibility that phosphorylation of the protein may be required for the association with a class of short piRNAs. To test this, we performed immunoprecipitation with an anti-PIWIL3 antibody using MII oocyte lysate that had been treated with CIP and examined whether the CIP treatment affected the association of piRNAs with PIWIL3. Indeed, PIWIL3 treated with CIP was free from piRNAs (Figure 2D). This shows that phosphorylation of the protein is required for PIWIL3 probably either to get loaded with piRNAs or hold them or both.

### Generating the hamster genome assembled by resequencing the whole genome to accurately map piRNAs

Although the draft genome of the golden hamster has been sequenced (the MesAur1.0 genome), we soon came to realize that we needed much more accurate genome sequence data to further characterize these PIWI-associated piRNAs on the genome mainly because the MesAur1.0 genome sequence contains a large number of gaps (N) (17.58% of the genome; 420 Mb of the 2.4 Gb) and remains fragmented. Because TEs and other repeats in the genome are the main targets of piRNAs in many animals, the lack of accurate sequences of TEs and other repeats is a serious problem when mapping piRNAs on the genome. Accurate detection of TEs requires both full collection/classification of TE consensus sequences and high-quality genome assembly. Thus, we re-sequenced the golden hamster genome.

**Figure 3.**
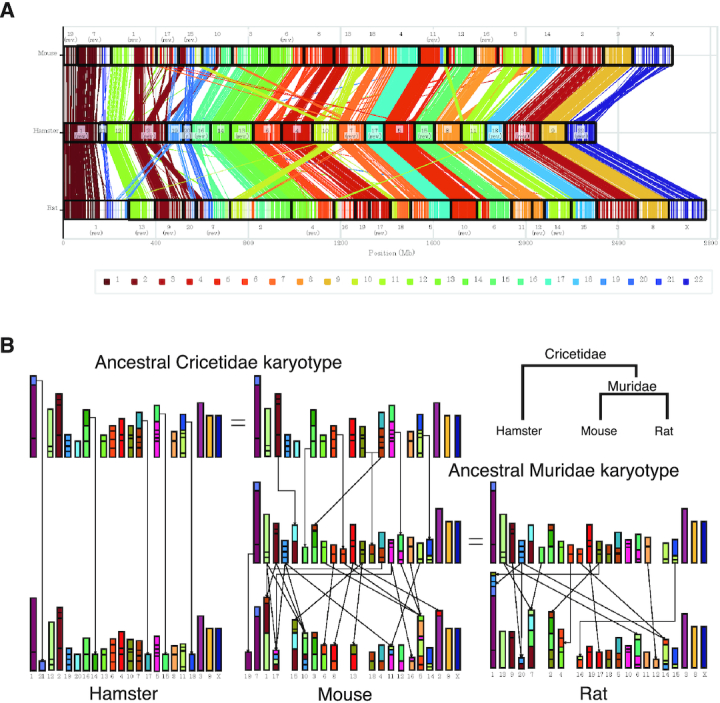
Chromosomal evolution in Rodentia. (**A**) Each line represents a reciprocally best-matching pair of positions in our hamster genome (middle) and the mouse (top) or rat (bottom) reference genome. In each line, the colored part represents the hamster chromosome, and the color palette displayed at the bottom shows the color-coding. Neighboring lines indicate synteny blocks conserved between two species. (**B**) Schematic showing the ancestral karyotypes of *Cricetidae* and *Muridae* inferred from conserved synteny blocks using maximum parsimony. The top two karyotypes are identical and represent the ancestral *Cricetidae* karyotype; the middle two show the ancestral *Muridae* karyotype. Each box represents a conserved synteny block among the three species; the colored part indicates the hamster chromosome as per Figure A. Small synteny blocks are enlarged. Lines from one proto-chromosome to the descendant chromosomes indicate rearrangements (fusion, fission, or translocation). To simplify the graph, we omitted lines between the identical chromosomes within the same columns. Brown blocks show the existence of a proto-chromosome in the ancestral *Cricetidae* karyotype. We confirmed the previous speculation that mouse chromosomes 5 and 16 obtained blocks from a common *Muridae* proto-chromosome (light-orange), and that chromosomes 10, 15 and 17 obtained blocks from a common *Cricetidat*e proto-chromosome (maroon blocks). We also identified two groups of mouse chromosomes (6, 8) and (8, 13) having large blocks from common *Muridae* proto-chromosomes.

Details of the hamster genome assembly are shown in the Methods section. The final genome assembly is summarized in Table 1. The nucleotide difference between the DNA Zoo MesAur1.0_HiC assembly and our assembly was 0.140%. We assessed the completeness of the genome assemblies using the BUSCO tool (77) and found that our golden hamster genome assembly included 3,991 complete genes (97.2%) and 37 fragmented genes among 4,104 single-copy genes. Our new golden hamster genome allowed us to resolve several issues that had remained ambiguous. For example, although putative ancestral karyotypes of rodents in the *Muridae* and *Cricetidae* families have been partly reconstructed by traditional chromosome painting (78), we compared our nearly complete genome with the mouse (*M. musculus*) and rat (*R. norvegicus*) reference genomes (Methods) and identified conserved synteny blocks between the golden hamster, mouse, and rat genomes (Figure 3A). We inferred ancestral karyotypes by integrating synteny blocks shared between two or three species according to maximum parsimony, to minimize the amount of chromosomal rearrangement (Methods) and obtained a high-resolution ancestral karyotype of *Muridae* using the golden hamster genome as the outgroup as well as a precise ancestral *Cricetidae* karyotype (Figure 3B). Although our ancestral *Cricetidae* and *Muridae* karyotypes were consistent with most previous inference (78), we resolved some problems: for example, it was unclear whether mouse chromosomes 3 and 4 possessed synteny blocks from a common ancestral karyotype, and our analysis demonstrated the existence of a proto-chromosome in the ancestral *Cricetidae* karyotype (brown blocks in Figure 3B). We also confirmed previous speculation that mouse chromosomes 5 and 16 obtained blocks from a common *Muridae* proto-chromosome (light orange in Figure 3B), and that chromosomes 10, 15, and 17 obtained blocks from a common *Cricetidae* proto-chromosome (maroon blocks in Figure 3B). Finally, we identified two groups of mouse chromosomes (6,8) and (8,13) having large blocks from common *Muridae* proto-chromosomes.

**Table 1.** The statistics of the genome assemblies

	Ensembl reference (MesAur1.0)		DNA zoo (Hi-C, MesAur1.0_HiC)		Our draft genome	
Number of scaffolds	21 484		22		22	
**Total bases**	**2 504 925 039**		**2 351 684 983**		**2 302 785 321**	
Max length	79 790 405		158 839 776		155 812 073	
Min length	1000		29 446 437		29 820 714	
Average length	116 595		106 894 772		104 672 060	
N10	30 845 043	5	158 364 997	2	155 489 353	2
N20	24 332 566	15	157 194 130	3	153 247 738	3
N30	19 995 379	26	133 100 194	5	130 365 707	5
N40	16 574 428	39	124 260 332	7	122 808 942	7
**N50**	**12 753 307**	57	**119 733 378**	9	**116 281 491**	9
N60	10 749 540	79	111 148 667	11	108 846 491	11
N70	8 492 120	105	107 481 000	13	105 528 230	13
N80	5 360 393	141	87 854 995	16	85 318 168	16
N90	2 084 661	212	85 557 910	18	83 767 106	18
N99	3663	9798	29 446 437	22	29 820 714	22
Number of gaps	216 216		199 141		594	
**Total bases of gaps**	**428 748 785**	**17%**	**367 776 424**	**16%**	**5 784 938**	**0.25%**
Max length of gaps	43 810		43 172		48 182	
Average length of gaps	1983		1847		9739	
Number of unanchored contigs			21 762		3775	
Total bases			141 734 268		234 152 039	
BUSCO complete			3671/4104	89.4%	3 991/4104	97.2%
BUSCO fragmented			3722/4104	90.7%	4 028/4104	98.1%
MesAur1.0_HiC identity (%)						99.86%

Using our genome assembly, we compared the hamster genomic locus containing the *Piwil3* gene with syntenic regions of the mouse and rat genomes. The *Piwil3* gene is flanked by *Wscd2* and *Sgsm1* in the hamster genome (Supplementary Figure S3A). The order of the two genes is conserved in the syntenic regions of the mouse and rat genomes. We then extracted the genomic regions between these genes from our hamster genome and the reference genomes of mouse (mm10) and rat (rn6), compared the syntenic regions using dot plots (Supplementary Figure S3B), and observed the absence of the *Piwil3* gene. The validity of the hamster genomic region with *Piwil3* was confirmed by checking each base in the region was covered by an ample number of long reads (Supplementary Figure S3C). In addition, we performed a similarity search with blastn and ssearch36 using the protein-coding sequence (CDS) of hamster *Piwil3* as a query and found no hits in the corresponding regions of the mouse and rat genome. The *PIWIL3* gene is conserved in most mammals, including humans, suggesting that *Piwil3* might have been deleted after speciation in mice and rats.

With the new golden hamster genome sequence generated, we also conducted a *de novo* repeat characterization and identified 177 consensus sequences of repetitive elements at the subfamily level, including 3 SINEs, 12 LINEs, 156 long terminal repeat (LTR) retrotransposons and 2 DNA transposons. RepeatMasker analysis using our custom repeat library (RepeatMasker rodent library with newly identified 177 consensus sequences) revealed that SINEs, LINEs, LTR retrotransposons, and DNA transposons occupy 9.2%, 16.9%, 12.1% and 1.3% of the hamster genome, respectively. The contents of TEs are equivalent to those found in mice and rats, but the fractions of SINE and LINE are, respectively, higher and slightly lower in the hamster than those observed in mice and rats (Table 2). The contents of the MesAur1.0 genome (MesAur1.0_HiC) were similarly analyzed. In contrast to our assembly, a much lower proportion of young TEs were detected even using our custom repeat library (Supplementary Figure S4A, Supplementary Table S4). This is mostly because of the high frequency of gaps (Ns) in the MesAur1.0_HiC assembly (Table 1), which resulted in apparently less similarity between TEs and their consensus sequences.

**Table 2.** Proportion (%) of transposable elements in rodents

Class	Clade/superfamily	Hamster	Mouse	Rat
SINE	B1	3.3	2.32	1.56
	B2	1.96	2.16	2.1
	B4	2.6	2.13	2.12
	ID	0.34	0.17	0.72
	MIR	0.72	0.56	0.53
	other SINE	0.24	0.01	0.01
LINE	L1	16.22	19.75	18.94
	L2	0.57	0.43	0.41
	other LINE	0.13	0.1	0.1
LTR	ERV1	0.64	0.74	0.99
	ERVK	5.02	5.02	3.81
	ERVL	5.55	5.9	5.26
	other LTR	0.87	0.44	0.04
DNA	hAT	0.98	0.82	0.81
	TcMar	0.27	0.22	0.21
	other DNA	0.03	0.03	0.03
Other TE		0.38	0.29	0.27
Unknown TE		0.09	0.12	0.79
**Total**		**39.9**	**41.22**	**38.7**

**Figure 4.**
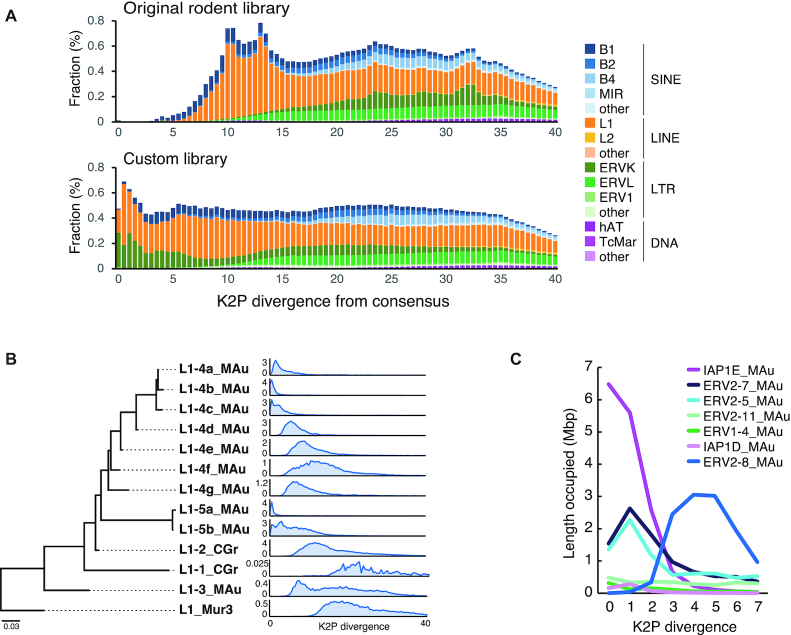
Identification of active transposable elements in the hamster genome. (**A**) Age distribution of the hamster TEs compared between the original and customized repeat libraries. The proportion of TEs is shown for 0.5 bins of Kimura 2-parameter (K2P) distance (CpG-corrected) from each consensus sequence. (**B**) A Maximum-likelihood tree of the L1 ORF2 subfamilies and their age distribution (0.5 bins of K2P distance). The TE amount is the length occupied in Mbp. (**C**) Amount of recently-active LTR retrotransposons compared among representative IAP and other ERV2 families.

The custom library substantially improved the detection ability of recently active TEs, as represented by a higher proportion of young elements, for example, those with low (<5.0) Kimura two-parameter (K2P) divergence from the consensus (Figure 4A). Hamster-specific subfamilies of B1 SINE were recently active, and 36 000 copies of young full-length B1s are present in the assembly. We identified three groups of the LINE-1 (L1) family, two of which were recently highly active (L1–4_MAu and L1–5_MAu), and the genome harbors at least 1000 young full-length copies (Figure 4B). The most active LTR retrotransposons belong to the ERV2/ERVK superfamily, including IAP (for ERV classification, see (79,80)). We identified 20 families of ERV2 that contain an internal portion between LTRs, and 11 of them possess a clear reverse transcriptase domain. There are over 13 000 LTR sequences and 1600 internal portions of the recently active elements in the genome. Among them, we found three recently expanded families: IAP1E_MAu, ERV2–5_MAu and ERV2–7_MAu, which accounted for 29.9%, 14.7% and 26.8% of the very young (<2.0 K2P divergence) LTR retrotransposons, respectively (Figure 4C). It is likely that not only IAP but also other ERV2/ERVK elements are currently active in the hamster genome. Together, these results show that our effort to re-sequence the golden hamster genome significantly improved annotations, especially for recently active TEs, which are potential piRNA targets.

### Characterization of piRNAs loaded onto PIWIL1 or PIWIL3

**Figure 5.**
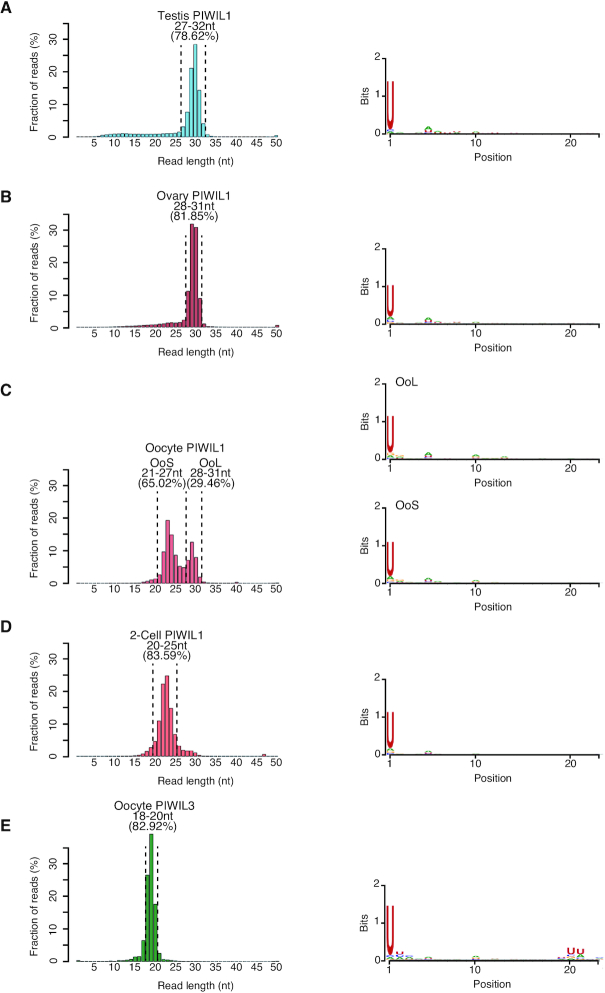
Characterization of PIWI-associated piRNAs in hamster male and female gonads. Size distribution and nucleotide bias in PIWIL1-associated piRNAs in hamster (**A**) testis, (**B**) ovary, (**C**) MII oocyte, (**D**) 2-cell embryo and (**E**) PIWIL3-associated piRNAs in hamster MII oocytes. Left panel: size distribution; peaks of size distribution are shown at 29–30 nt in testis and ovary PIWIL1-piRNAs, 29–30 nt and 23–24 nt in MII oocyte PIWIL1-piRNAs, 22–23 nt in 2-cell embryo PIWIL1-piRNAs and 19 nt in MII oocyte PIWIL3-piRNAs, respectively. The right panel shows nucleotide bias. All piRNA populations have a strong uridine (U) bias at their 5′-end.

To characterize piRNAs loaded onto PIWIL1 or PIWIL3 in oocytes, we performed small RNA sequencing using piRNA samples immunopurified with an anti-PIWIL1 or an anti-PIWIL3 antibody from oocytes. For PIWIL1-associated piRNAs, we also sequenced piRNA samples immunopurified with an anti-PIWIL1 antibody from testes. Replicates were highly correlated with each other (*R*^2^ > 0.9) (data not shown); therefore, we merged the reads. First, we performed a length distribution analysis of the obtained reads (Figure 5A–E). We observed peak signals at the size range of 29–30 nt in the testes and ovaries, 29 nt and 23 nt in MII oocytes, and 23 nt in 2-cell embryos of PIWIL1-associated piRNAs and 19 nt in MII oocytes of PIWIL3-associated piRNAs, confirming that the length of PIWIL1- and PIWIL3-associated piRNAs sequenced is consistent with that observed on the gels. Then we divided into two groups using the length of PIWIL1-associated piRNAs in MII oocyte; Oocyte long piRNAs (L1 OoL-piRNAs) and Oocyte short piRNAs (L1 OoS-piRNAs) for further analysis. Sequencing also revealed that the piRNA populations in all samples showed a strong 1U bias, a conserved characteristic for piRNAs (Figure 5A–E).

**Figure 6.**
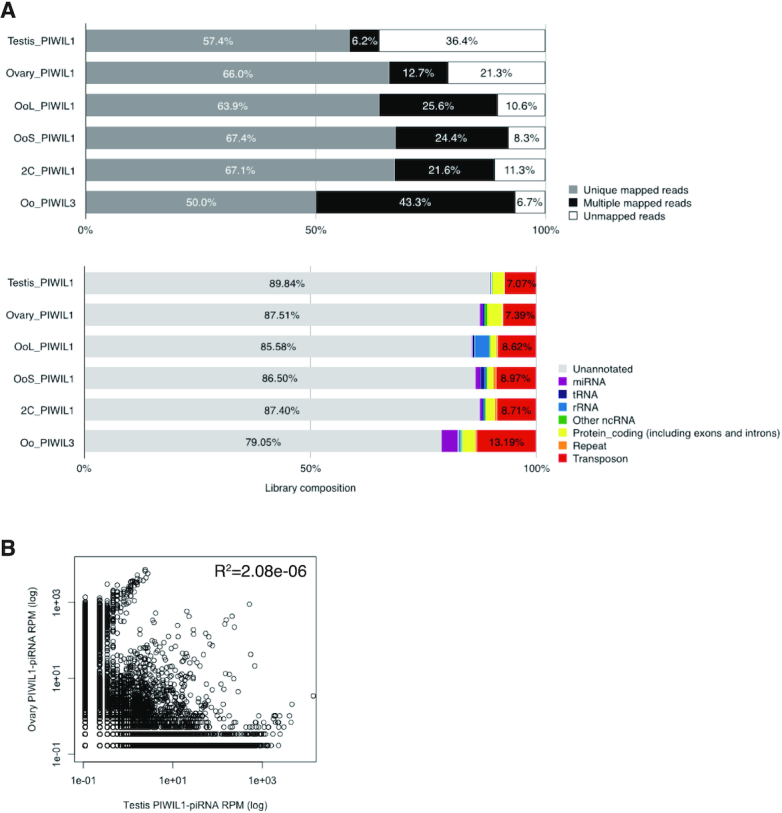
Characterization of genome mapped PIWI-associated piRNA reads in hamster male and female gonads. (**A**) Genome mapping ratio and annotation of PIWIL1- and PIWIL3-associated piRNA reads in hamster testis, ovary, MII oocyte and 2-cell embryo. The upper panel exhibits the genome mapped ratio of each PIWI-associated piRNA reads: (gray) unique mapped reads; (black) multiple mapped reads; (white) unmapped reads. Lower panel exhibits the results of annotation of genome mapped reads: (red) transposon; (orange) repeat; (yellow) protein-coding gene, including exon and intron; (purple) miRNA; (blue) tRNA; (skyblue) rRNA; (green) other ncRNA, including snoRNA and snRNA. (**B**) Comparison between testis and ovary PIWIL1-associated piRNAs. Pearson correlation coefficient was calculated using R. Each dot exhibits the specific piRNA reads.

We then mapped piRNAs to the new hamster genome (hamster.sequel.draft-20200302.arrow.fasta). Among the PIWI-associated piRNA reads, 50.0–67.4% of the reads were uniquely mapped to the genome and 6.2–43.3% were mapped multiple times (Figure 6A, upper panel). Then, we annotated each piRNA read to analyze the genomic region from which the piRNA reads were generated. Approximately 79.05–89.84% of the reads mapped to unannotated intergenic regions, and only 7.07–13.19% originated from TEs (Figure 6A, lower panel). This profile is similar to that of pachytene piRNAs associated with mouse MIWI, in which ∼70% is derived from intergenic regions and ∼24% from TEs (33).

The characteristics of PIWIL1-piRNAs were virtually indistinguishable between the testis and ovary. To determine whether this was due to the common piRNA sequences, we calculated reads per million mapped reads (RPM) for each piRNA sequence and compared the correlation between testis and ovary samples. The RPM of piRNAs greatly varied between the testis and ovary samples (*R*^2^ = 2.08e–06). In addition, when we checked the top 10 piRNA sequences with the most abundant read counts, none of the sequences were common between the testis and ovary (data not shown). These results show that testis and ovary PIWIL1-piRNAs possess distinct sequences (Figure 6B).

We found that most piRNAs in hamster female gonads were derived from LTR retrotransposons, including endogenous retroviruses (ERVs), compared to PIWIL1–piRNAs in the hamster testis, which corresponded to both LINEs and LTR retrotransposons (Supplementary Figure S5). This observation is consistent with the fact that the main targets of the piRNA pathway in mouse testes are L1 and IAP elements. In the mouse genome, L1 is the most active TE family, as represented by an increasing accumulation of their young copies (Supplementary Figure S4B). There are also young LTR retrotransposons in mice, among which IAP and MERVL (ERV3/ERVL) families are highly active, while ERV2/ERVK families, except IAPs, showed much lower proportions among the young elements (Supplementary Figure S4C). Interestingly, most LTR retrotransposons from which piRNAs in hamster female gonads were derived were evolutionally younger judged by the K2P divergence (Figure 4C). This suggests that hamster oocyte piRNAs were generated from the higher activity of recent transposition. In contrast, testis PIWIL1-piRNAs target both LINEs and LTR retrotransposons. Together with the diversity in PIWI-associated piRNA sequences of the oocyte and testis (Figure 6B), this supports the notion that PIWI-piRNA target TEs are different for male and female gonads.

### Relationships among PIWIL1- and PIWIL3-bound piRNAs in hamster oocytes

**Figure 7.**
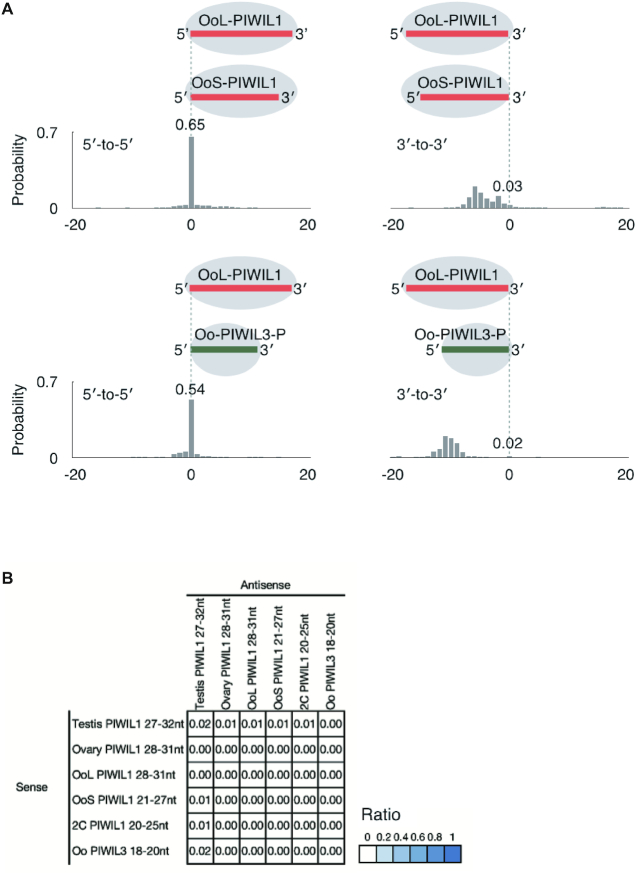
PIWIL1- and PIWIL3-associated piRNA populations are likely to share the same 5′ cleaved piRNA precursors. (**A**) Probability of distances between the 5′- or 3′-ends of OoL PIWIL1 (longer)- and OoS PIWIL1- or PIWIL3 (shorter)- piRNAs (Oo PIWIL3). The numbers indicate the total frequency of the 5′- or 3′-ends of shorter piRNAs residing before, after, or coinciding with the 5′- or 3′-ends of the longer piRNAs. This indicates that piRNA populations are likely to share the same 5′ cleaved piRNA precursors, but the 3′-end formation of these populations may be determined by the footprint of piRNA-binding PIWI proteins probably because of either structure. (**B**) A heat map showing the probability that the first base of the PIWI-associated piRNA reads mapped to the antisense strand overlap the 10th base of the reads, which mapped to the sense strand. This result shows that they do not have any ping-pong signals with each other.

Figures 2 and 5 show that piRNAs loaded onto PIWIL1 in MII oocytes consist of two distinct populations, short piRNAs (L1 OoS-piRNAs) and long piRNAs (L1 OoL-piRNAs). Shorter piRNAs (18–20 nt) were loaded onto PIWIL3 in MII oocytes. These findings prompted us to test whether these piRNAs are derived from the same genomic loci and whether L1 OoS-piRNAs and/or PIWIL3-associated piRNAs are processed products of L1 OoL–piRNAs by cleaving and/or trimming their 3′-ends. To this end, we asked whether the genomic positions of the 5′- or 3′-ends of the L1 OoL–piRNAs differ from those of L1 OoS-piRNAs or PIWIL3–piRNAs and calculated the probabilities of the 5′- or 3′-ends of L1 OoL-piRNAs coinciding with the 5′- or 3′-ends of L1 OoS–piRNAs or PIWIL3-piRNAs (18). In MII oocytes, L1 OoL-piRNAs were much more likely to share 5′-ends with L1 OoS–piRNAs and PIWIL3–piRNAs (Oo PIWIL3) than with 3′-ends (0.65 for 5′-ends versus 0.03 for 3′-ends and 0.54 for 5′-ends versus 0.02 for 3′-ends, respectively; Figure 7A). These results suggest that approximately half of these three piRNA populations share the same 5′ cleaved piRNA precursors, thereby sharing the same piRNA clusters as piRNA sources. Thus the size differences among these piRNAs account for their 3′-end formation of these populations that may be determined by the footprint of piRNA-binding PIWI proteins, probably because of either the structures of partner PIWI proteins (in particular, the distance between MID-PIWI and PAZ domains) or conformational changes in the partner proteins (see Discussion).

The finding that piRNA populations in oocytes share the same 5′ cleaved piRNA precursors suggests little ping-pong amplification among them. We calculated the ping-pong signature of each piRNA and found that they had little or no ping-pong signatures (Figure 7B). This result indicates that most of the piRNAs expressed in oocytes are not involved in the ping-pong cycle. Recently, a novel class of small RNAs with 21–23 nt, termed short PIWI-interacting RNAs (spiRNAs), was identified in mouse oocytes (41). They are produced by the ping-pong cycle driven by the MILI slicer activity, and thus, small piRNAs found in hamster oocytes are distinct from spiRNAs.

### piRNA clusters in the oocytes are distinct from those observed in the testis

**Figure 8.**
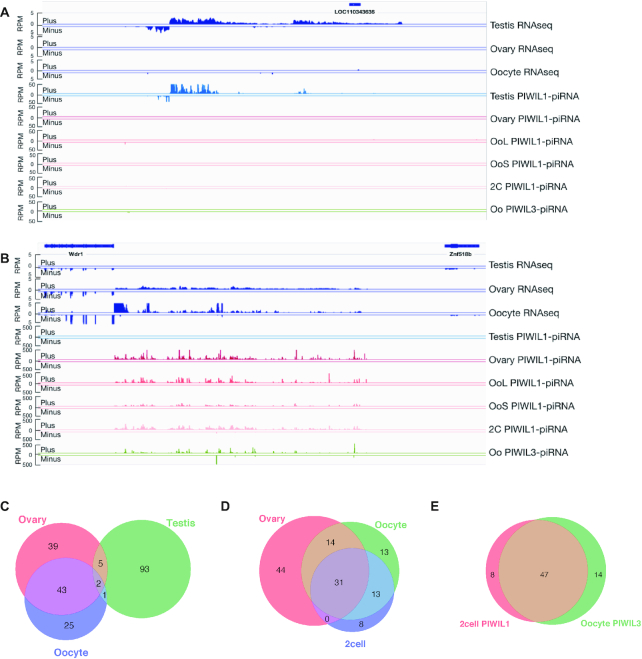
piRNA clusters in hamster male and female gonads. (**A**) Example of male-specific piRNA cluster in hamster. Distributions of uniquely mapped piRNAs and RNA-seq reads located in piRNA clusters are shown. (**B**) Example of female-specific piRNA cluster in hamster. Distributions of uniquely mapped piRNAs and RNA-seq reads located in piRNA clusters are shown. (**C**) The Venn diagram shows the amount of overlap among PIWIL1-piRNA clusters, which are consistent with 28∼30 nt piRNAs in hamster testis, ovary, and MII oocyte. (**D**) The Venn diagram shows the amount of overlap among PIWIL1–piRNA clusters in hamster ovary, MII oocyte and 2-cell embryo. (**E**) The Venn diagram shows the amount of overlap among PIWIL1–piRNA clusters in hamster 2-cell embryo and PIWIL3–piRNA clusters in hamster MII oocyte.

We found that more than half of PIWIL1- and PIWIL3-associated piRNAs are likely to share 5′-ends of precursor transcripts, suggesting that the primary source of piRNAs is also shared among them. To test the possibility that PIWIL1- and PIWIL3-associated piRNAs share piRNA clusters from which they are derived, we next focused on the identification of piRNA clusters in hamster testes, ovaries, MII oocytes, and 2-cell embryos. We identified piRNA clusters using proTRAC (version 2.4.3) under the following conditions as described (40) with some modifications: (i) >75% of the reads were appropriate to the length of each piRNA; (ii) >75% of the reads exhibited the 1 U or 10 A preference; (iii) >75% of reads were derived from the main strand and (iv) -pimin option with 21, 28 and 18 for oocyte PIWIL1–piRNAs and PIWIL3–piRNAs, respectively. We identified 101, 89, 74, 55 and 61 piRNA clusters in testis PIWIL1–piRNAs, ovary PIWIL1–piRNAs, MII oocyte PIWIL1–piRNAs, 2-cell PIWIL1–piRNAs and oocyte PIWIL3–piRNAs, respectively. In the testis, both unidirectional clusters, in which piRNAs map to only one strand, and bidirectional clusters, in which the polarity of piRNA production switches between plus and minus strands, were identified, although unidirectional clusters were dominant (Figure 8A and Supplementary Figure S7A). We found that the majority of the piRNA clusters identified in female gonads were unidirectional (Figure 8B and Supplementary Figure S6A).

Interestingly, approximately 90% of the piRNA clusters corresponding to PIWIL1-associated piRNAs in testis, ovary, and MII oocytes were sex-specific, and only eight piRNA clusters were identified commonly in both male and female gonads (Figure 8C). These results support the idea that transcription of the primary source of piRNA (piRNA clusters) plays a key role in the production of sex-specific piRNAs (Figures 6B and 8C). We next examined overlaps among ovaries, MII oocytes, and 2-cell embryo PIWIL1 piRNA clusters and found that they shared 31 clusters (Figure 8D). PIWIL1–piRNAs in 2-cell embryos shared approximately 85% of the source clusters with those in MII oocytes, while they shared ∼60% of the source clusters with those in the ovary, which suggests that the genomic regions where piRNAs are derived are altered during oocyte maturation. PIWIL3-associated piRNAs shared 77% of the clusters with PIWIL1-associated piRNAs in MII oocytes. These results are consistent with the findings that PIWIL1- and PIWIL3-associated piRNA populations are likely to share the same 5′ cleaved piRNA precursors (Figure 7A). Finally, we found that piRNAs loaded onto PIWIL3 in MII oocytes shared a large number of piRNA clusters with piRNAs loaded onto PIWIL1 in 2-cell embryos, suggesting that they may have common targets (Figure 8E).

Given that the transcription of piRNA clusters greatly differs in male and female gonads, we analyzed the motif sites surrounding the potential transcription start sites of the testis and ovary piRNA clusters. We first extracted sequences surrounding the transcriptional start sites of unidirectional and bidirectional piRNA clusters, predicted from small RNA-seq and RNA-seq mapping data. We used these sequences and performed motif searches using MEME v.5.1.0, which can discover motifs enriched in the given dataset. The results of the MEME analysis show that the *A-Myb* binding site is significantly represented in the bidirectional piRNA clusters in the testis transcriptional start site surrounding regions (Supplementary Figure S6B), where it was under-represented in the ovary transcriptional start site surrounding regions (data not shown). Moreover, we calculated the expression level of *A-Myb* family homolog genes in hamster testis, ovary, and oocyte using RNA-seq data, and found that *A-Myb* was especially highly expressed in the testis (TPM = 66.33), whereas it was hardly expressed in the ovary and oocyte (TPM = 2.02 in the ovary and was not detected in MII oocytes, respectively) (Supplementary Figure S6C), consistent with previous findings that in mice, *A-Myb* is expressed only in the testis, but not in the female gonads (35). It has been previously shown that *A-Myb* regulates transcription of piRNA clusters in mice and roosters (35). These results suggest that piRNA clusters in hamster testes may also be regulated by the transcription factor *A-Myb*.

In contrast, we failed to detect clear binding consensus sequences for oocyte-expressing transcriptional factors in the upstream regions of unidirectional piRNA clusters in oocytes. Although we also identified some bidirectional clusters in oocytes (Supplementary Figure S7A), clear binding consensus sequences for oocyte-expressing transcriptional factors between regions of piRNA clusters could not be detected. This could be due to a lack of information on the exact 5′-ends of the piRNA precursors. Alternatively, the transcription of piRNA clusters in oocytes may differ from the testis piRNA clusters in their modes in which, for example, multiple transcriptional factors, but not a few master transcriptional factors such as *A-Myb*, may drive the transcription of their loci.

## DISCUSSION

In this study, we generated a new golden hamster genome, which revealed the presence of young and possibly transposition-competent TEs in the genome. This also allowed us to characterize piRNAs in golden hamster oocytes. Intriguingly, the size of PIWIL1-associated piRNAs changes during oocyte maturation. In sharp contrast, PIWIL3 binds to piRNAs only in MII oocytes, and the size of loaded piRNAs is shorter (19 nt).

### PIWI proteins and piRNAs in hamster oocytes and early embryos

The change in the size of PIWIL1-associated piRNAs during oocyte maturation may necessitate unloading of the 3′-ends of the long piRNAs from PIWIL1 to shorten the long PIWIL1-associated piRNAs to short 22–23 nt either by trimming or by direct cleavage to determine their new 3′-ends. Alternatively, PIWIL1-associated short piRNAs may not be the processed products of loaded long piRNAs, but they may be produced by a mechanism similar to that produced by long piRNAs and then loaded onto either newly produced PIWIL1 or PIWIL1, which has unloaded piRNAs. Because it is thought that the size of small guide RNAs is determined by the footprint of small RNA-binding Argonaute/PIWI proteins (28), the change in the size of PIWIL1-associated piRNAs implies a change in the structure of the protein that accommodates short piRNAs. The question is how the change in the structure of PIWIL1 is induced to either unload long piRNAs or reload short piRNAs or to conclude the production of short piRNAs, but not long piRNAs, from intermediates, of which the 5′-ends are likely anchored within the MID-PIWI domain of PIWIL1. It is known that the release of the 3′-end of the guide small RNA from the PAZ domain of some bacterial Argonaute proteins occurs during target recognition, which is accompanied by conformational changes in the PAZ domain (81,82). A recent study has also shown that disengagement of the small RNA 3′-end from the PAZ domain occurs during the mammalian Argonaute activity cycle (83). Thus, it is tempting to speculate that conformational changes in PIWIL1, either upon target recognition of long piRNA-loaded PIWIL1 or by hitherto unknown mechanisms, may occur to conclude the production of short piRNAs. In other words, PIWIL1 could switch between states of structural rearrangements to produce two types of piRNAs. In this context, it is interesting that short piRNAs are loaded onto PIWIL3 when the protein is heavily phosphorylated. Indeed, we demonstrated that phosphorylation is required for PIWIL3 to associate with piRNAs. It is known that the loading of small guide RNAs onto Argonaute proteins appears to be affected by phosphorylation, although the underlying mechanisms are poorly understood (84). Phosphorylation could induce changes in the structure of PIWIL3 so that the protein is loaded with processed intermediates of piRNA precursors and the footprint of the protein determines the size of loaded piRNAs. Alternatively, but not mutually exclusive, PIWIL3 may need to be phosphorylated to stably hold piRNAs. Our findings suggest that hamster PIWI proteins in the oocyte can alternate between states (allostery). It will be interesting to model the structural changes in the PIWI protein using published data on structures of PIWI proteins and other Argonaute proteins.

We found that PIWIL3 binds piRNAs only in MII oocytes but appears to be free from piRNAs at other stages of oocyte maturation. There are precedents for piRNA-independent functions of PIWI proteins. Mouse PIWIL1 (MIWI) was found to bind spermatogenic mRNAs directly, without using piRNAs as guides, to form mRNP complexes that stabilize mRNAs essential for spermatogenesis (85). Recent studies have also shown that human PIWIL1 (HIWI) functions as an oncoprotein via piRNA-independent mechanisms (86,87). Although Argonaute/Piwi proteins tend to be destabilized when small RNAs are not loaded onto them (88), these studies suggest that PIWI proteins may be stable in some conditions in the absence of piRNAs to play a role in some cellular functions.

### piRNA-target TEs in the hamster oocyte

In mouse testes, the main target TEs in the piRNA pathway are those of LINE1 and IAP family members. Two distinct piRNA populations are present in mouse testes: pre-pachytene piRNAs are enriched in TE-derived sequences (approximately 80% of the total) (32) and pachytene piRNAs have a higher proportion of unannotated sequences, with diminished contribution from TE-derived sequences (∼25%) (10,11). These piRNAs guide PIWI proteins to target LINE1 and IAP elements and repress them either by cleaving their transcripts in the cytoplasm or by modifying their chromatin loci in the nucleus (4,5). Recent studies also support a model in which TE-derived piRNAs serve as a guide to selectively target young L1 families for *de novo* DNA methylation (89) or H3K9me3 modification (90) in fetal testes. However, the Slicer activity of both MIWI and MILI is still required to maintain TE silencing in the mouse testis after birth, suggesting that continuous cleavage of TE transcripts by the Slicer activity is essential for repressing TEs in mouse testes (33,34). We found that the contents of PIWIL1- and PIWIL3-associated piRNAs in hamster oocytes are similar to those observed in mouse pachytene piRNAs. However, the major target TEs in the piRNA pathway in hamster oocytes appear as endogenous retroviruses (ERVs), including ERV2 families. Indeed, our results are concordant with the fact that 41.5% of recently active LTR retrotransposons are accounted for by ERV2 families such as ERV2–7_MAu and ERV2–5_MAu. The differences in piRNA targets between testes and oocytes suggest the possibility that the activity of IAP and these ERV2 elements might be distinctively controlled and their young copies in the genome might have jumped in different types of germline cells. Recent studies have shown that ERVs, which constitute a large portion (8∼10%) of mammalian genomes (91), have a significant impact on shaping transcriptomes and DNA methylation patterns (methylomes) in mammalian oocytes in a species-specific manner (92). These oocyte transcriptomes and methylomes in mammals are defined by a balance between activation and repression of ERVs. Thus, the piRNA pathway is likely to contribute to the formation of species-specific transcriptomes and methylomes in oocytes. Indeed, it was recently shown that unmethylated IAP promoters in *Miwi2*-deficient mouse testes rewire the transcriptome by driving the expression of neighboring genes (93). This also implies that spermatogonial dysfunction, observed in *PIWI-*deficient mice, may not simply be due to deregulation of TEs but due to transcriptional rewriting. It will be interesting to see if the lack of PIWIL1 or PIWIL3 leads to dysfunctions in hamster oocytes.

### piRNA clusters in hamster oocytes and early embryos

We found that nearly 80% of piRNA clusters corresponding to PIWIL1-associated piRNAs in testis, ovary, and MII oocytes of hamsters were sex-specific. This is in agreement with previous studies with total small RNA-seq of ovaries, indicating that piRNAs in human and bovine ovaries mostly share common piRNA clusters with piRNAs in testes (38,39). However, a recent study showed that only about 30% of human oocyte piRNA clusters overlapped with the human testis-piRNA clusters, proving that testes and oocytes differentially express piRNA clusters (40). Our study, together with that of human oocyte piRNAs, suggest a model in which the expression of oocyte and testis piRNA clusters have different functions with distinct targets. Transcriptional factors that regulate their expression are also distinct, though further analysis, including ATAC-seq to define transcription start sites of piRNA precursors, will be needed to identify the transcriptional factors responsible for piRNA clusters in hamster oocytes. In addition, we demonstrated that nearly half of the two populations of PIWIL1-associated piRNAs in oocytes share common clusters and that nearly half of PIWIL3-associated piRNAs in MII oocytes map to clusters from which PIWIL1-associated piRNAs are derived. These results suggest the possibility that a considerable portion of oocyte piRNA cluster transcripts are processed by a common biogenesis pathway but are loaded onto distinct PIWI proteins.

In summary, we have demonstrated that piRNAs are abundantly expressed in hamster oocytes and their size changes during oocyte maturation. Given the recent development of methods to produce genome-edited hamsters (42,44), our findings have set the stage for understanding the role that the piRNA pathway plays in mammalian oocytes. Our newly assembled golden hamster genome will also greatly promote the use of golden hamster as a model to understand human disease.

## DATA AVAILABILITY

The accession number for the reassembled genome and deep-sequencing datasets reported in this paper is PRJDB10770 in DDBJ.

## Supplementary Material

gkab059_Supplemental_Files
