# Supplementary material for: Hamster PIWI proteins bind to piRNAs with stage-specific size variations during oocyte maturation
Source: Nucleic Acids Res. 2021 Feb 15;49(5):2700–20. doi: 10.1093/nar/gkab059 (PMC7969018; doi:10.1093/nar/gkab059)
Supplement: gkab059_Supplemental_Files [file gkab059_supplemental_files.zip › 201215_ManuscriptFigure_Ishino_NAR_FigS8.pdf]

Figure S8

A: all chromosomes	
hamster	All
Mus_Musculus	All
Rattus_norvegicus	All

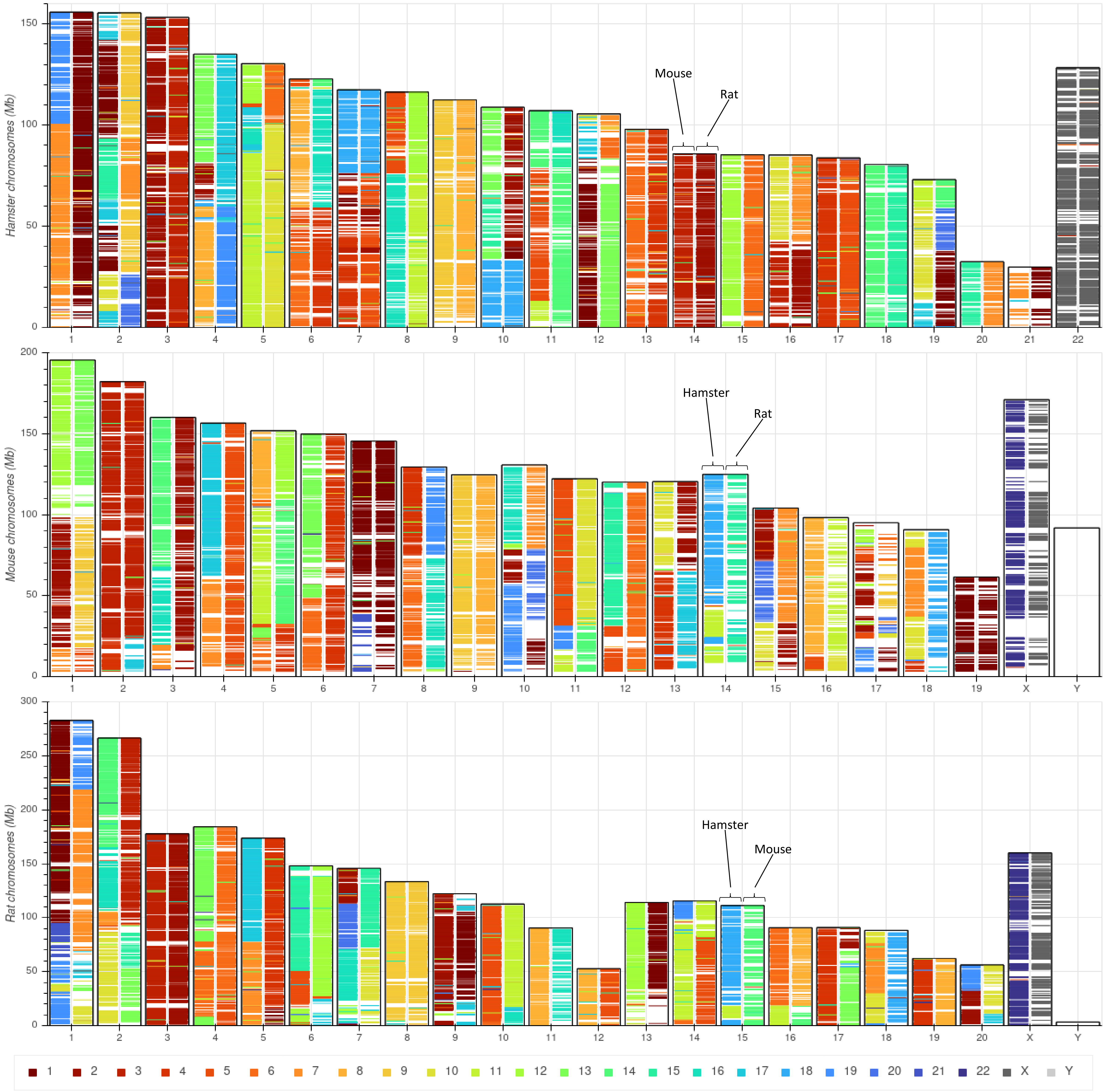

B: all chromosomes

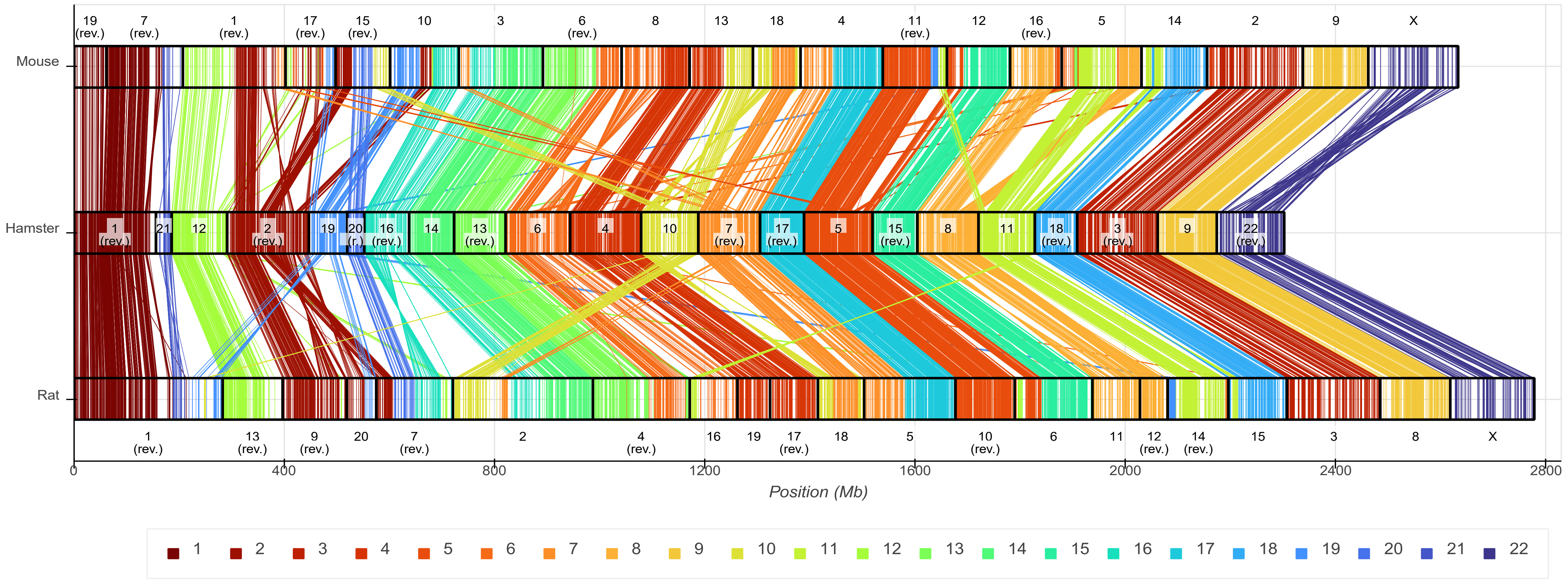

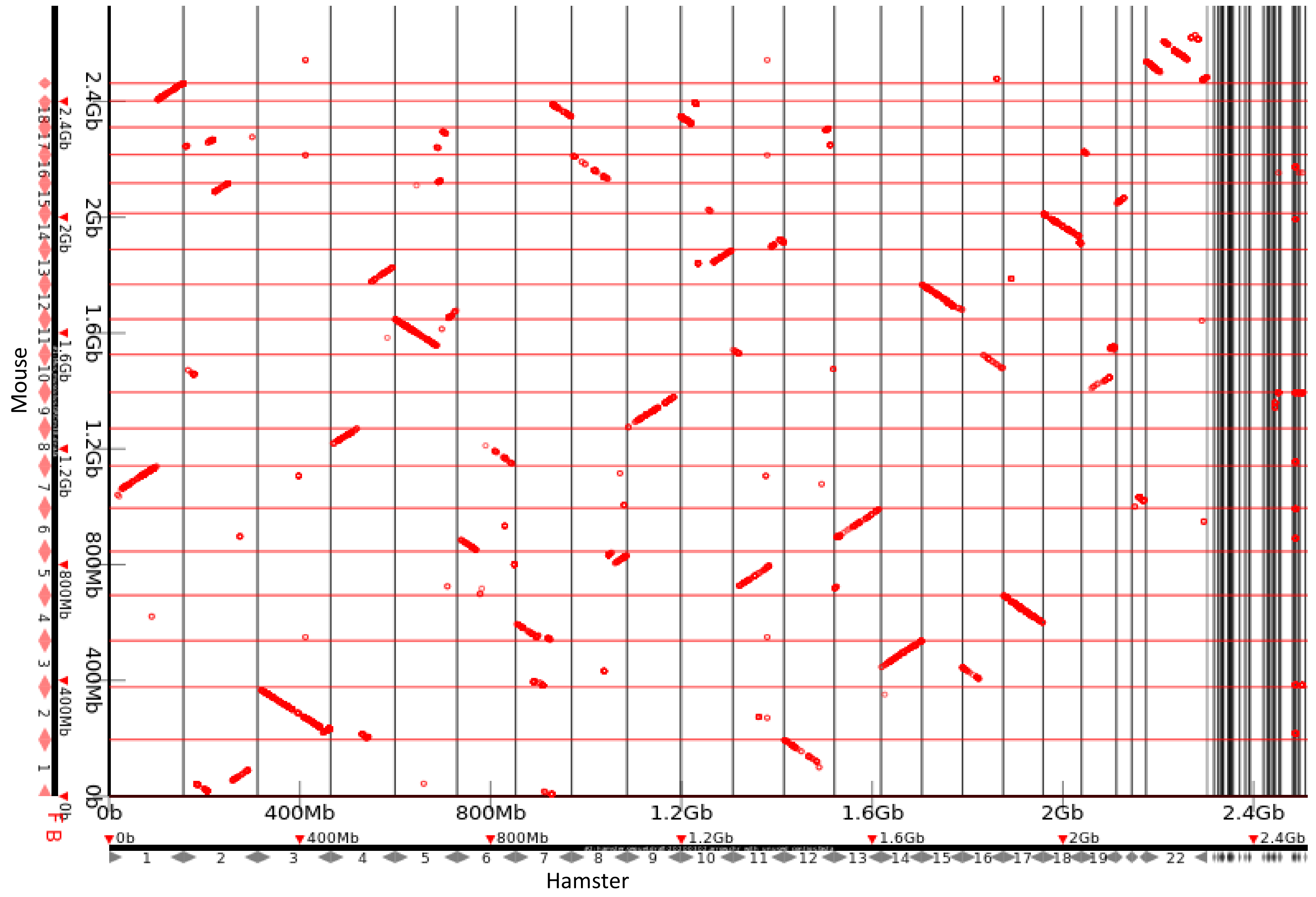

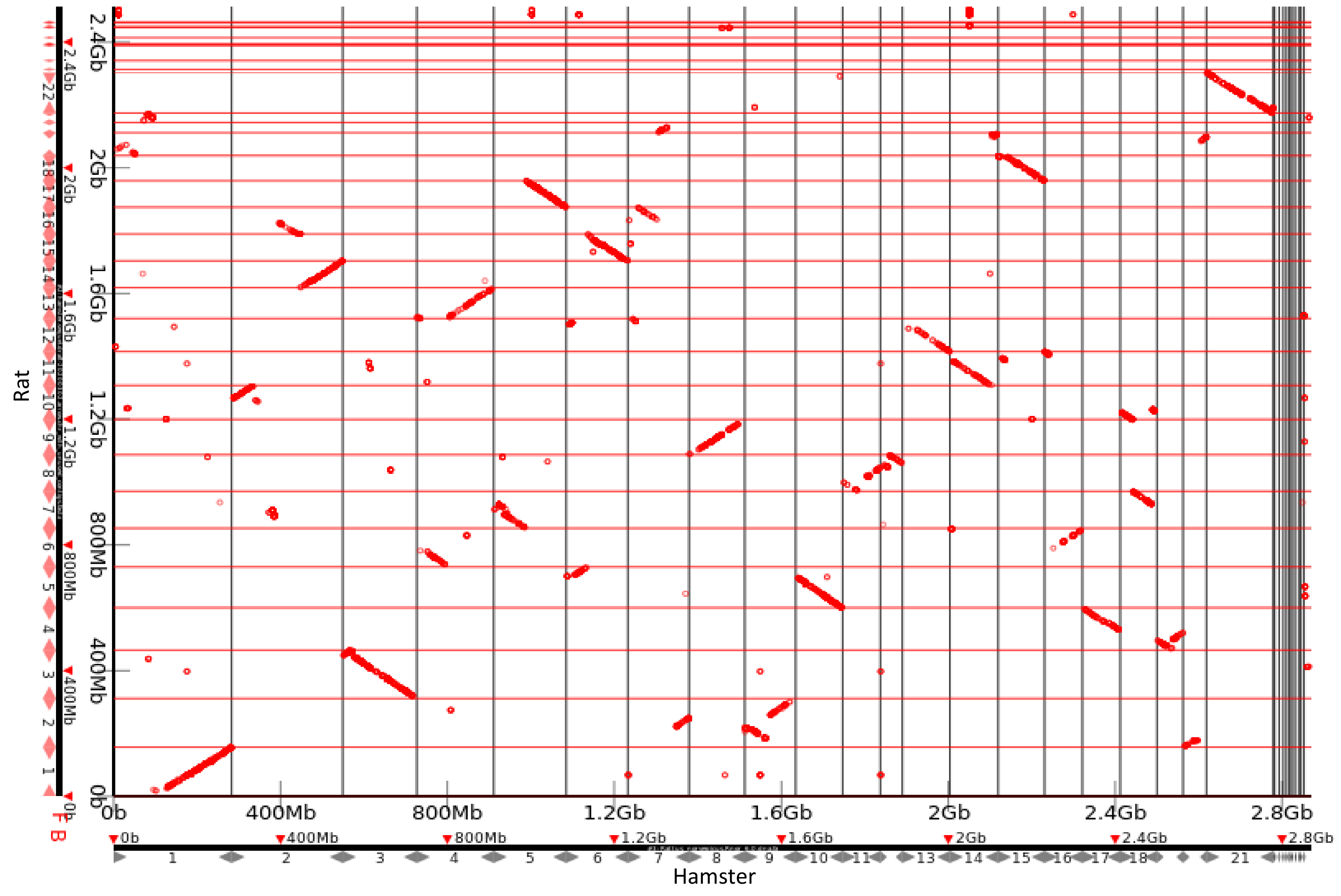

C: each chromosomes

hamster	chr1	HiC_scaffold_16
Mus_Musculus	7 & 19	
Rattus_norvegicus	1	

0bp-282,763,074bp of 282,763,074bp (100.00%)

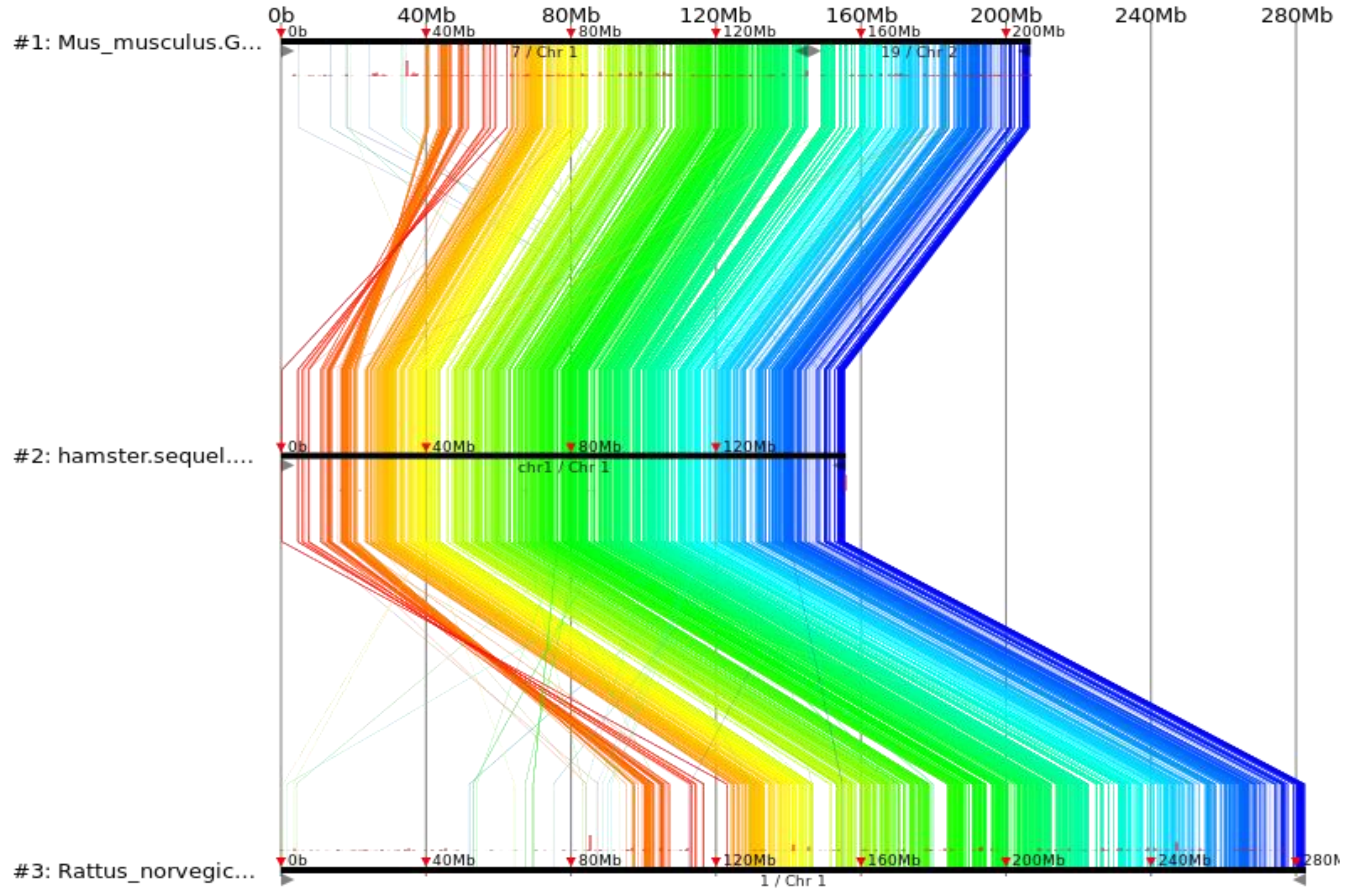

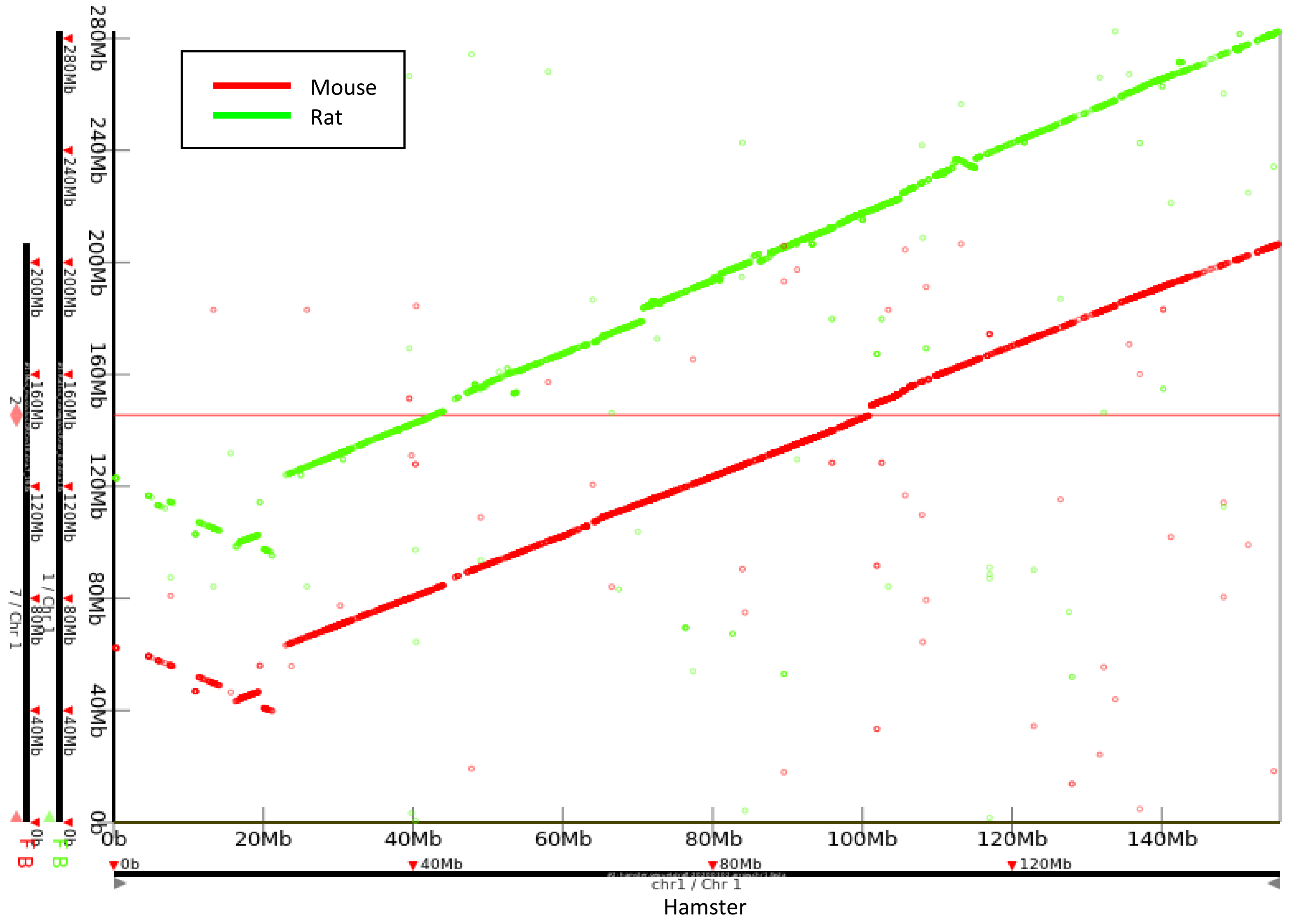

hamster	chr2	HiC_scaffold_14
Mus_Musculus	17 & 10 (rev.) & 15 & 1	
Rattus_norvegicus	20 & 7 & 9	

0bp-525,197,950bp of 525,197,950bp (100.00%)

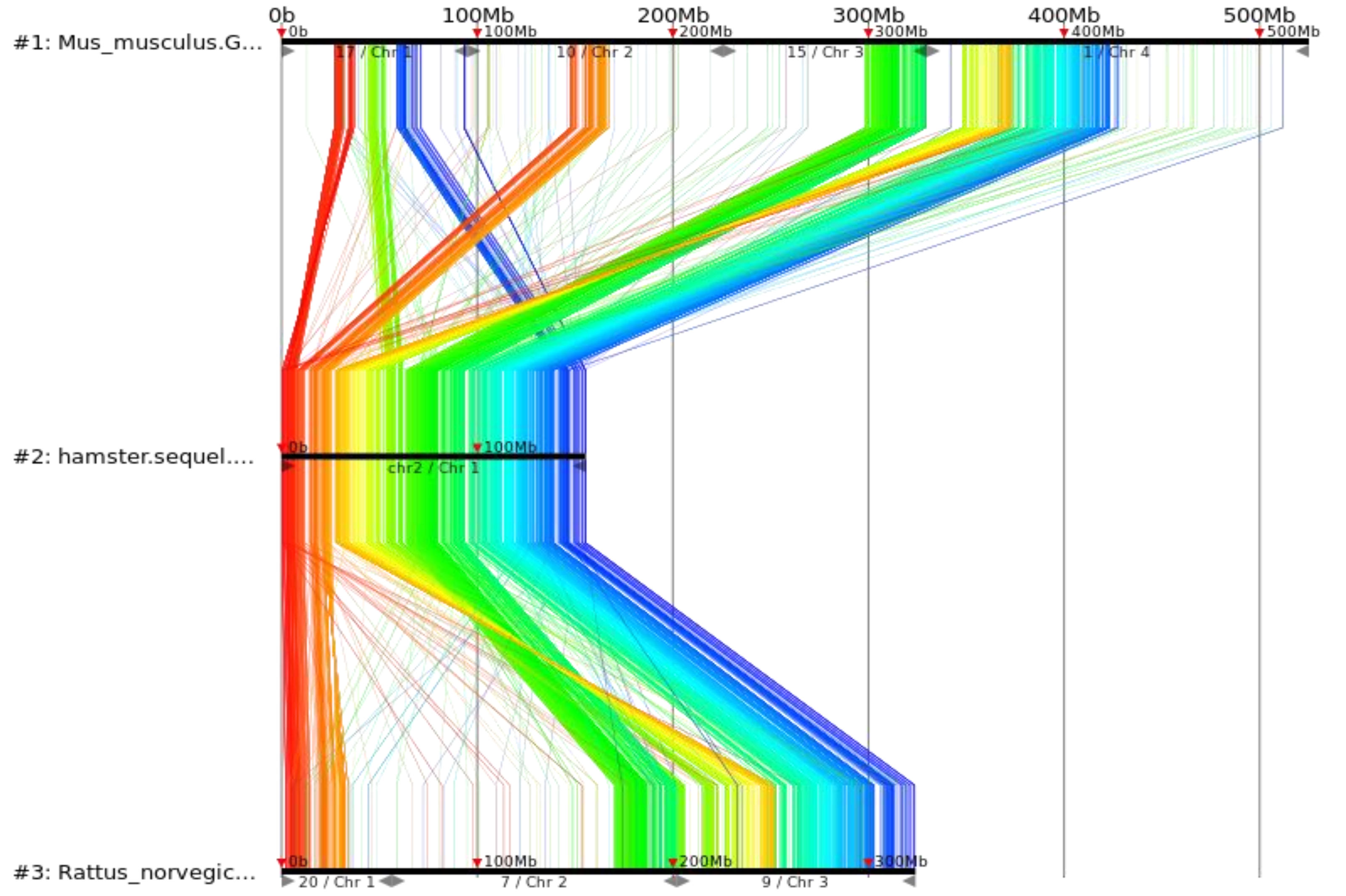

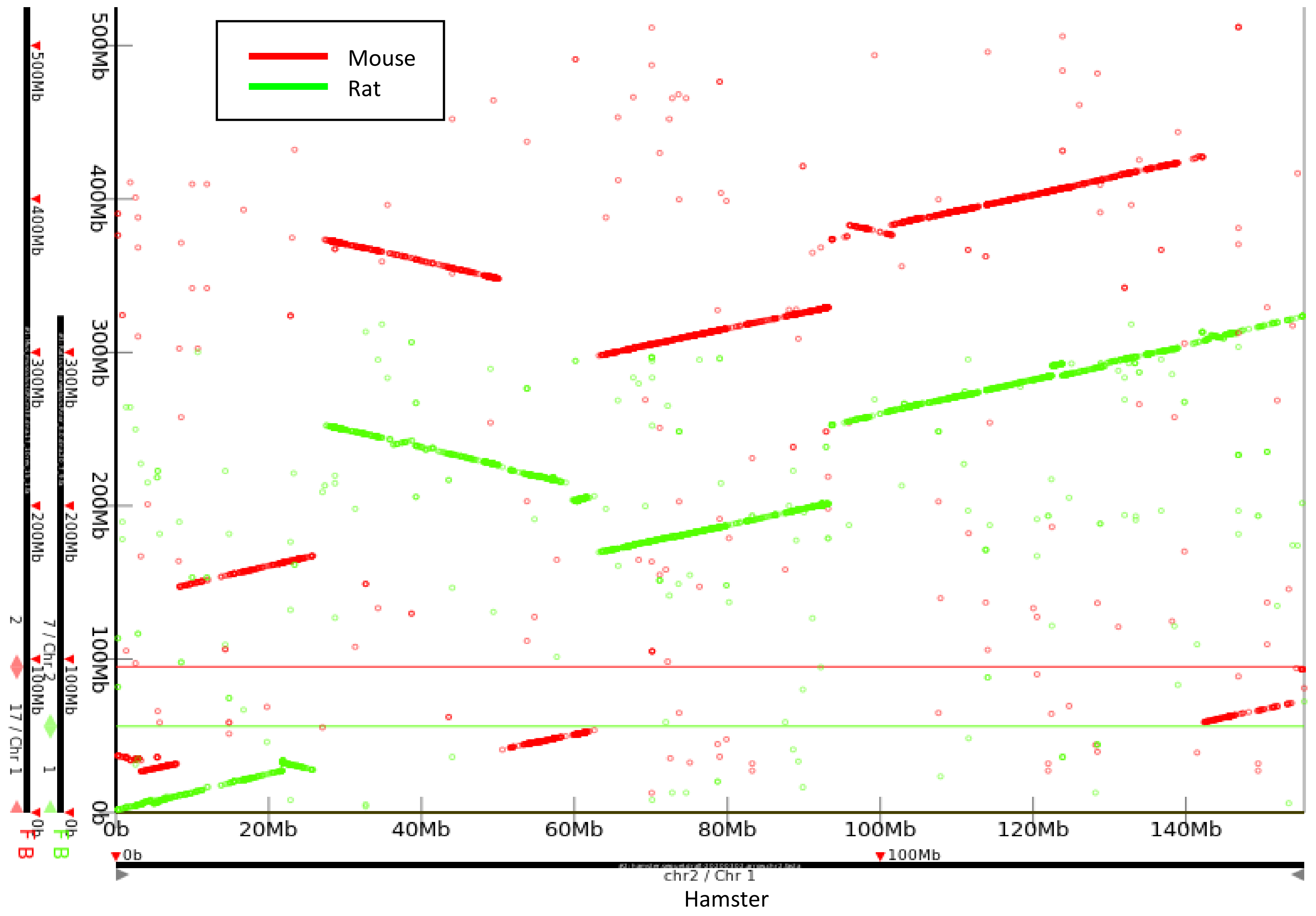

hamster	chr3	HiC_scaffold_17
Mus_Musculus	2 (rev.)	
Rattus_norvegicus	3 (rev.)	

0bp-182,113,224bp of 182,113,224bp (100.00%)

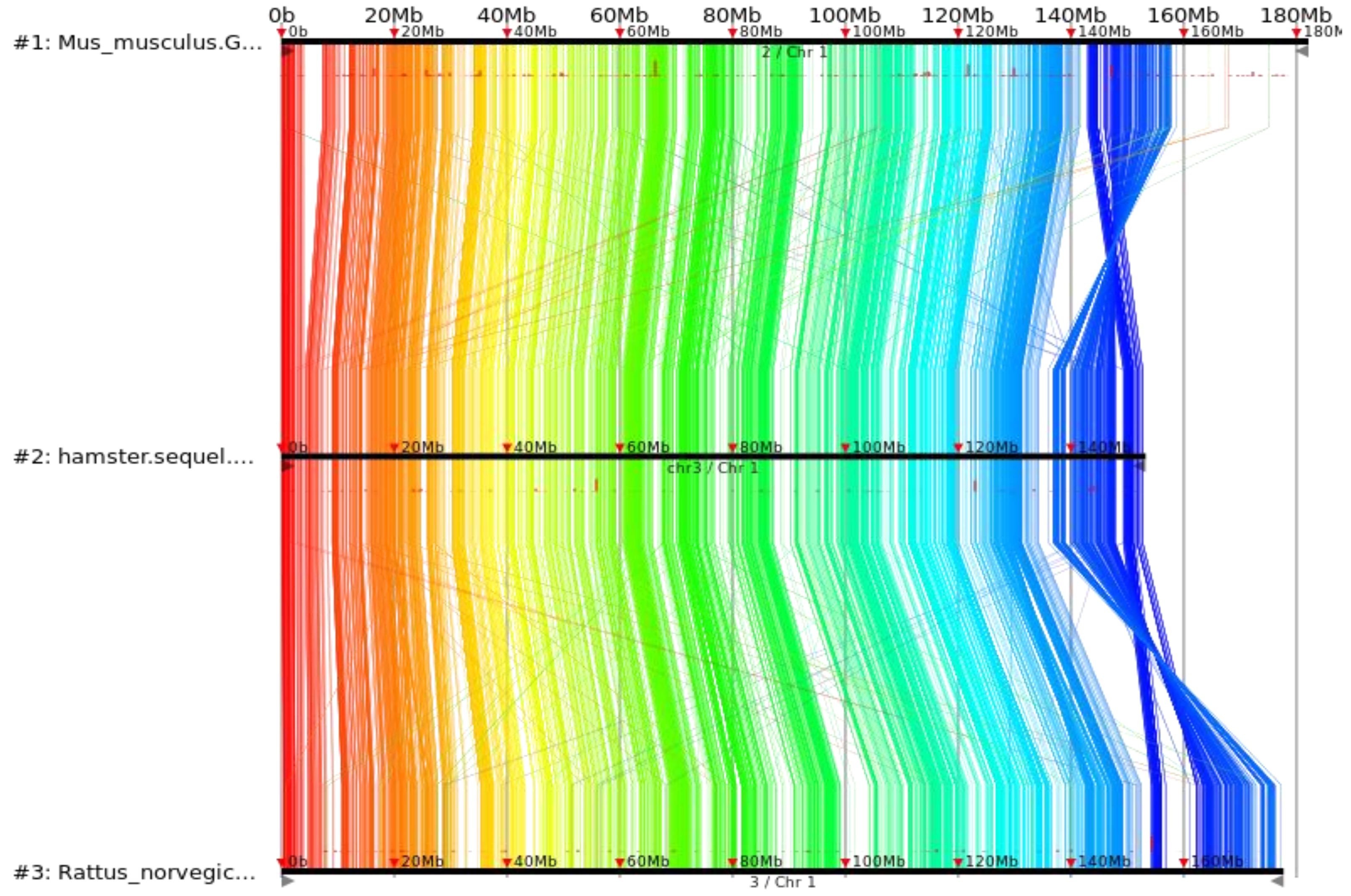

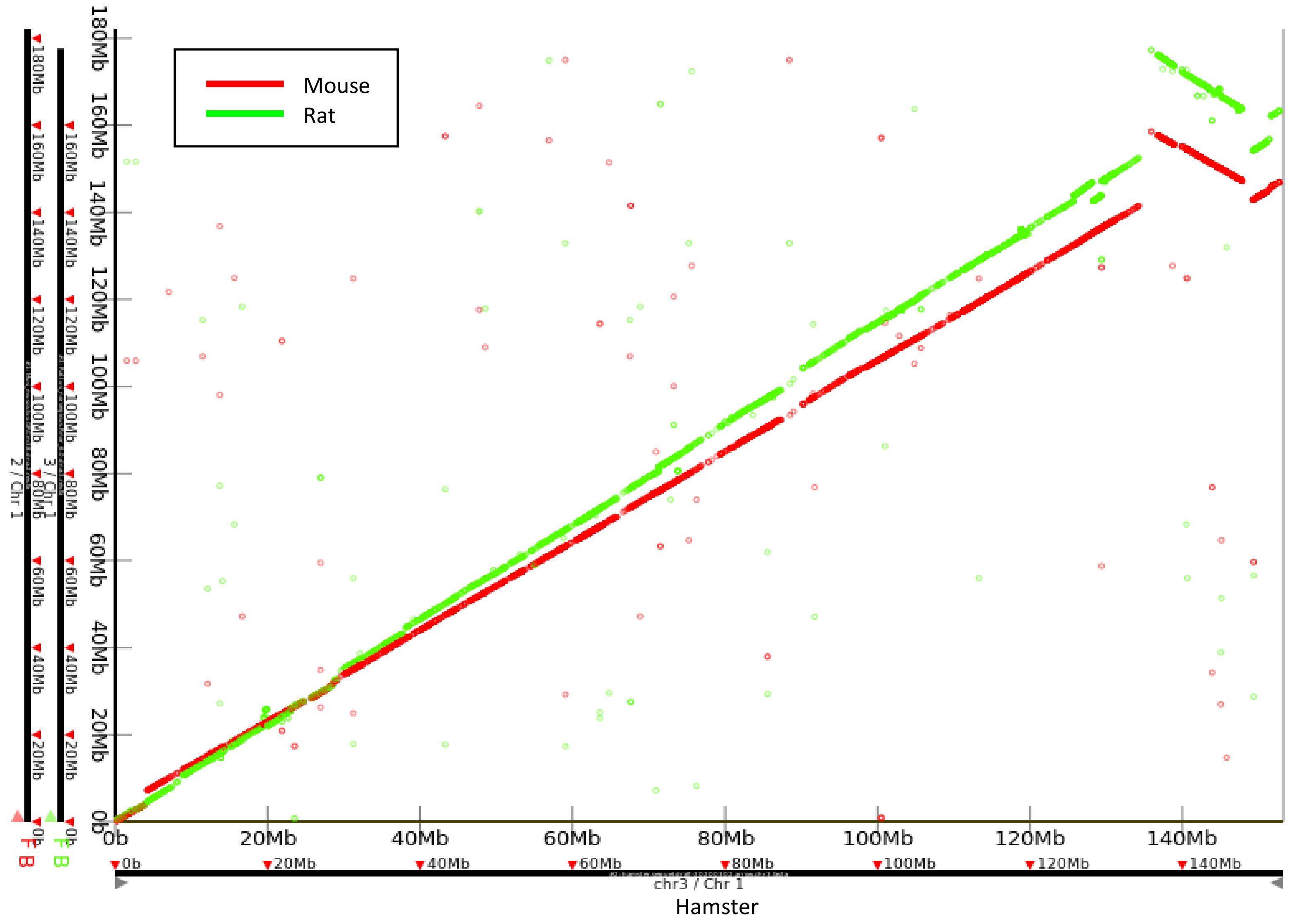

hamster	chr4	HiC_scaffold_5
Mus_Musculus	8 & 2(rev.) & 13	
Rattus_norvegicus	19 & 17 (rev.)	

0bp-431,936,096bp of 431,936,096bp (100.00%)

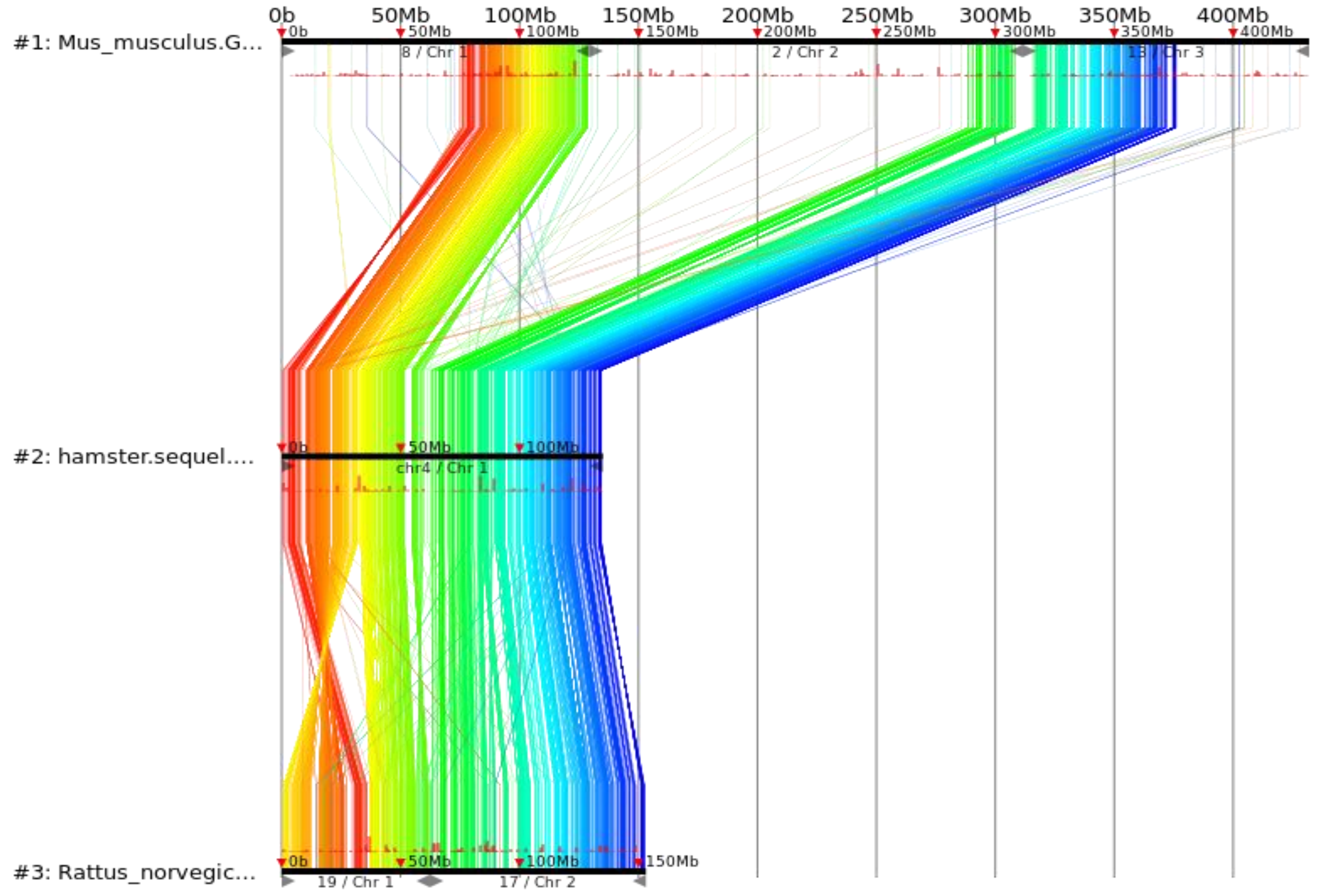

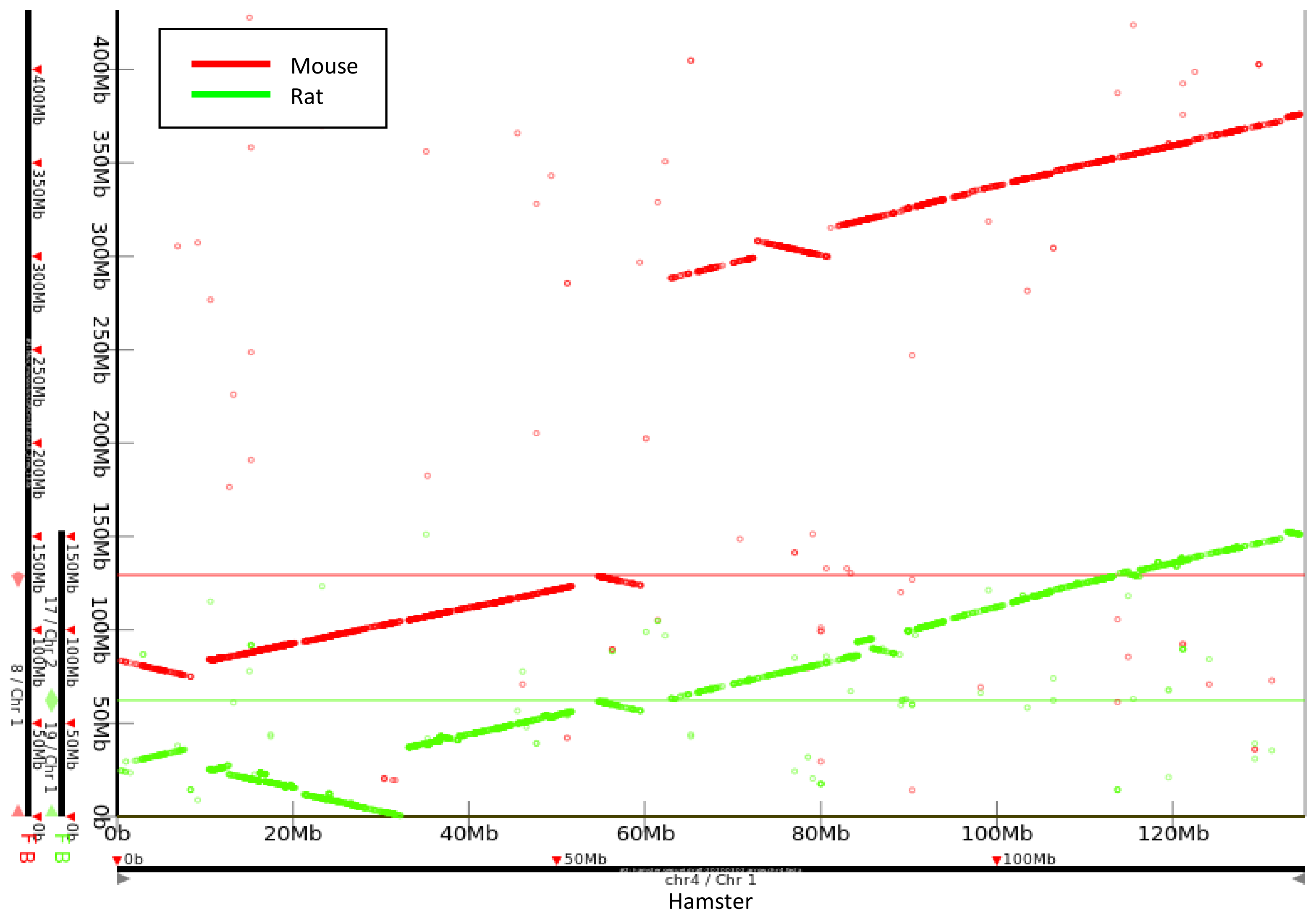

hamster	chr5	HiC_scaffold_9
Mus_Musculus	11 (rev.) & 17 & 16 & 5 (rev.) & 12	
Rattus_norvegicus	10 (rev.) & 6	

0bp-587,241,328bp of 587,241,328bp (100.00%)

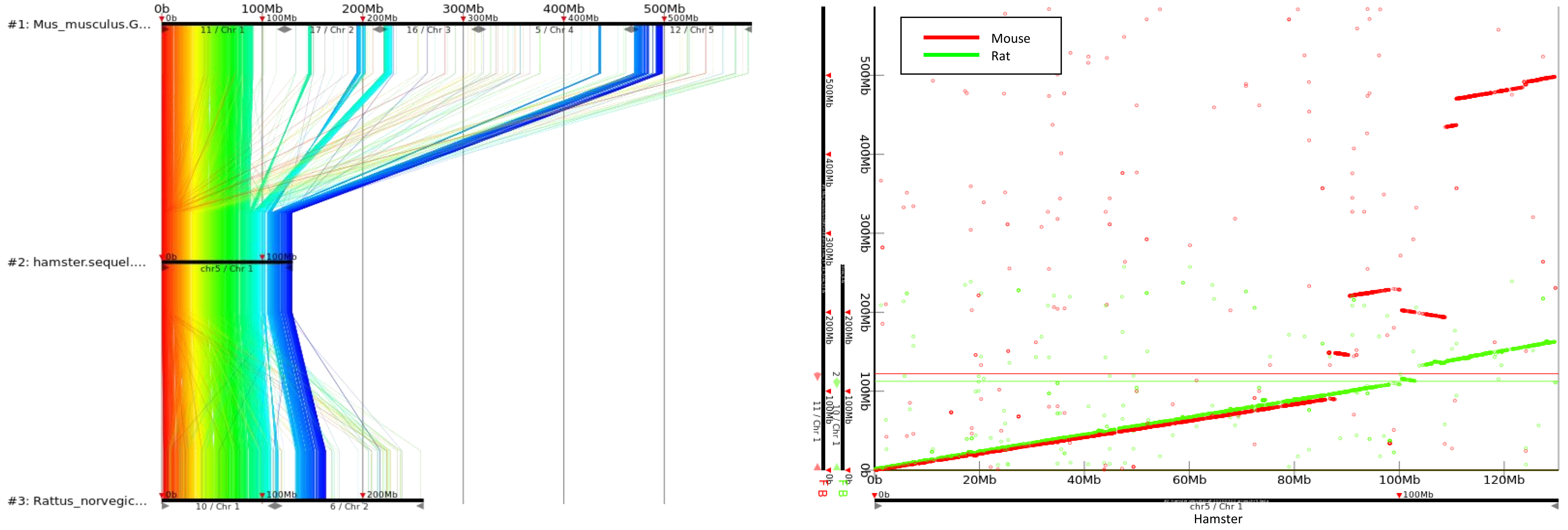

hamster	chr6	HiC_scaffold_13
Mus_Musculus	6(rev.) & 5 & 8(rev.)	
Rattus_norvegicus	4(rev.) & 16 &14(rev.)	

0bp-430,972,463bp of 430,972,463bp (100.00%)

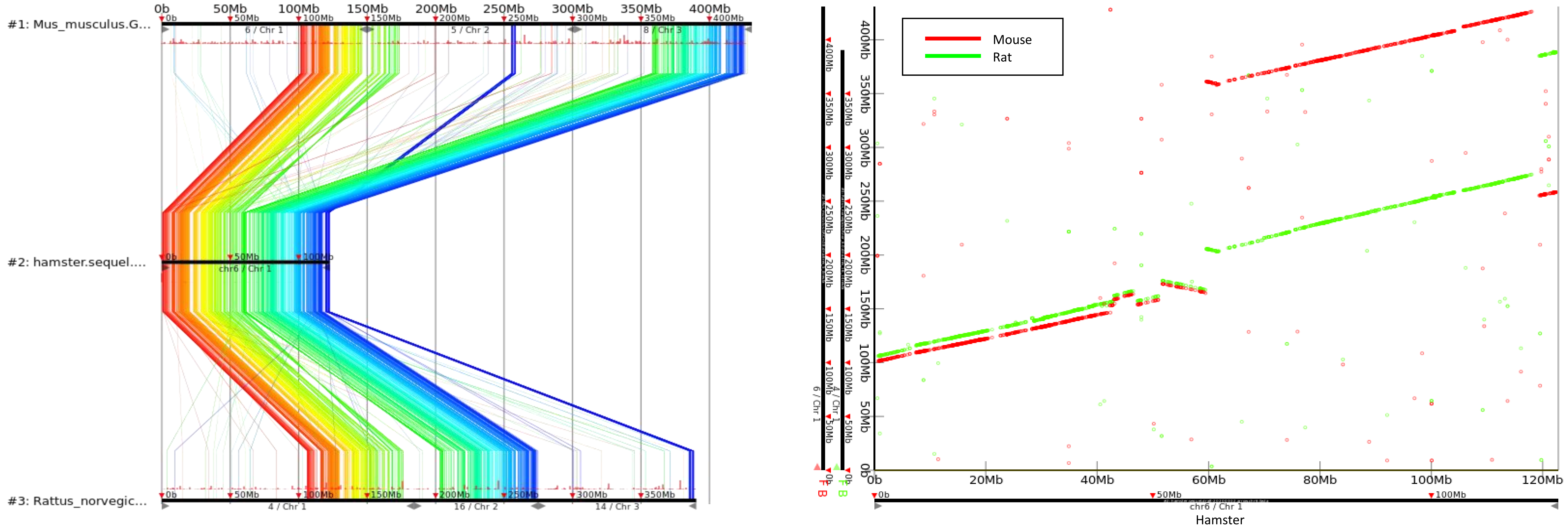

hamster	chr7	HiC_scaffold_10

Mus_Musculus 4(rev.) & 3(rev.) & 1(rev.) & 18(rev.)

Rattus_norvegicus 5(rev.) & 2 & 18(rev.)

0bp-602,722,436bp of 602,722,436bp (100.00%)

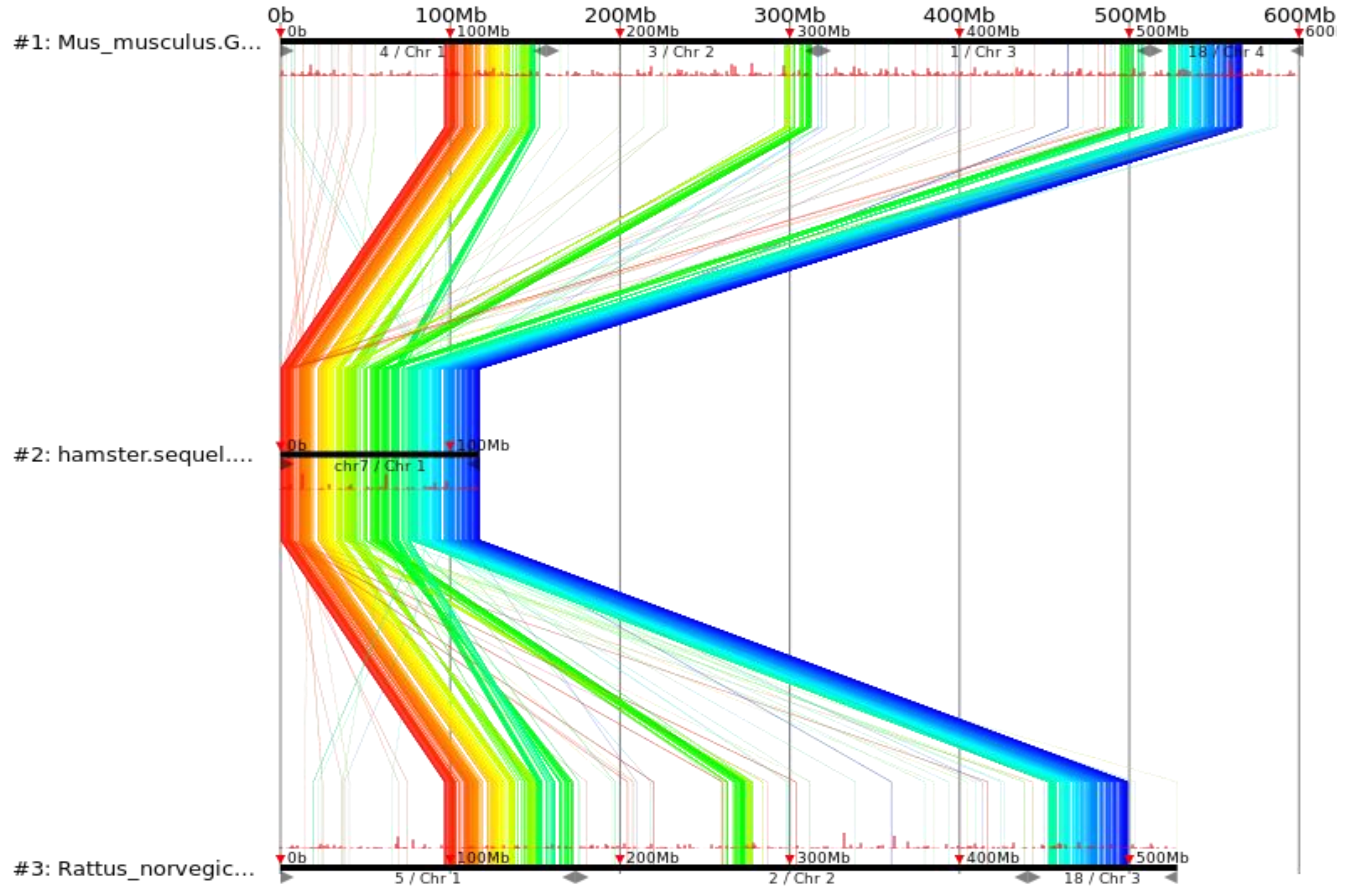

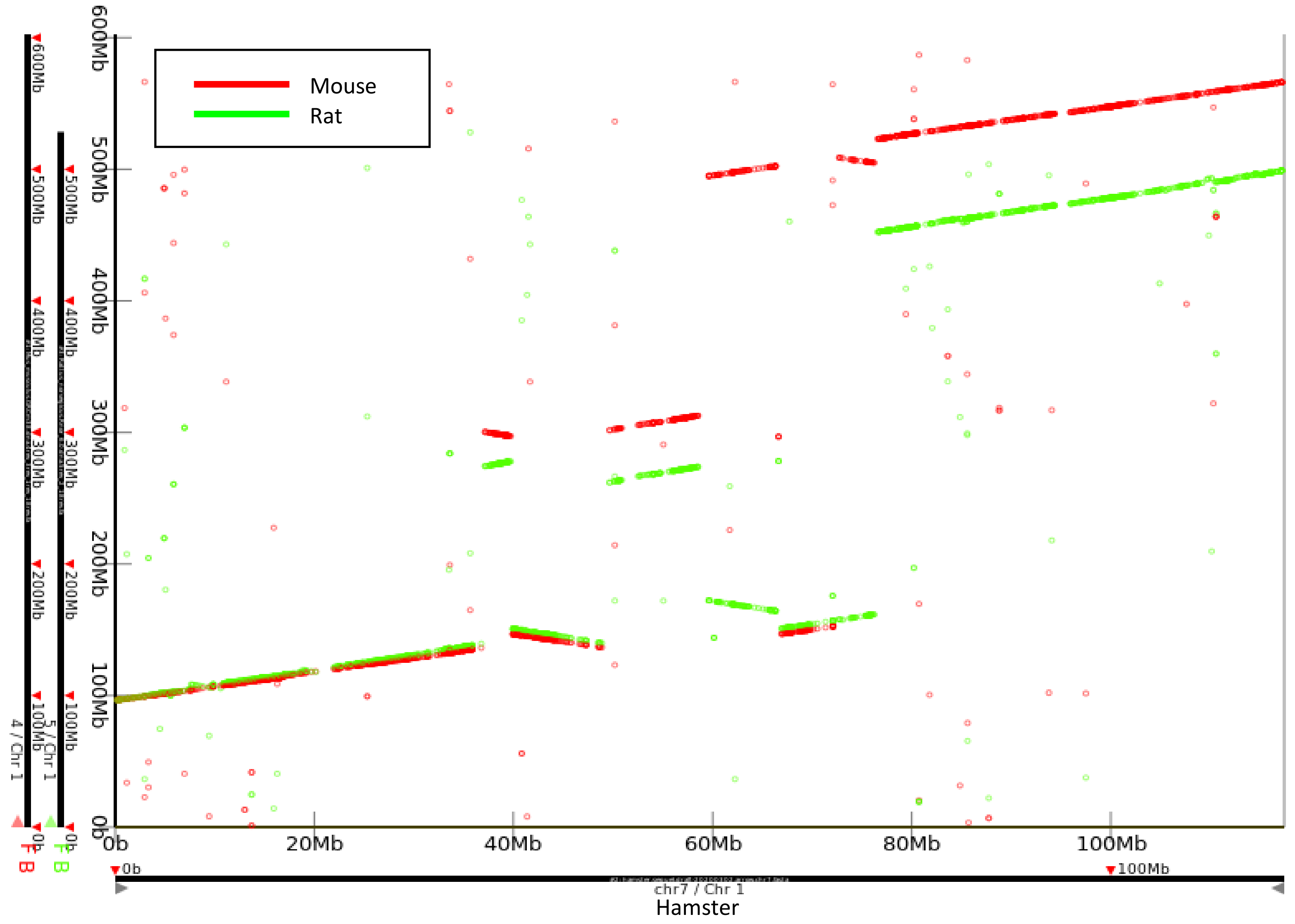

hamster	chr8	HiC_scaffold_1

Mus_Musculus 16(rev.) & 5

Rattus_norvegicus 11 & 12(rev.)

0bp-250,042,462bp of 250,042,462bp (100.00%)

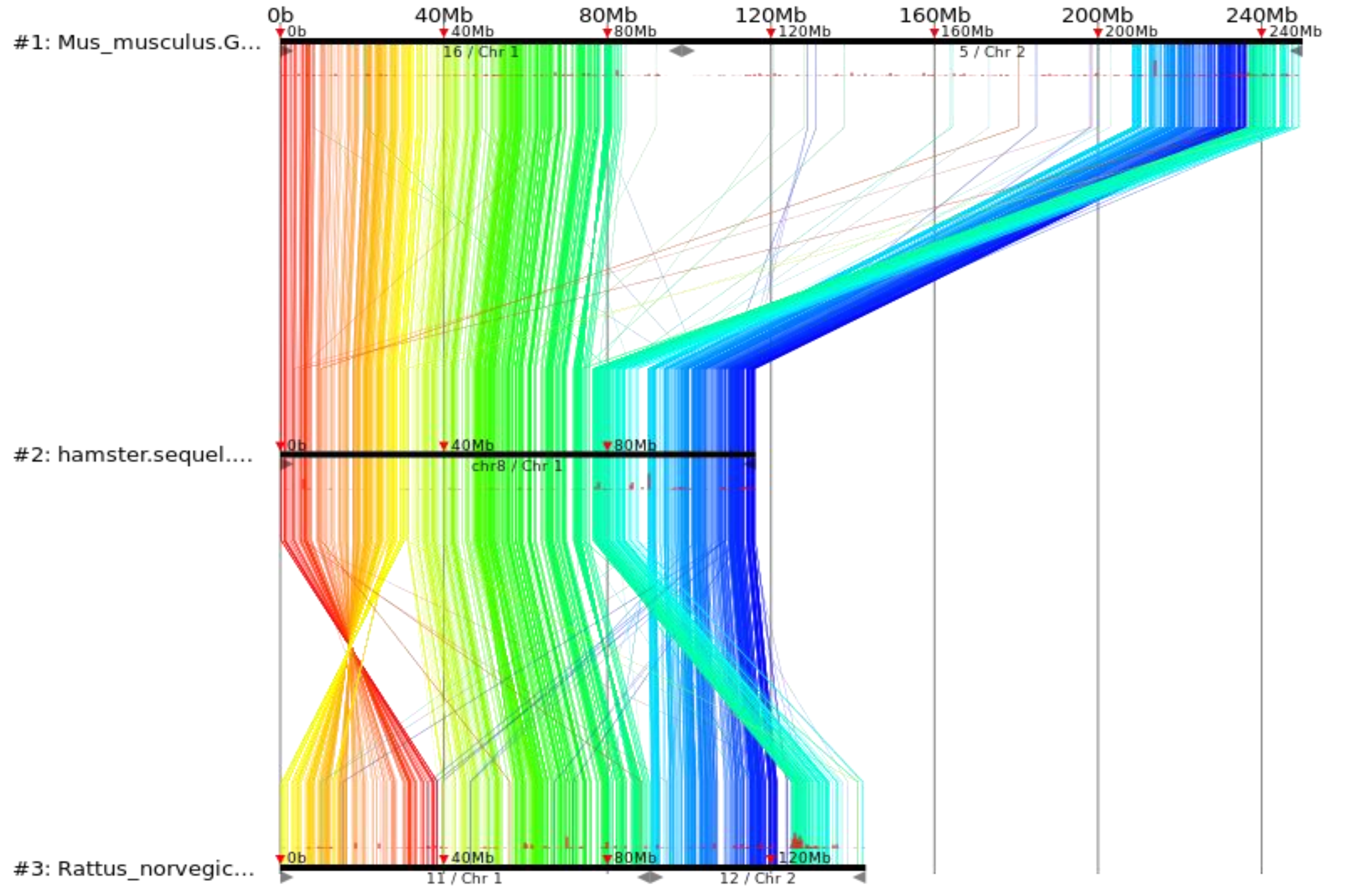

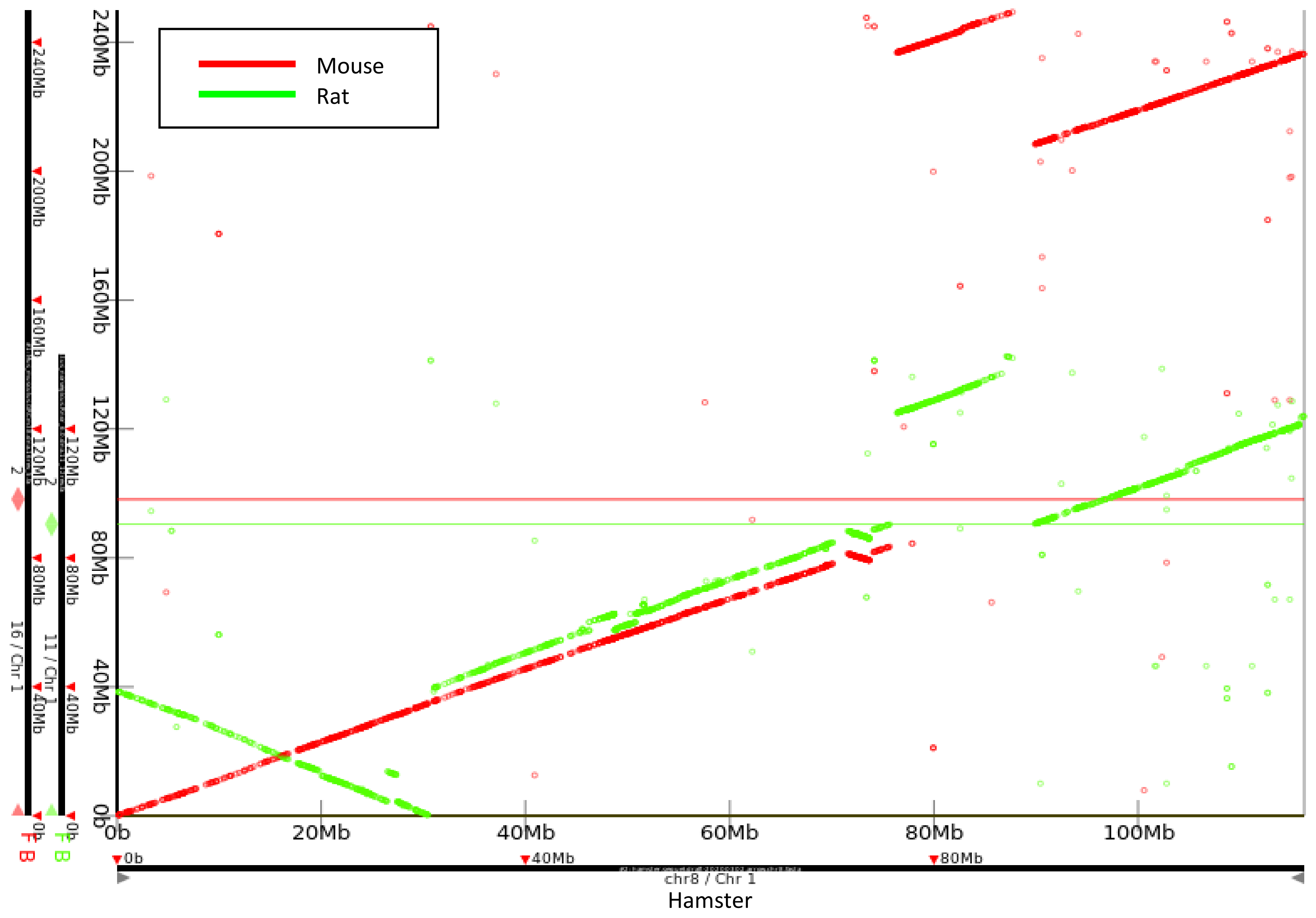

hamster	chr9	HiC_scaffold_8
Mus_Musculus	9	
Rattus_norvegicus	8	

0bp-133,307,652bp of 133,307,652bp (100.00%)

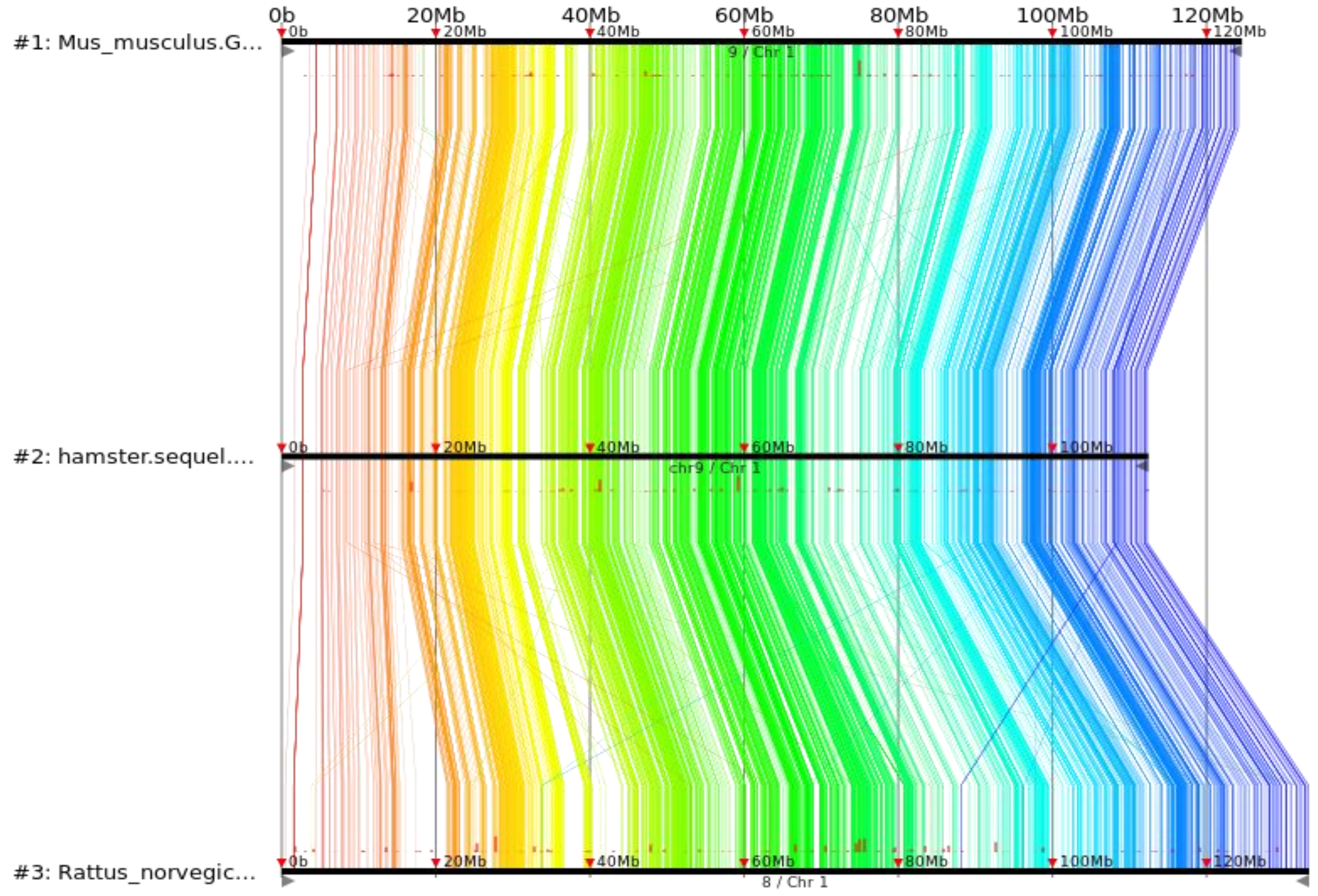

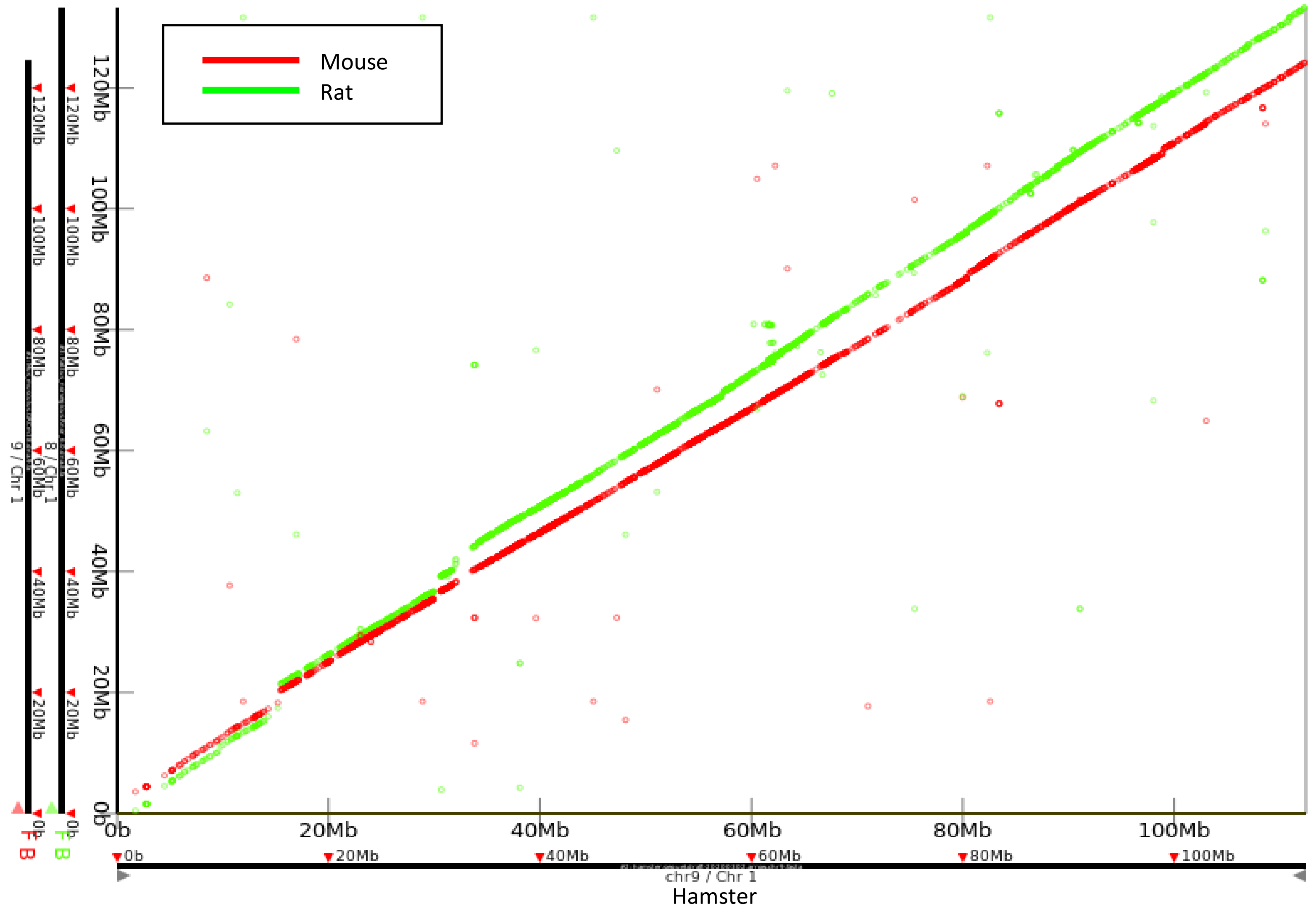

hamster	chr10	HiC_scaffold_3
Mus_Musculus	18(rev.) & 15(rev.) & 13	
Rattus_norvegicus	18(rev.) & 1 & 2(rev.)	

0bp-637,400,148bp of 637,400,148bp (100.00%)

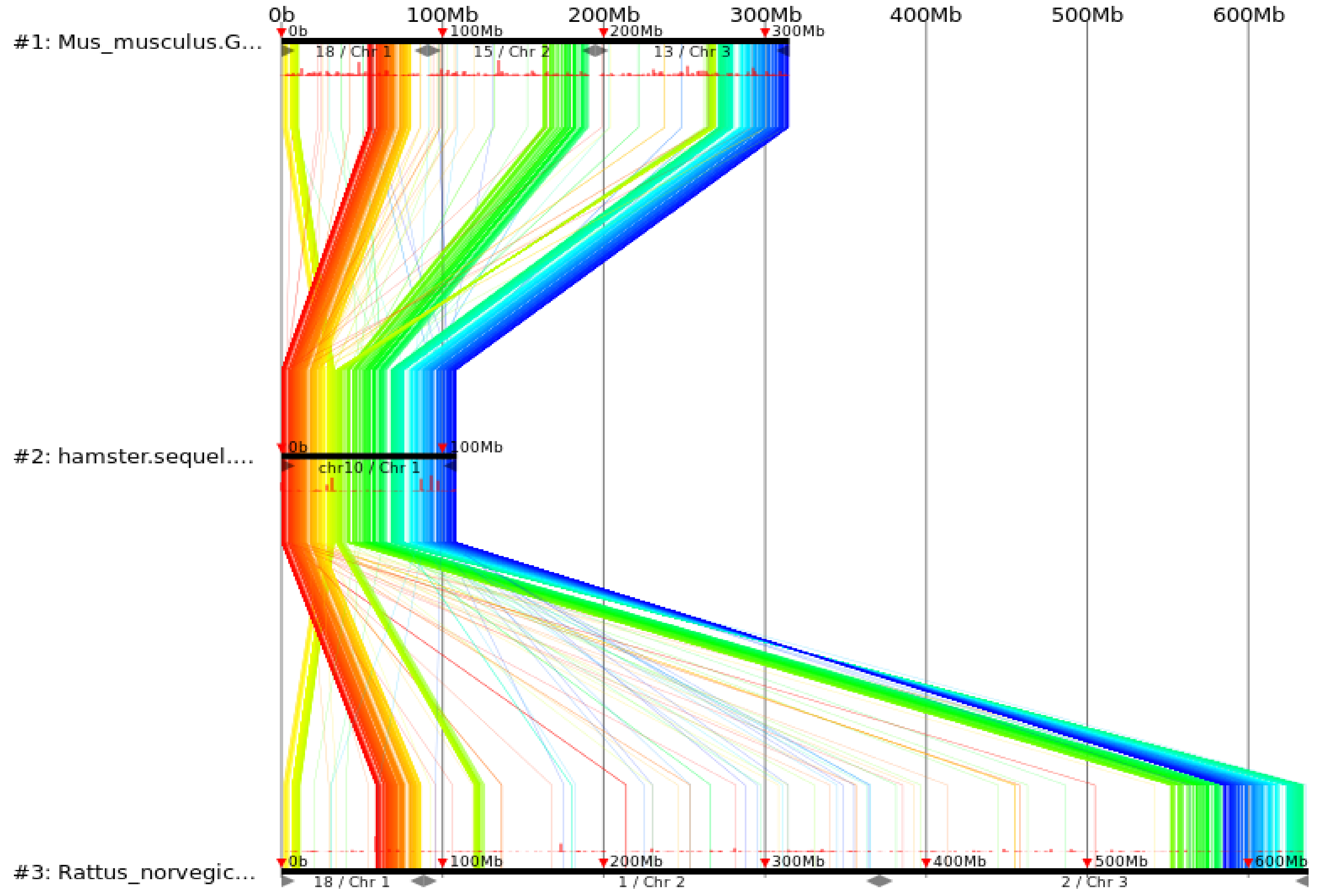

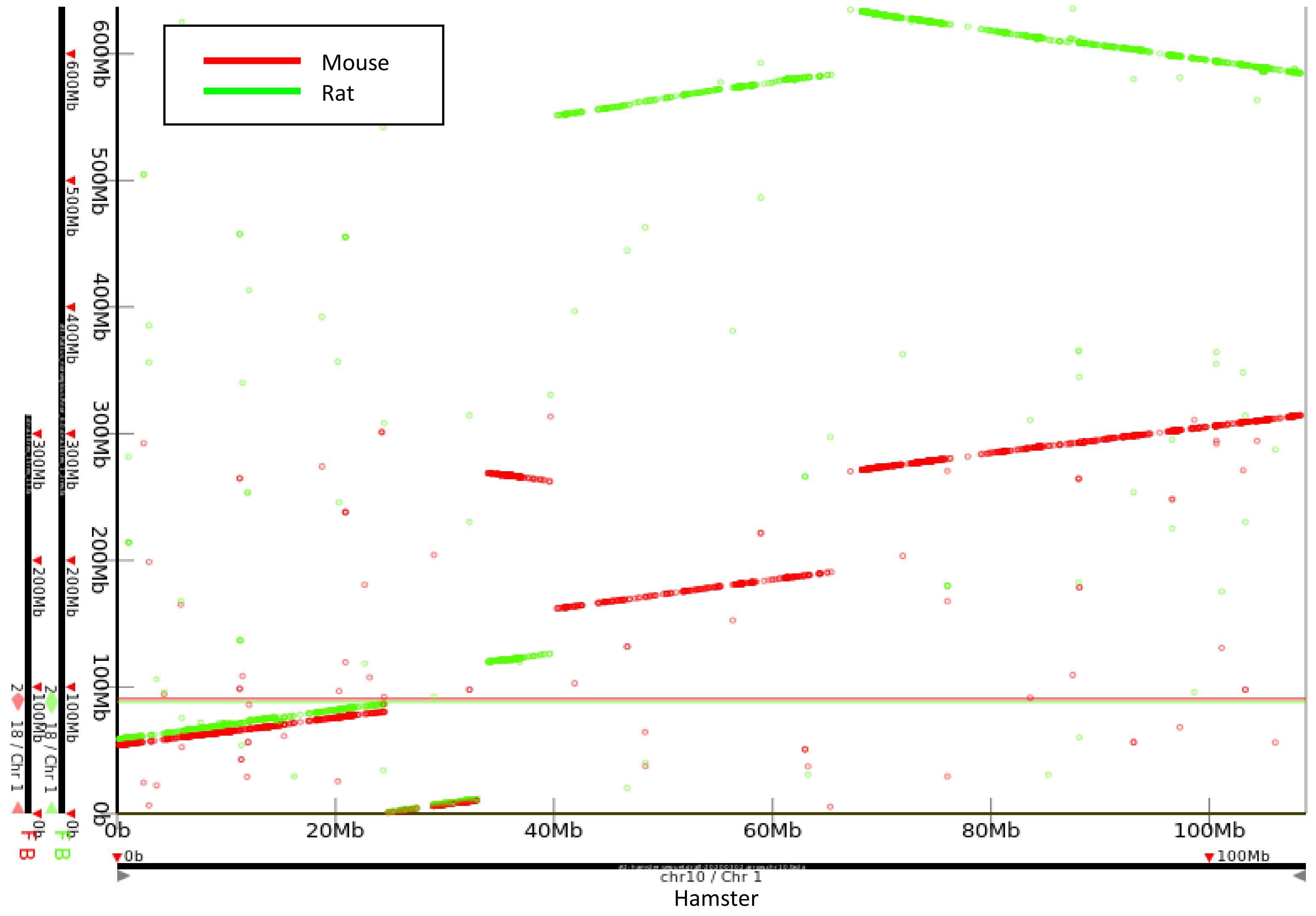

hamster	chr11	HiC_scaffold_4
Mus_Musculus	11(rev.) & 5 & 14(rev.)	
Rattus_norvegicus	14(rev.) & 15(rev.) & 16(rev.)	

0bp-398,819,491bp of 398,819,491bp (100.00%)

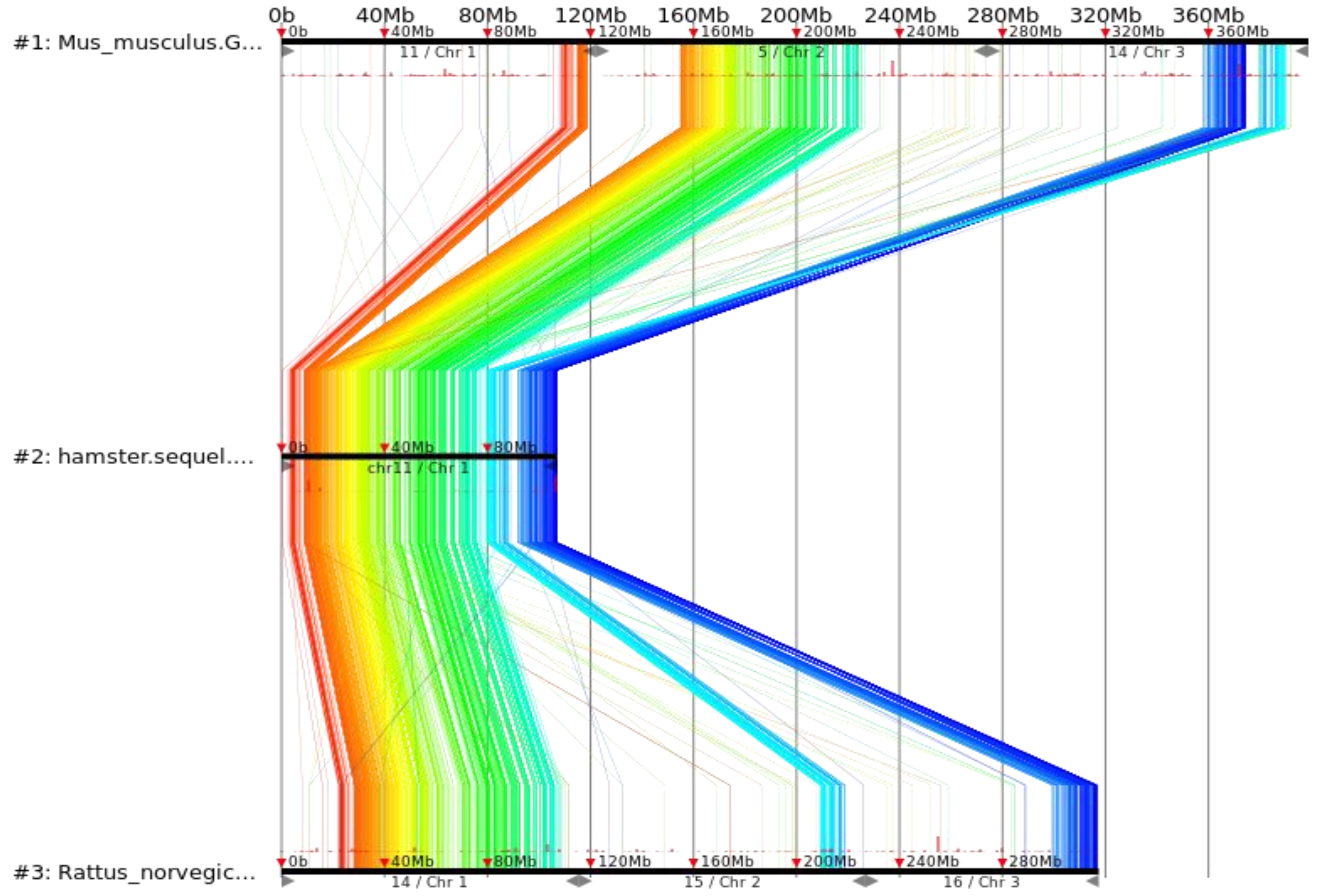

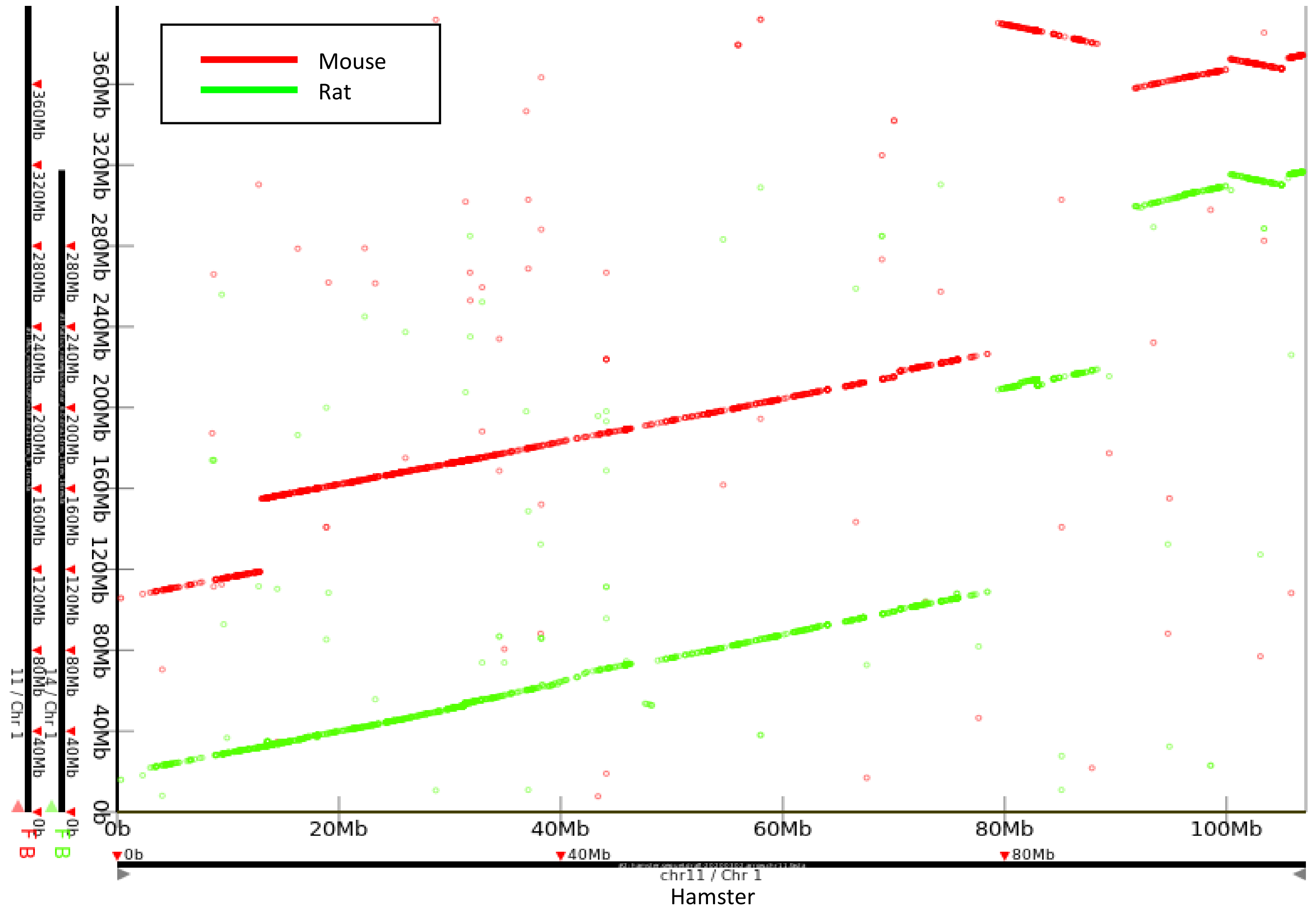

hamster	chr12	HiC_scaffold_12
Mus_Musculus	1(rev.) & 17 & 10	
Rattus_norvegicus	13(rev.) & 6 & 7(rev.)	

0bp-421,154,255bp of 421,154,255bp (100.00%)

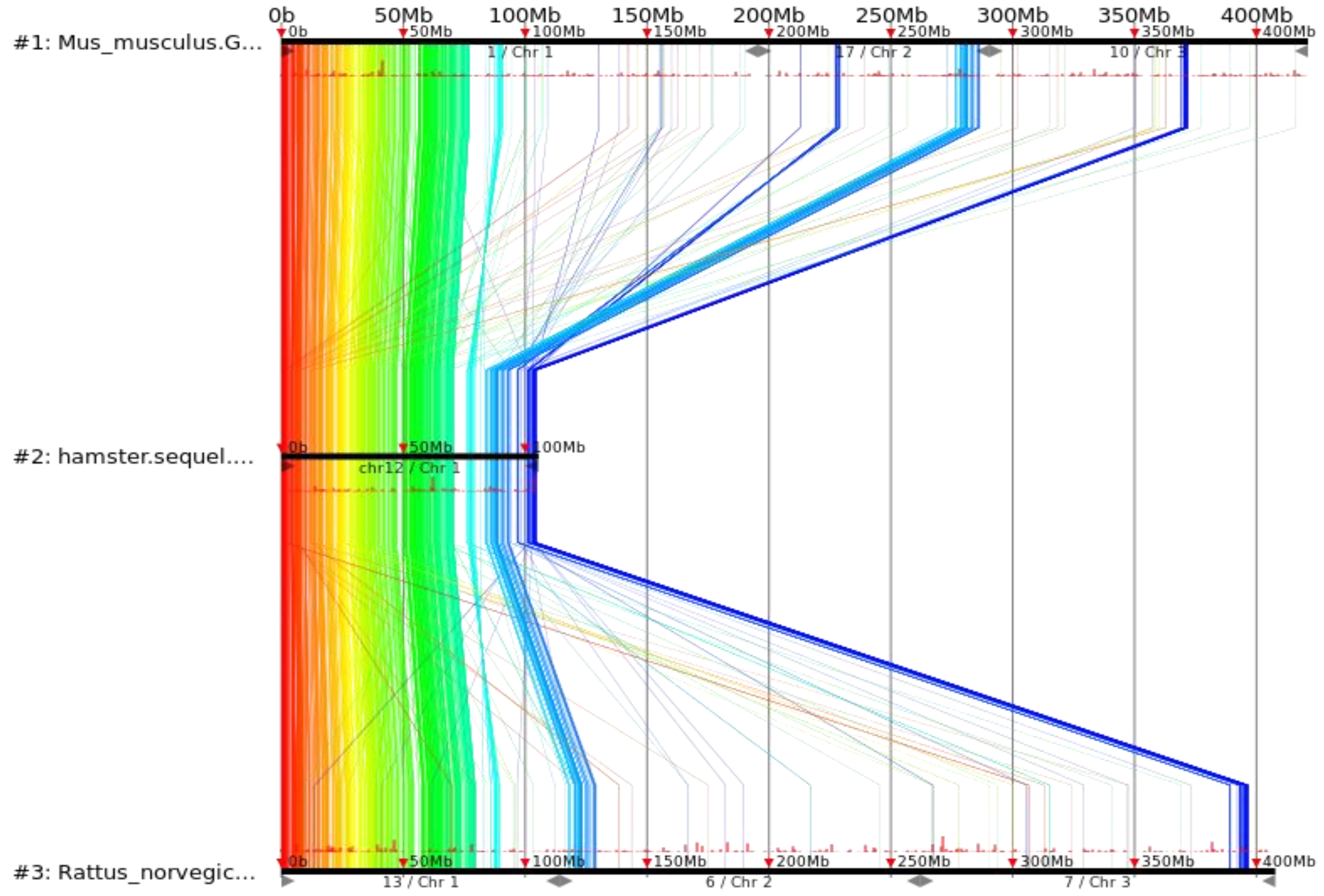

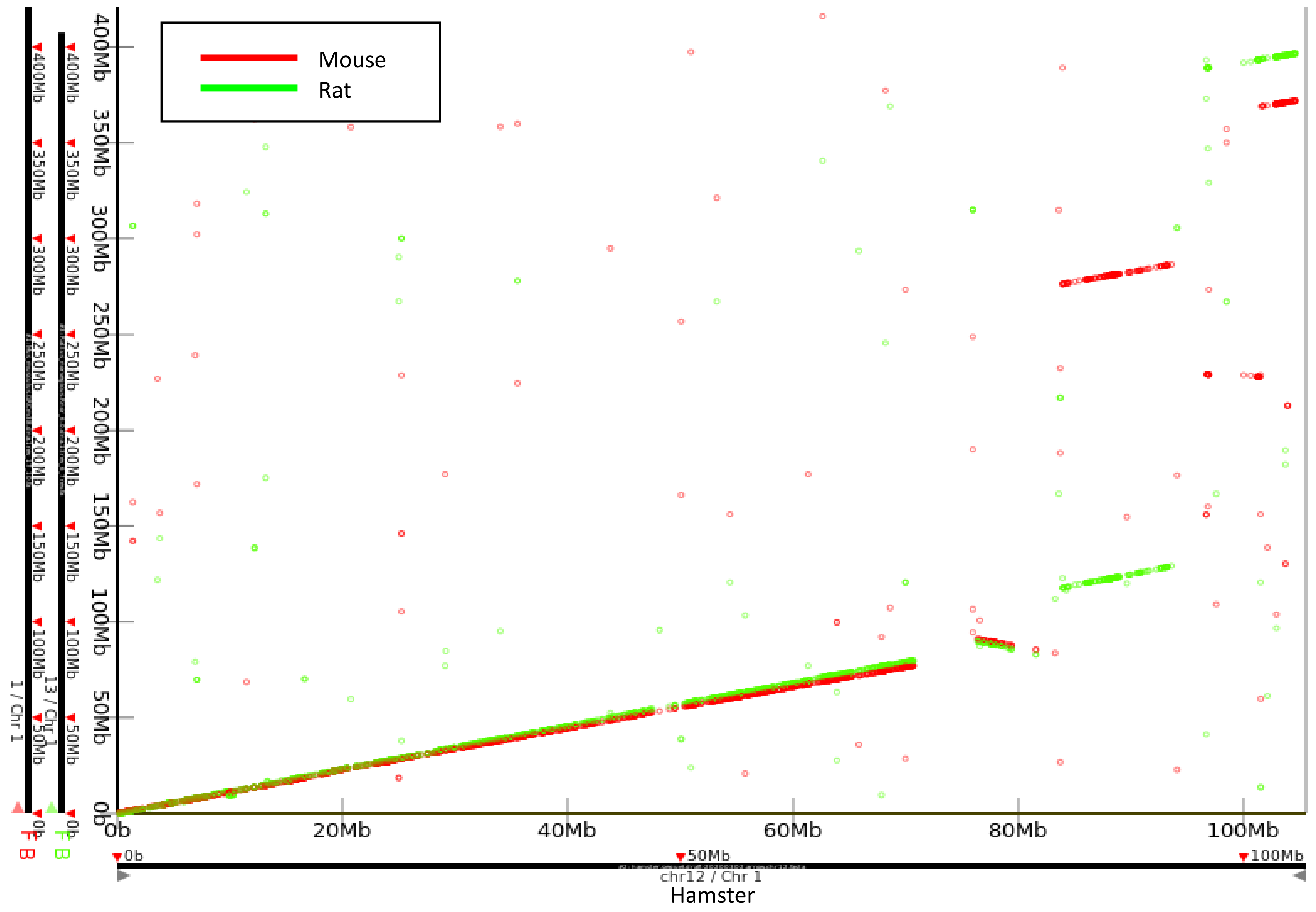

hamster	chr13	HiC_scaffold_18
Mus_Musculus	5 & 6	
Rattus_norvegicus	4	

0bp-301,571,240bp of 301,571,240bp (100.00%)

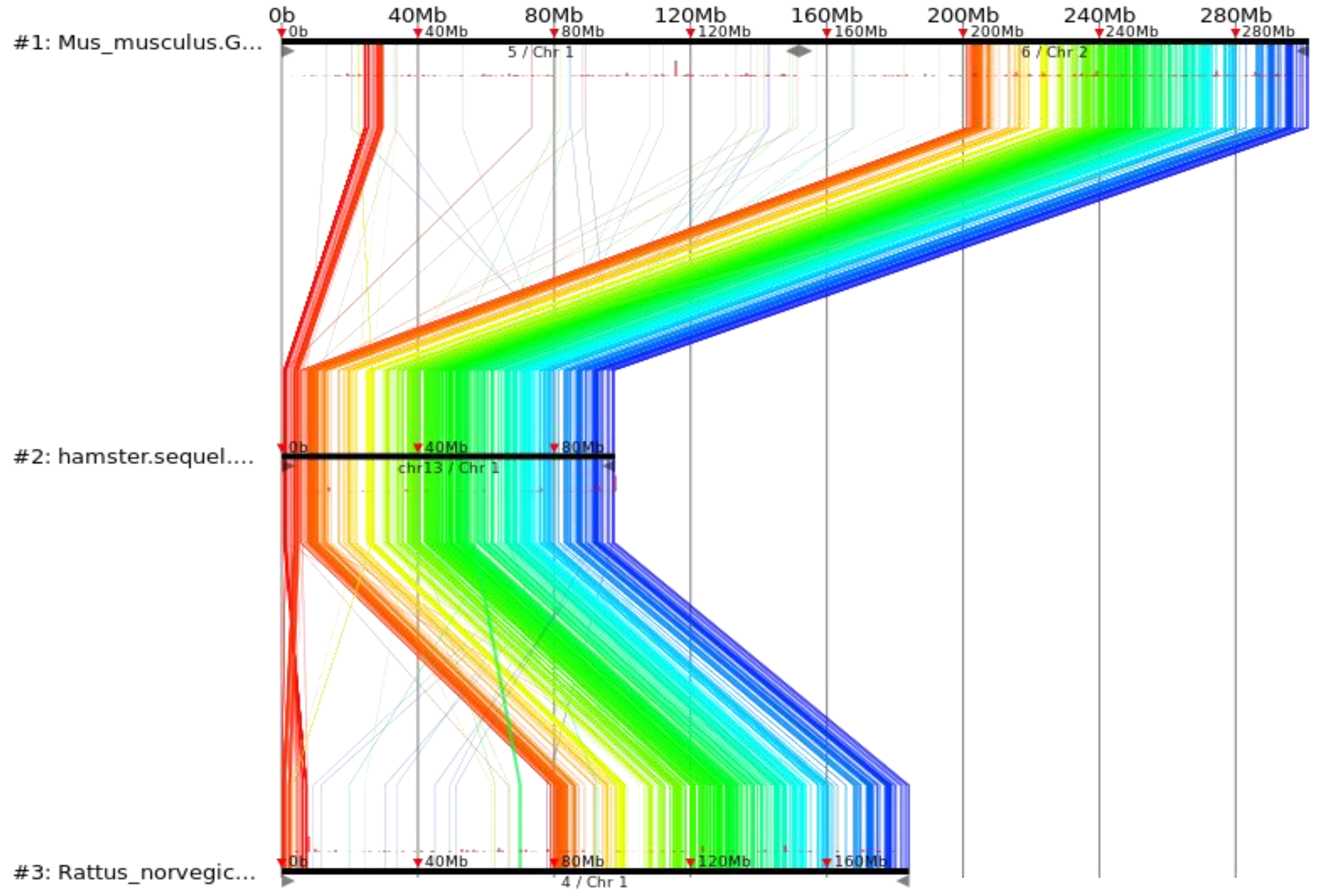

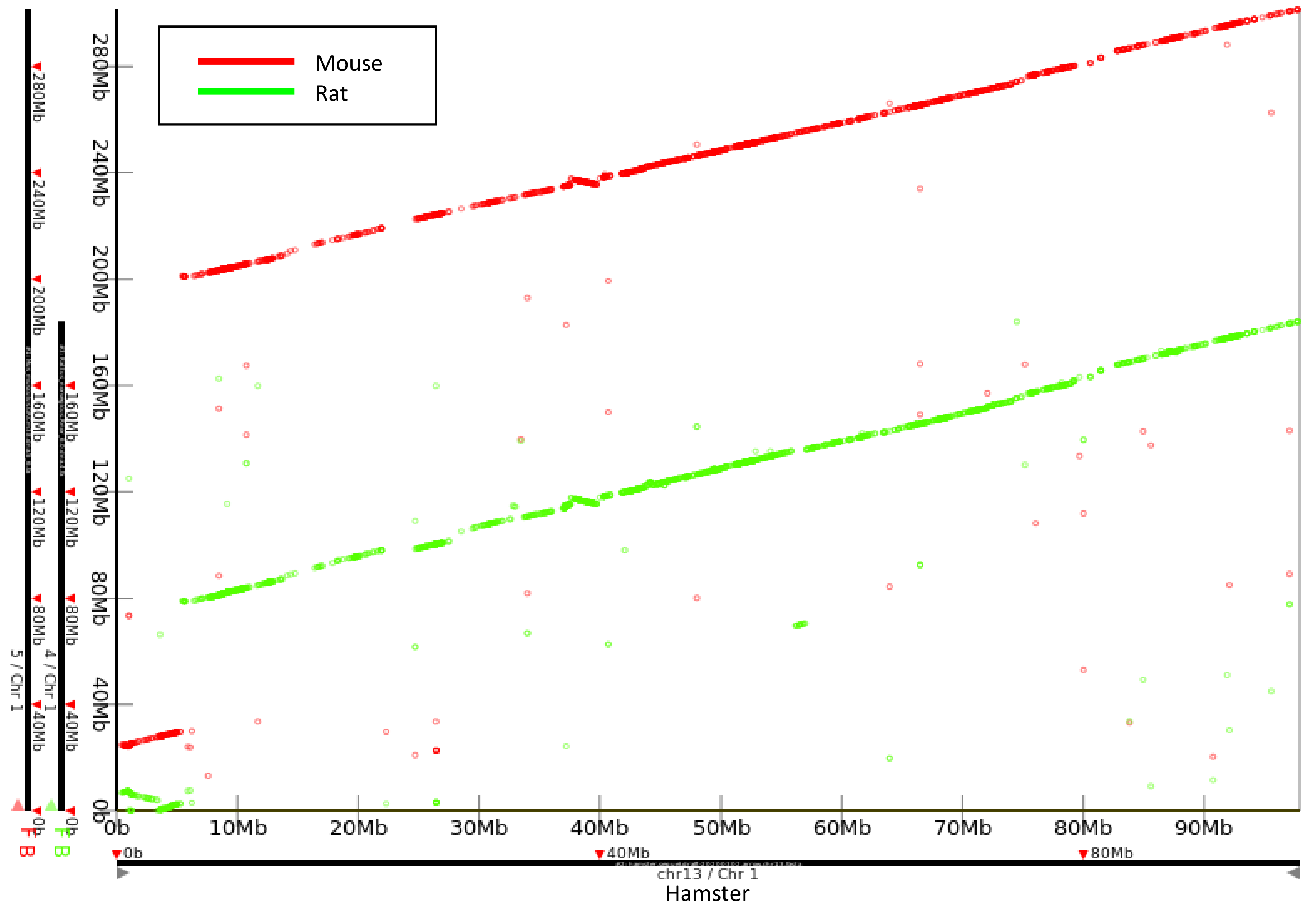

hamster	chr14	HiC_scaffold_20
Mus_Musculus	3	
Rattus_norvegicus	2	

0bp-266,435,125bp of 266,435,125bp (100.00%)

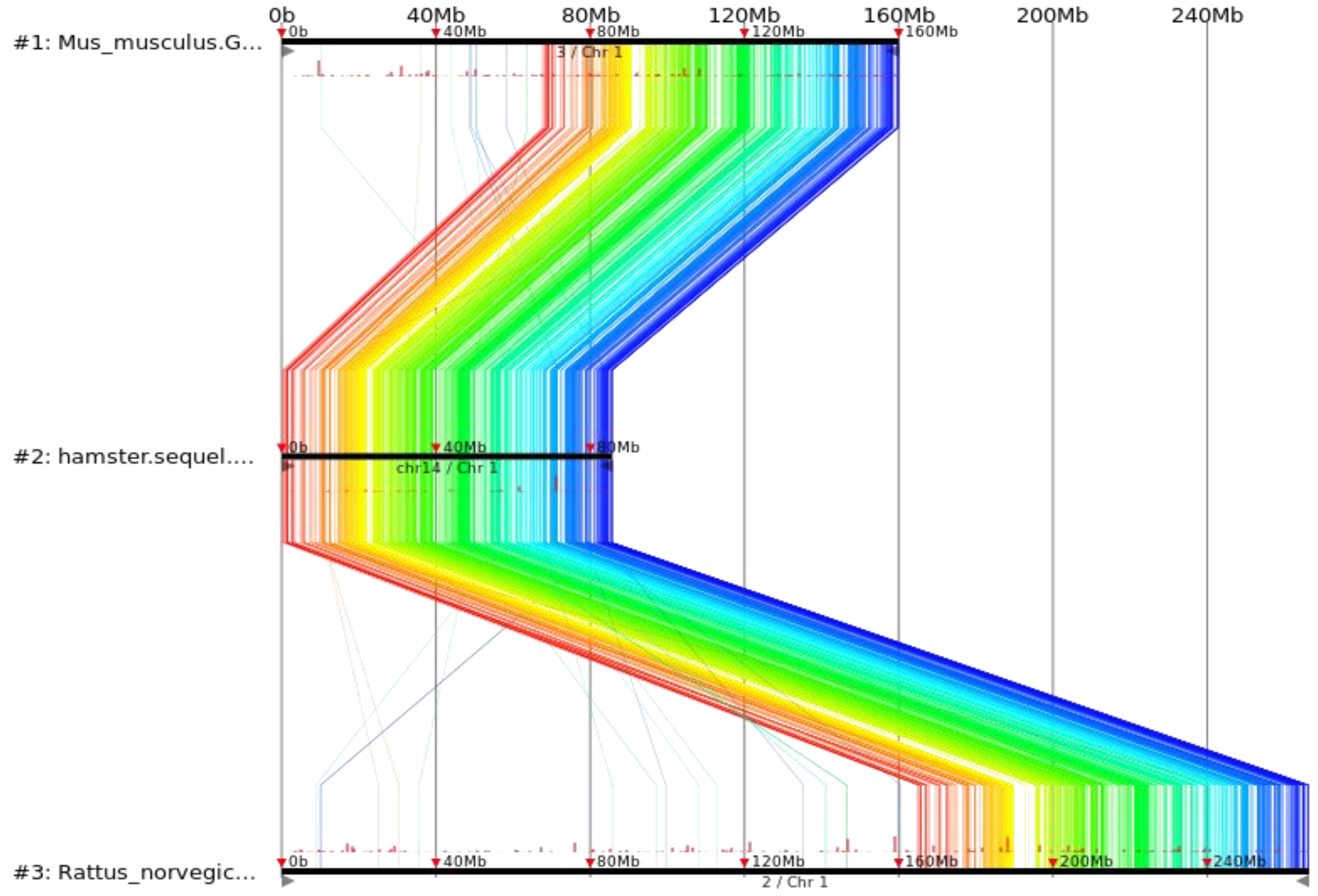

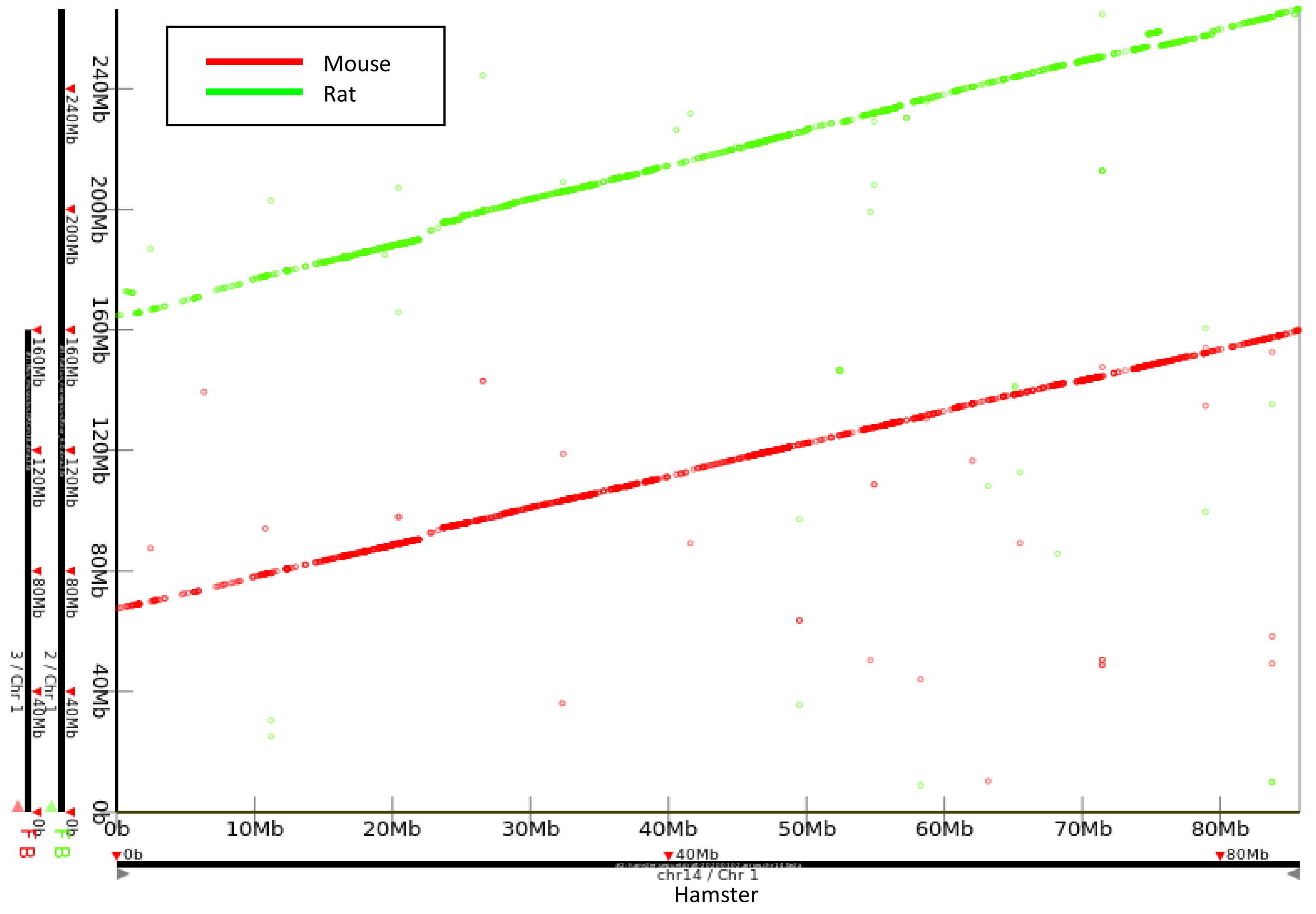

hamster	chr15	HiC_scaffold_6
Mus_Musculus	12(rev.)	
Rattus_norvegicus	6(rev.)	

0bp-147,991,367bp of 147,991,367bp (100.00%)

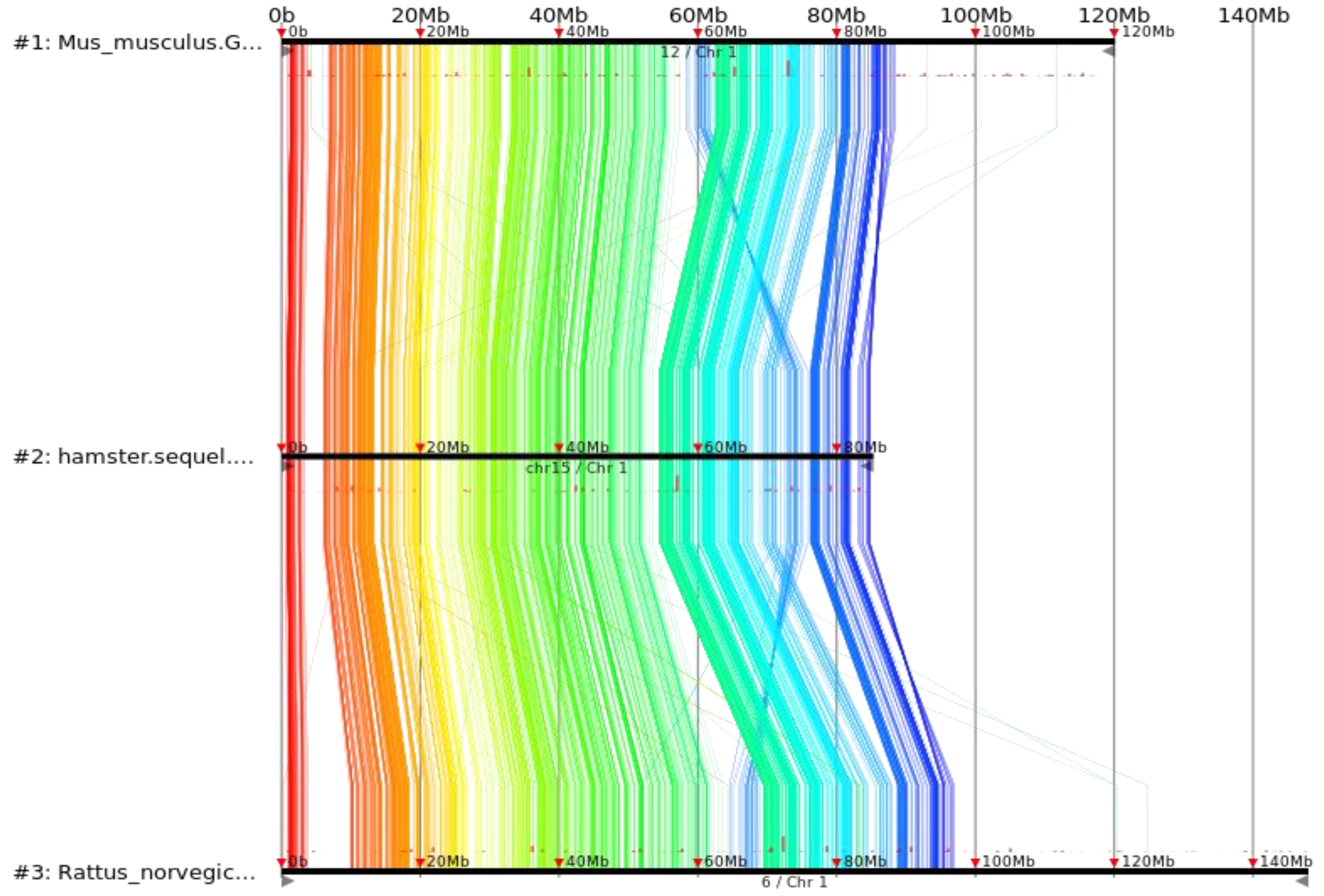

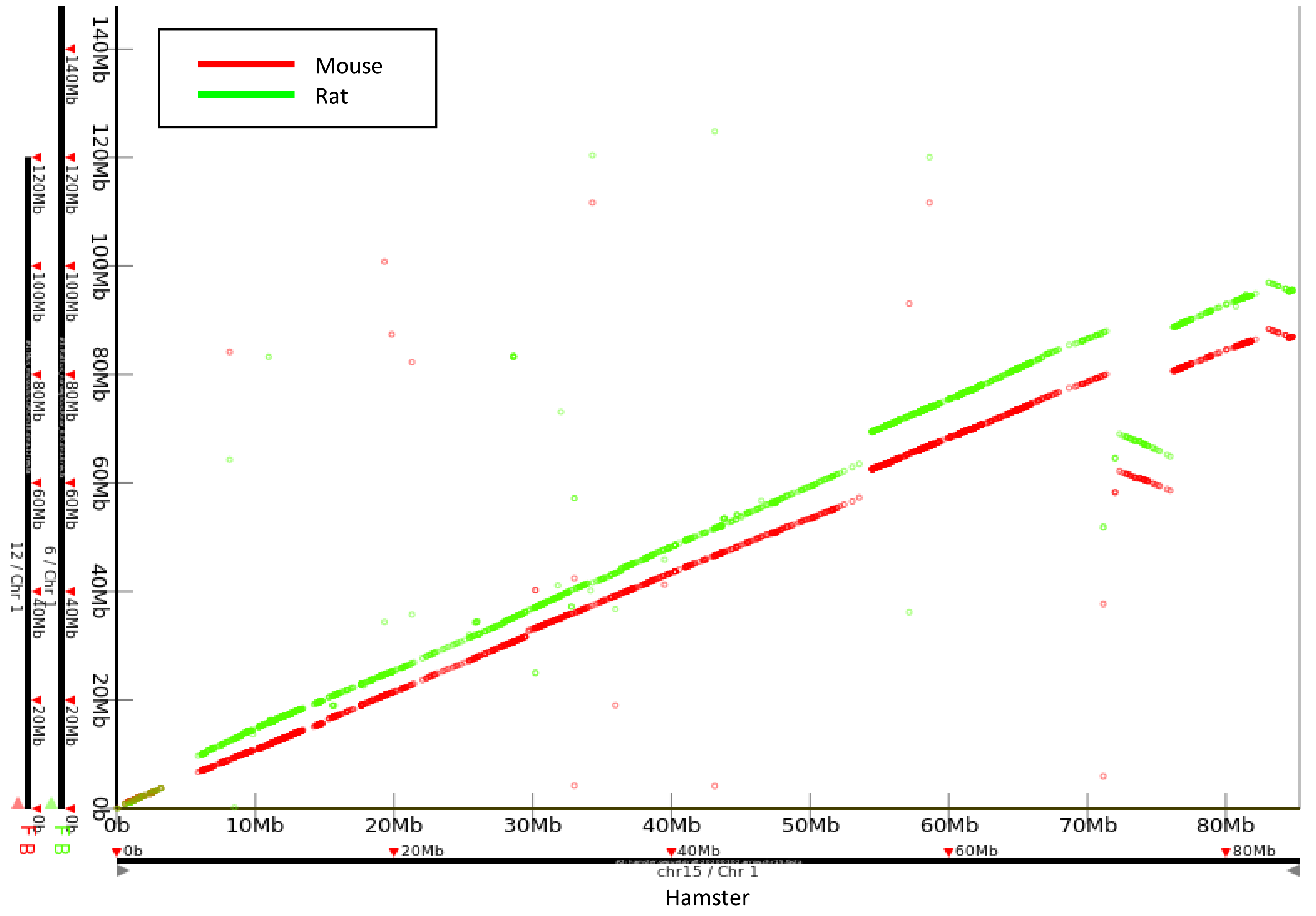

hamster	chr16	HiC_scaffold_15
Mus_Musculus	3(rev.) & 10(rev.)	
Rattus_norvegicus	2(rev.) & 7(rev.)	

0bp-412,164,437bp of 412,164,437bp (100.00%)

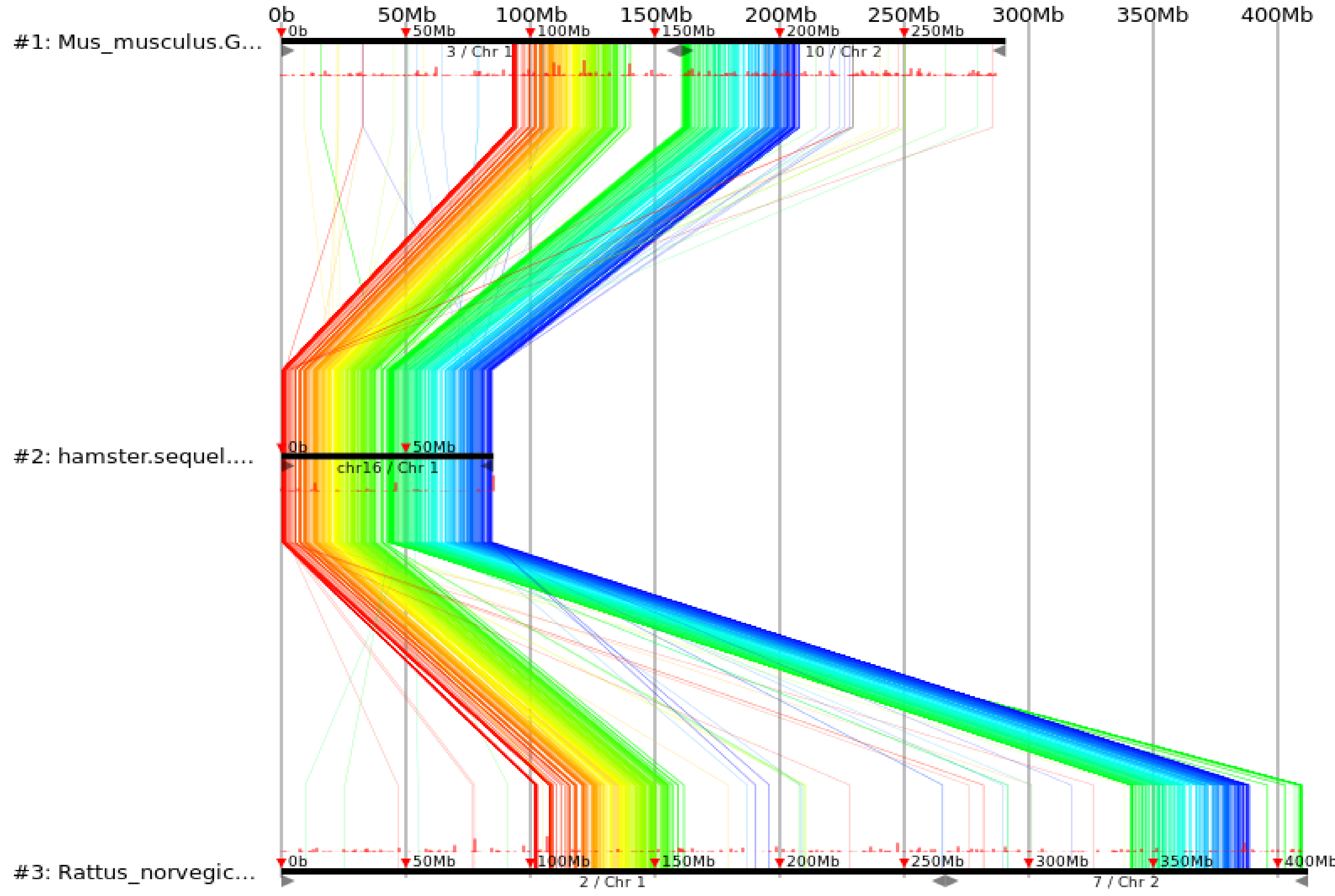

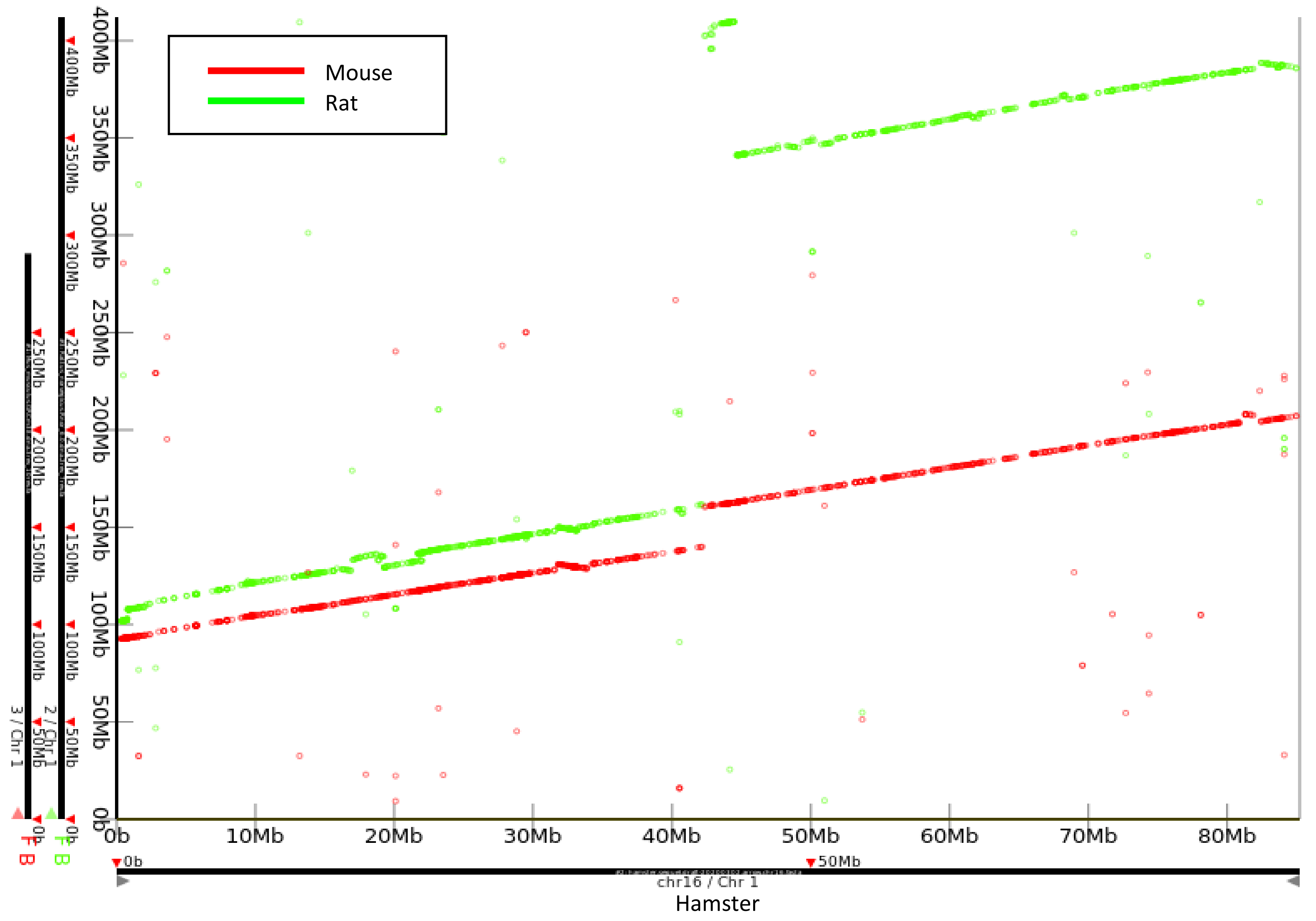

hamster	chr17	HiC_scaffold_11
Mus_Musculus	4(rev.)	
Rattus_norvegicus	5(rev.)	

0bp-173,707,219bp of 173,707,219bp (100.00%)

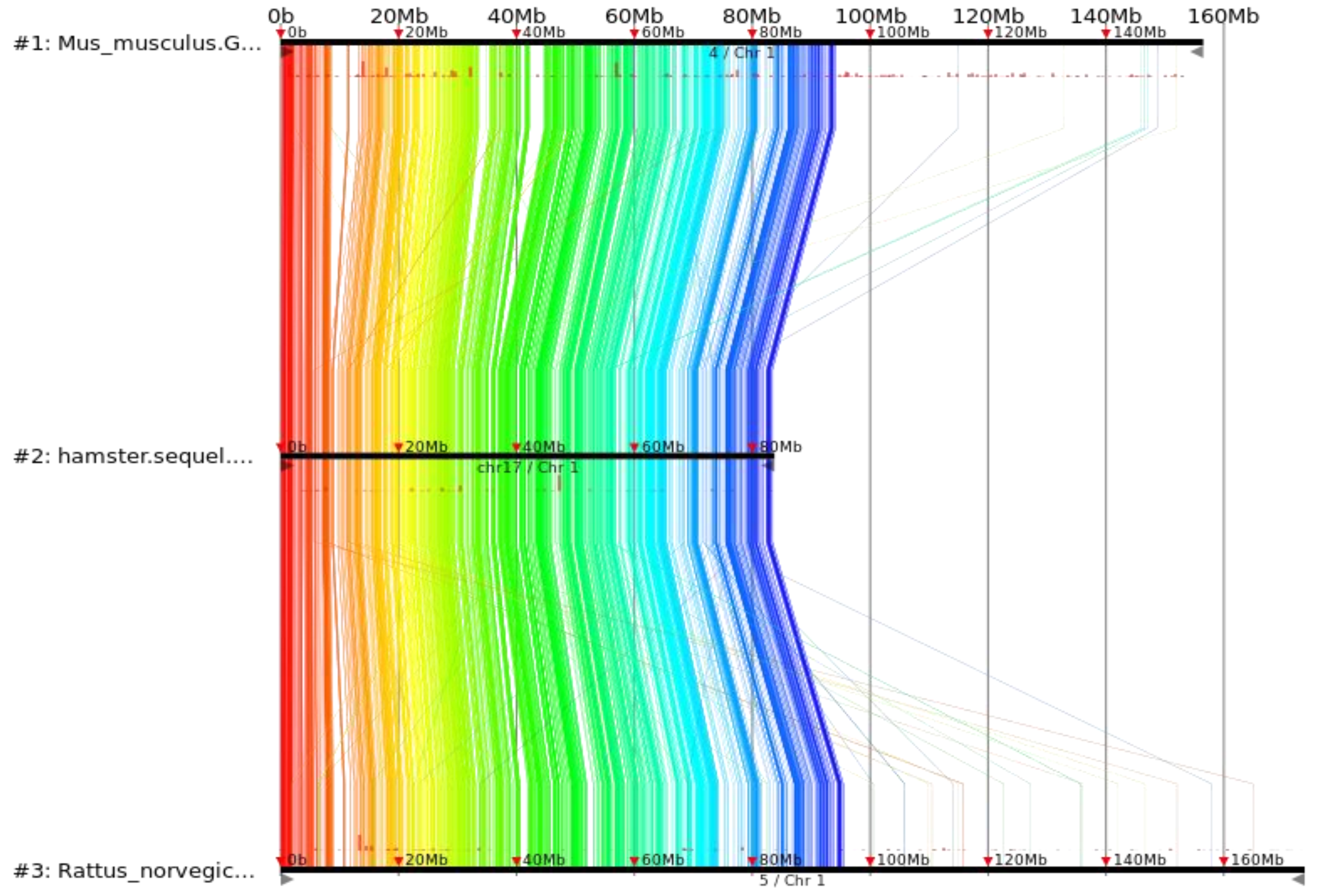

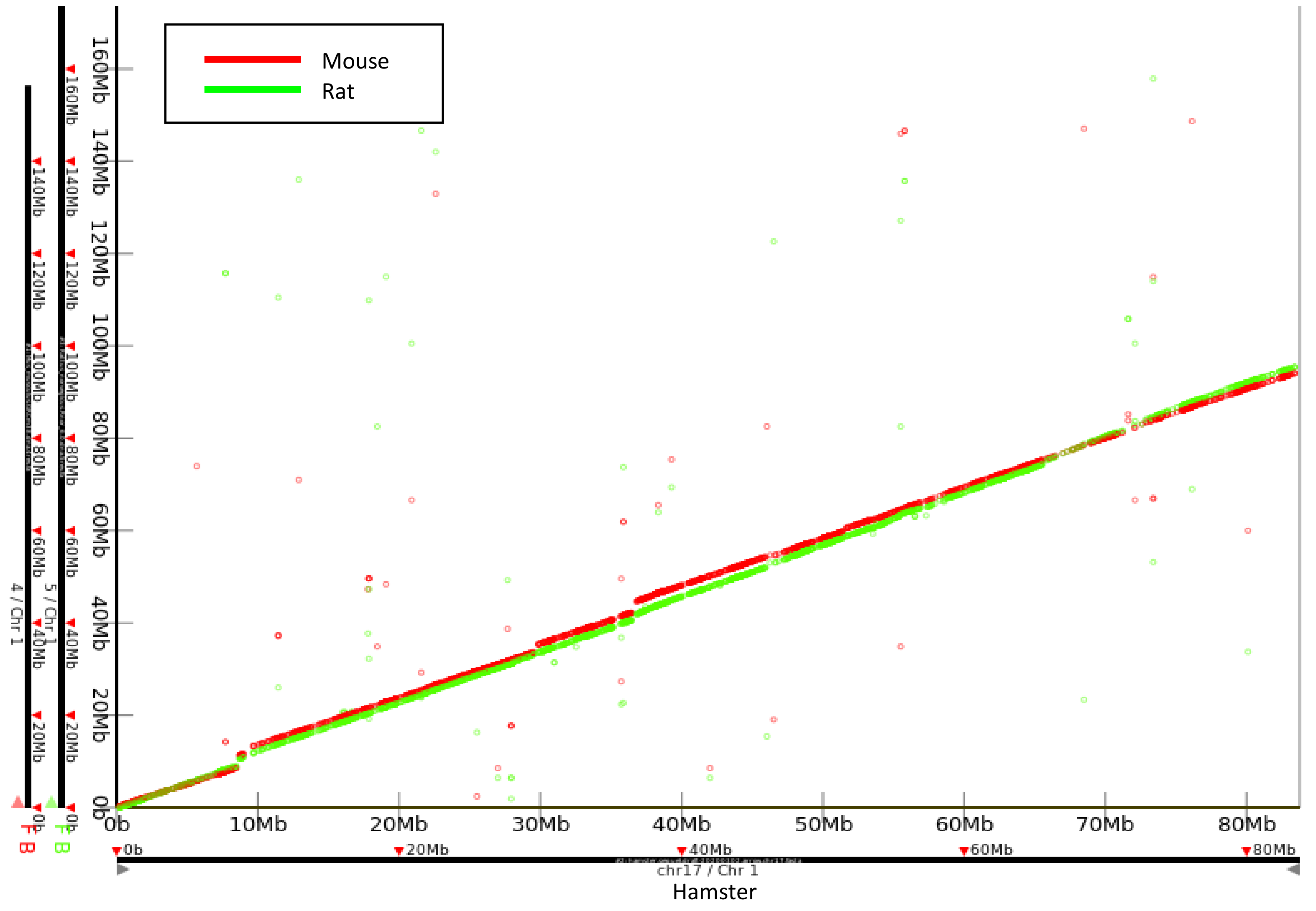

hamster	chr18	HiC_scaffold_21
Mus_Musculus	14(rev.)	
Rattus_norvegicus	15(rev.)	

0bp-124,902,244bp of 124,902,244bp (100.00%)

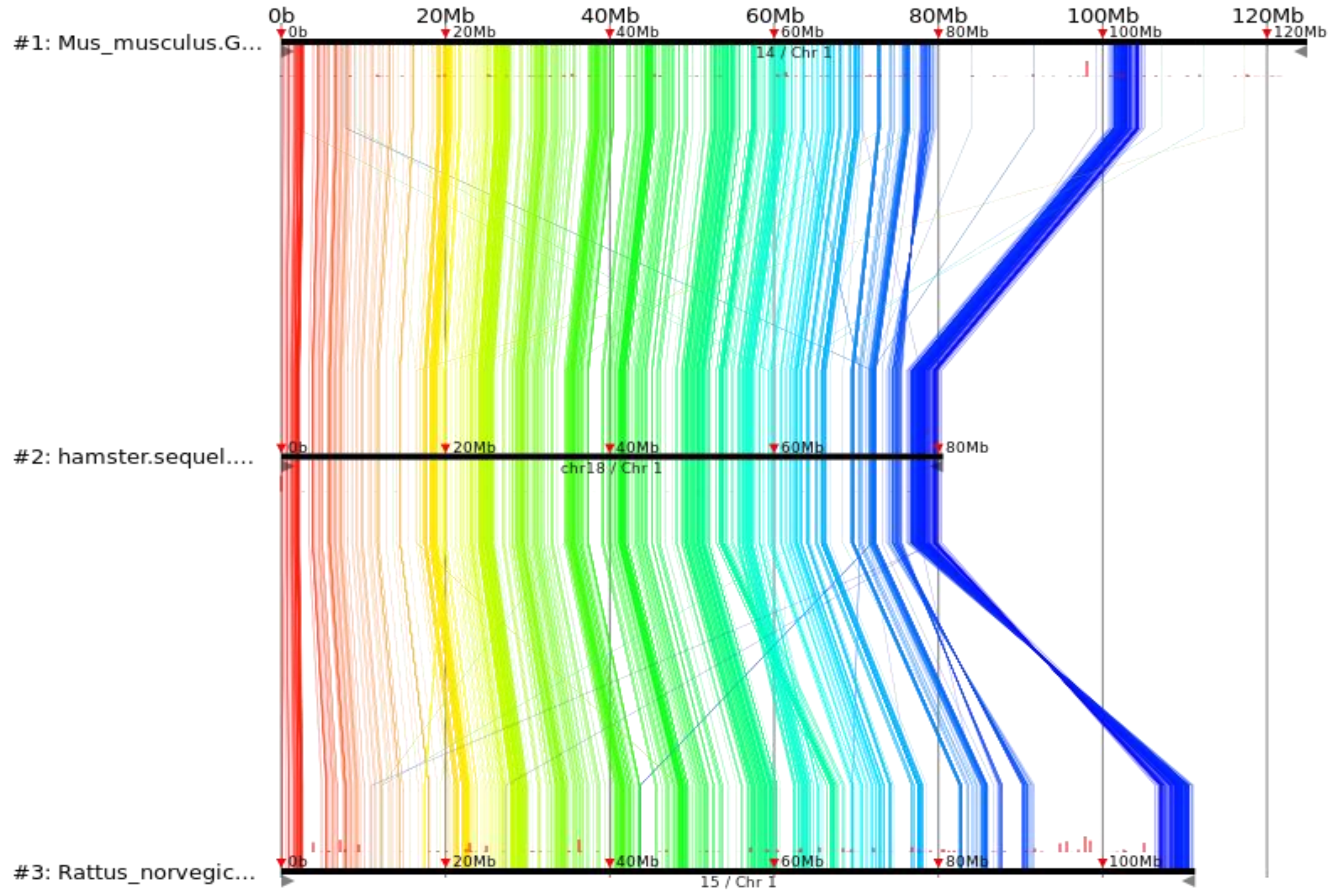

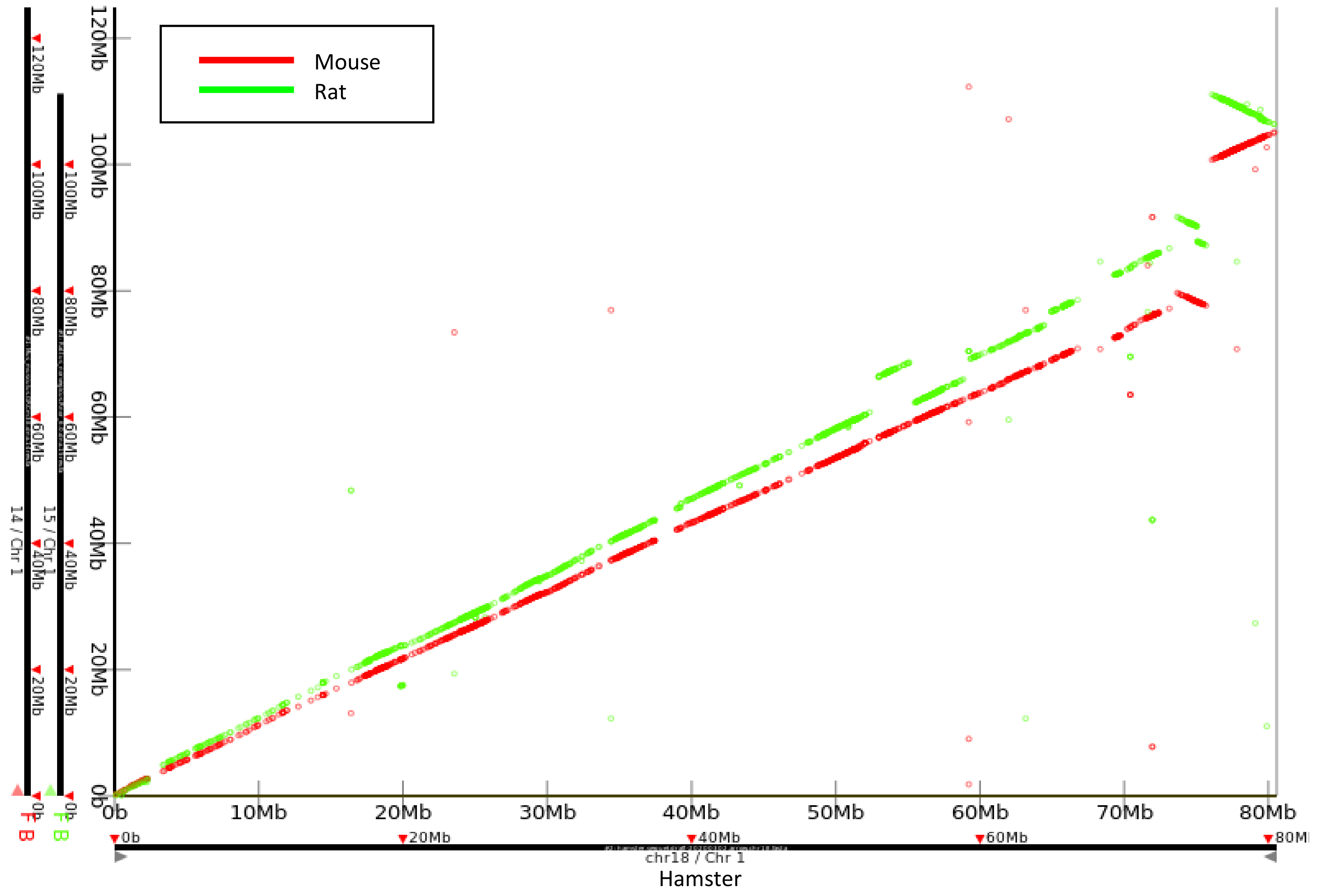

hamster	chr19	HiC_scaffold_19
Mus_Musculus	17(rev.) & 10 & 11	
Rattus_norvegicus	1(rev.) & 20 & 14	

0bp-454,462,496bp of 454,462,496bp (100.00%)

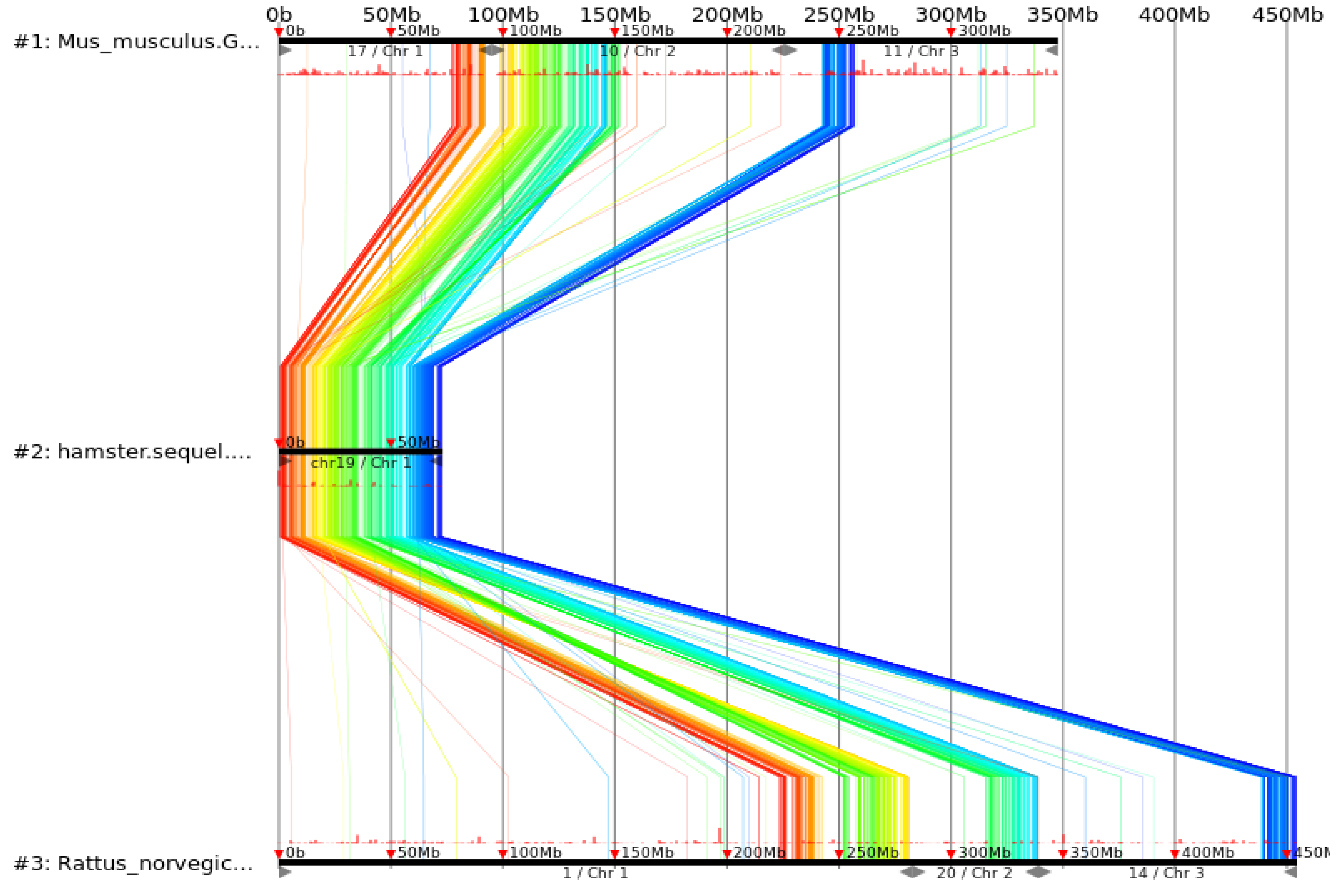

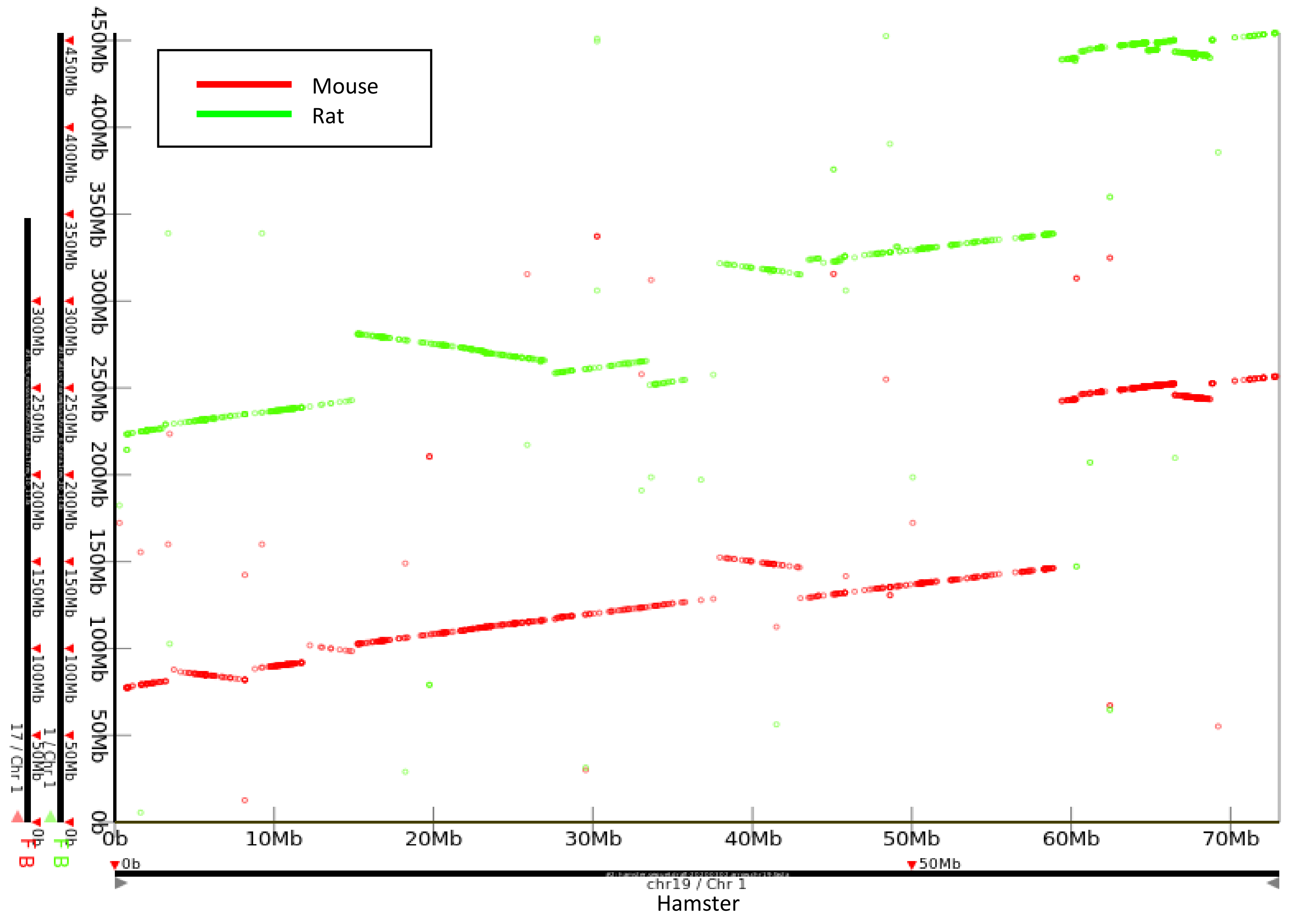

hamster	chr20	HiC_scaffold_2
Mus_Musculus	15	
Rattus_norvegicus	7	

0bp-145,729,302bp of 145,729,302bp (100.00%)

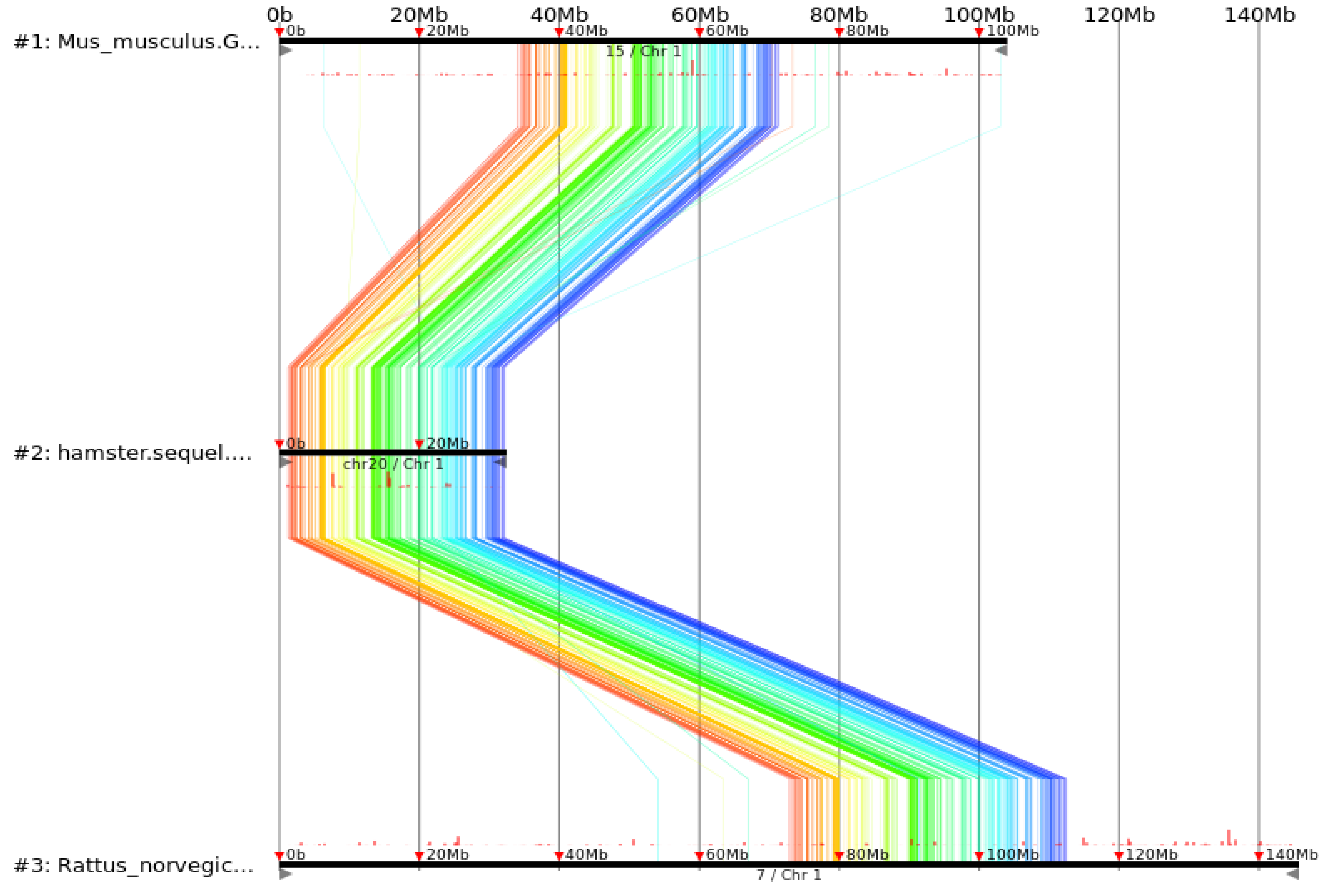

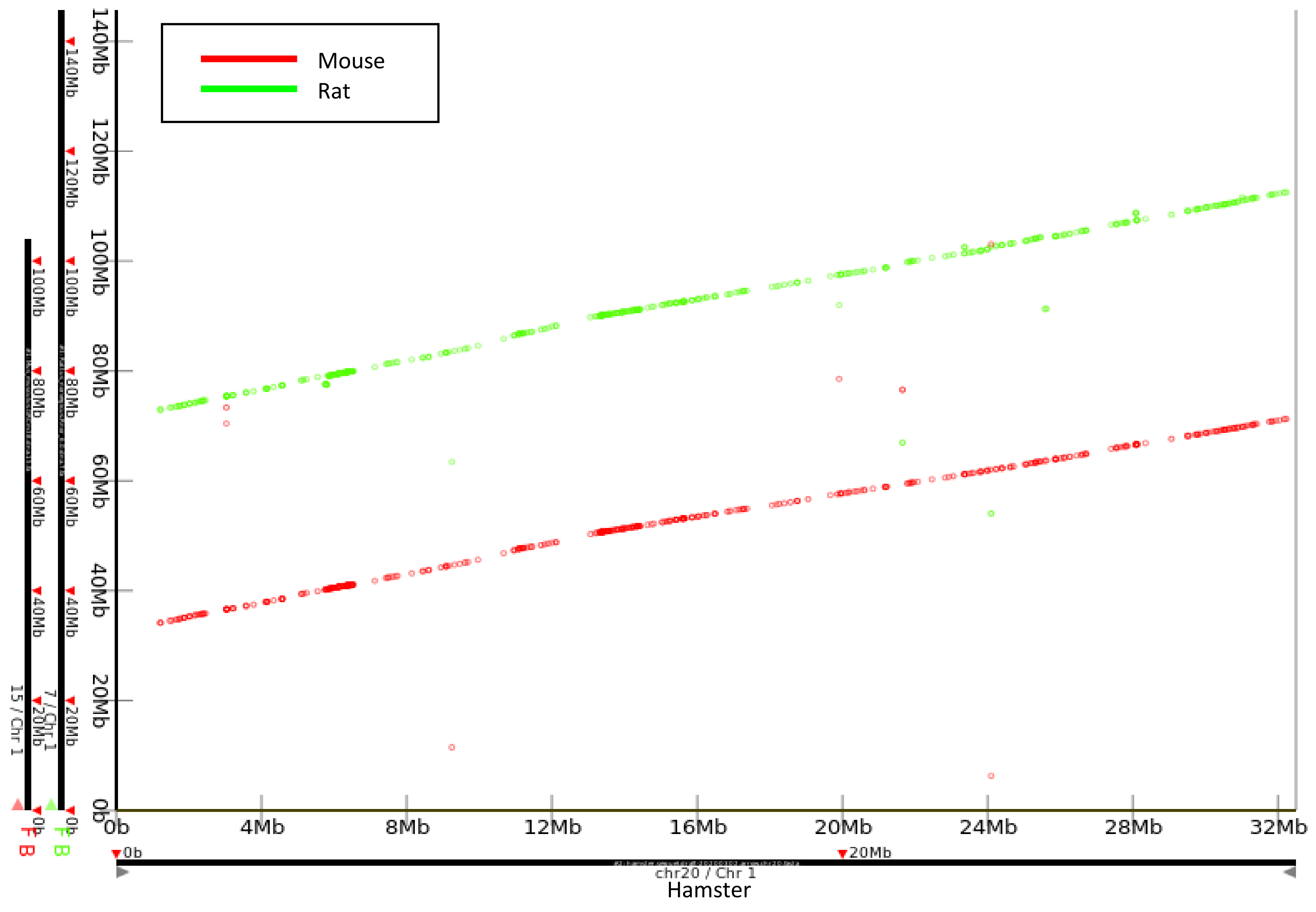

hamster	chr21	HiC_scaffold_7
Mus_Musculus	7	
Rattus_norvegicus	1	

0bp-282,763,074bp of 282,763,074bp (100.00%)

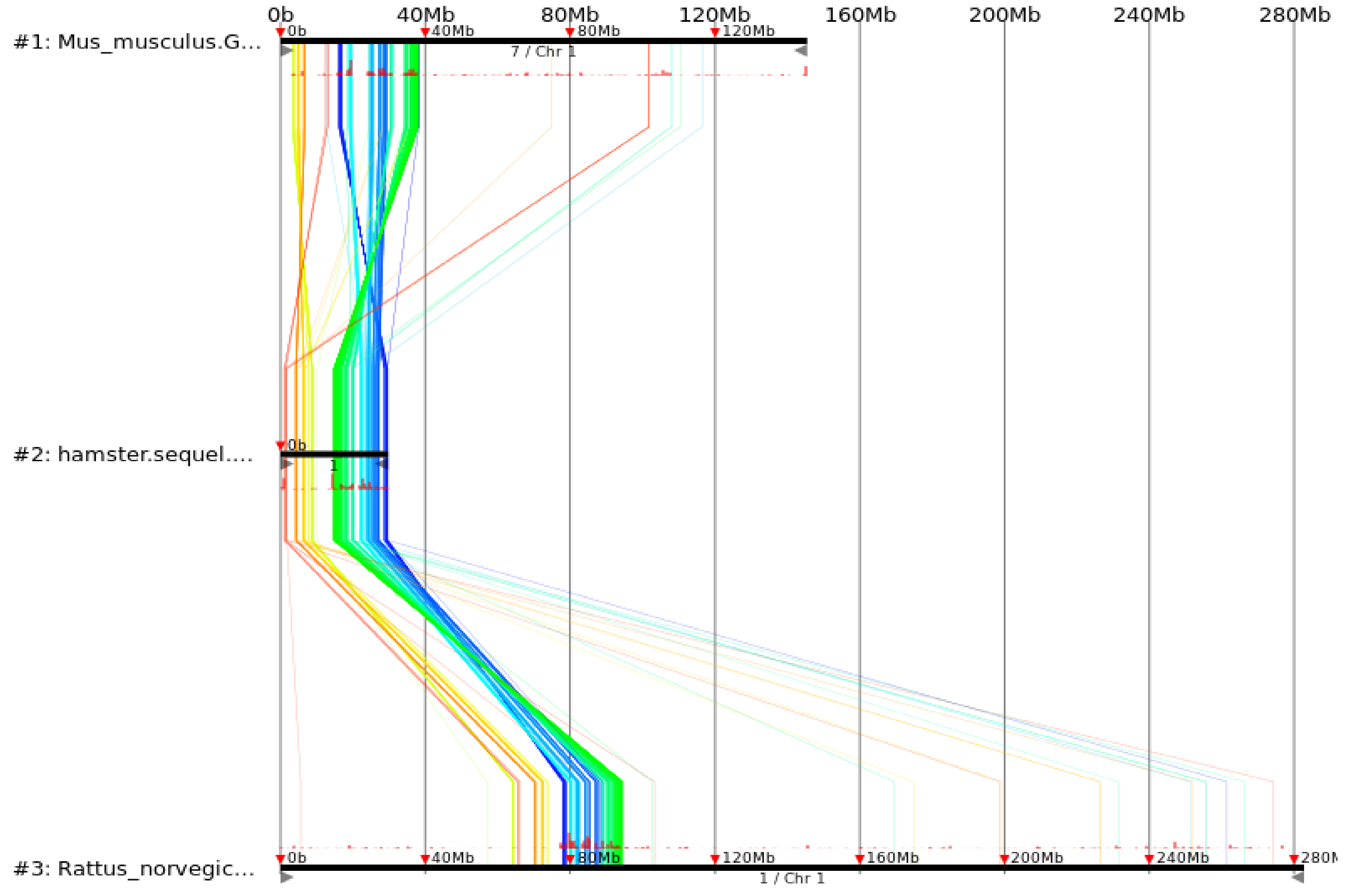

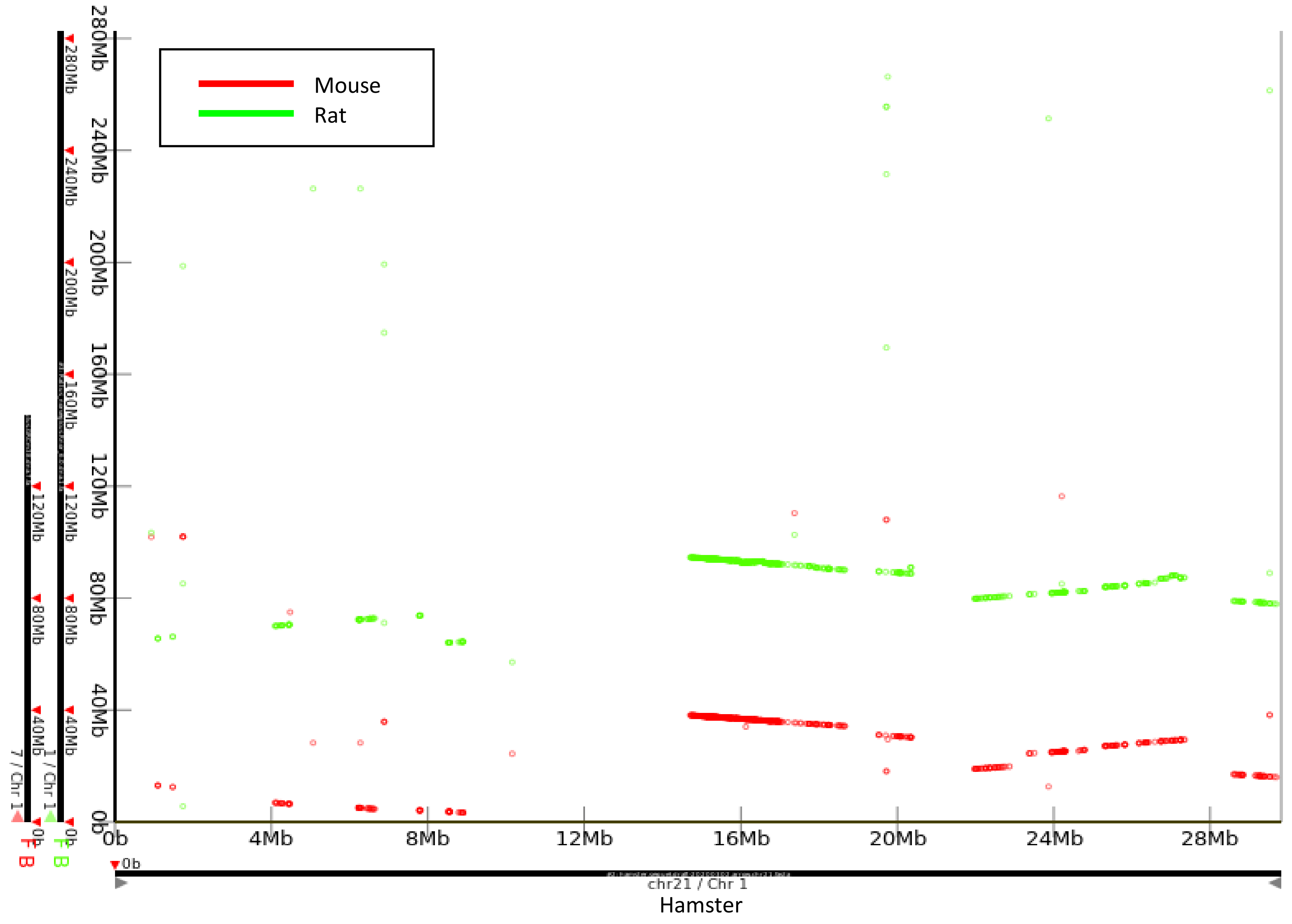

hamster	chr22	HiC_scaffold_22
Mus_Musculus	X (rev.)	
Rattus_norvegicus	X (rev.)	

0bp-171,031,299bp of 171,031,299bp (100.00%)

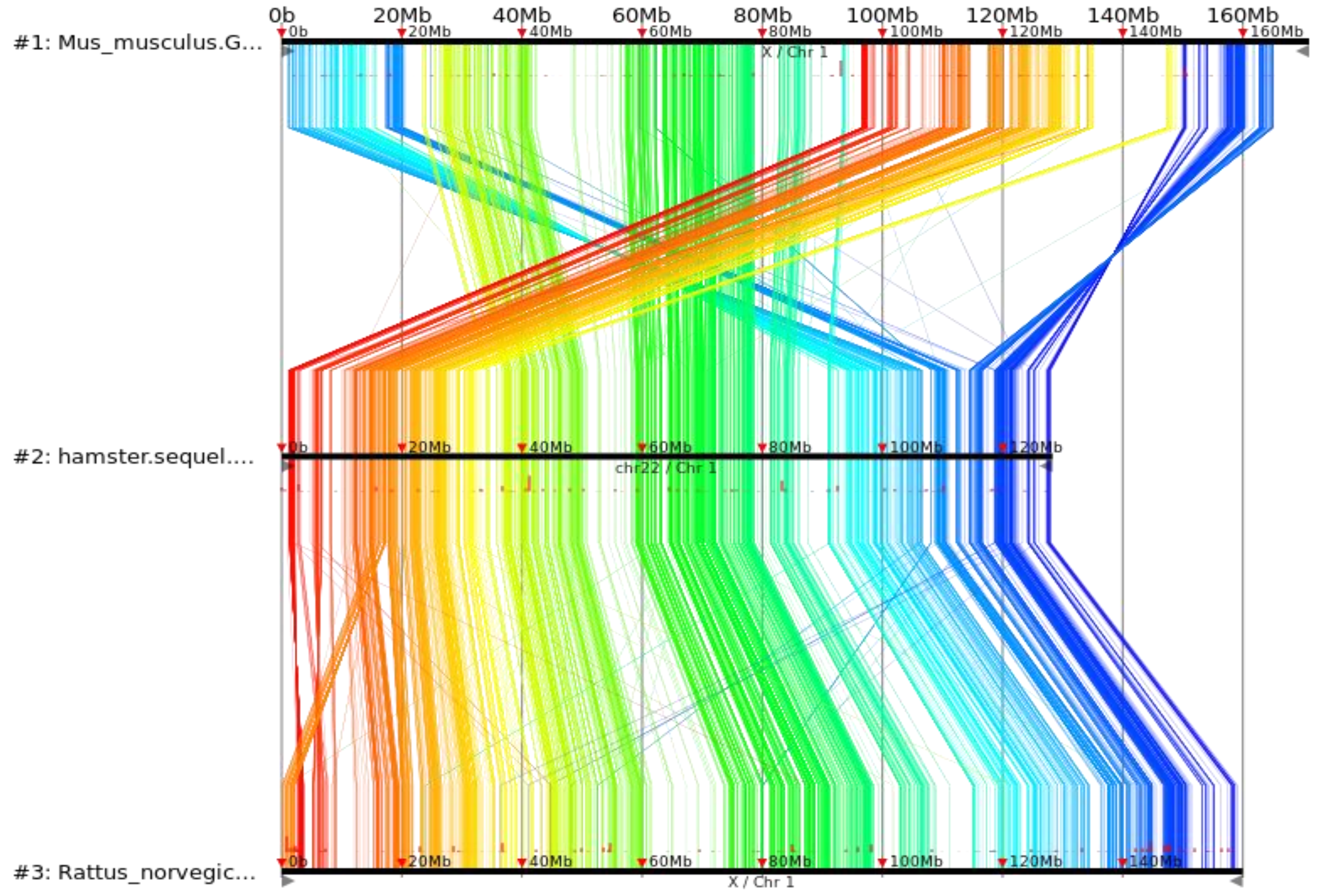

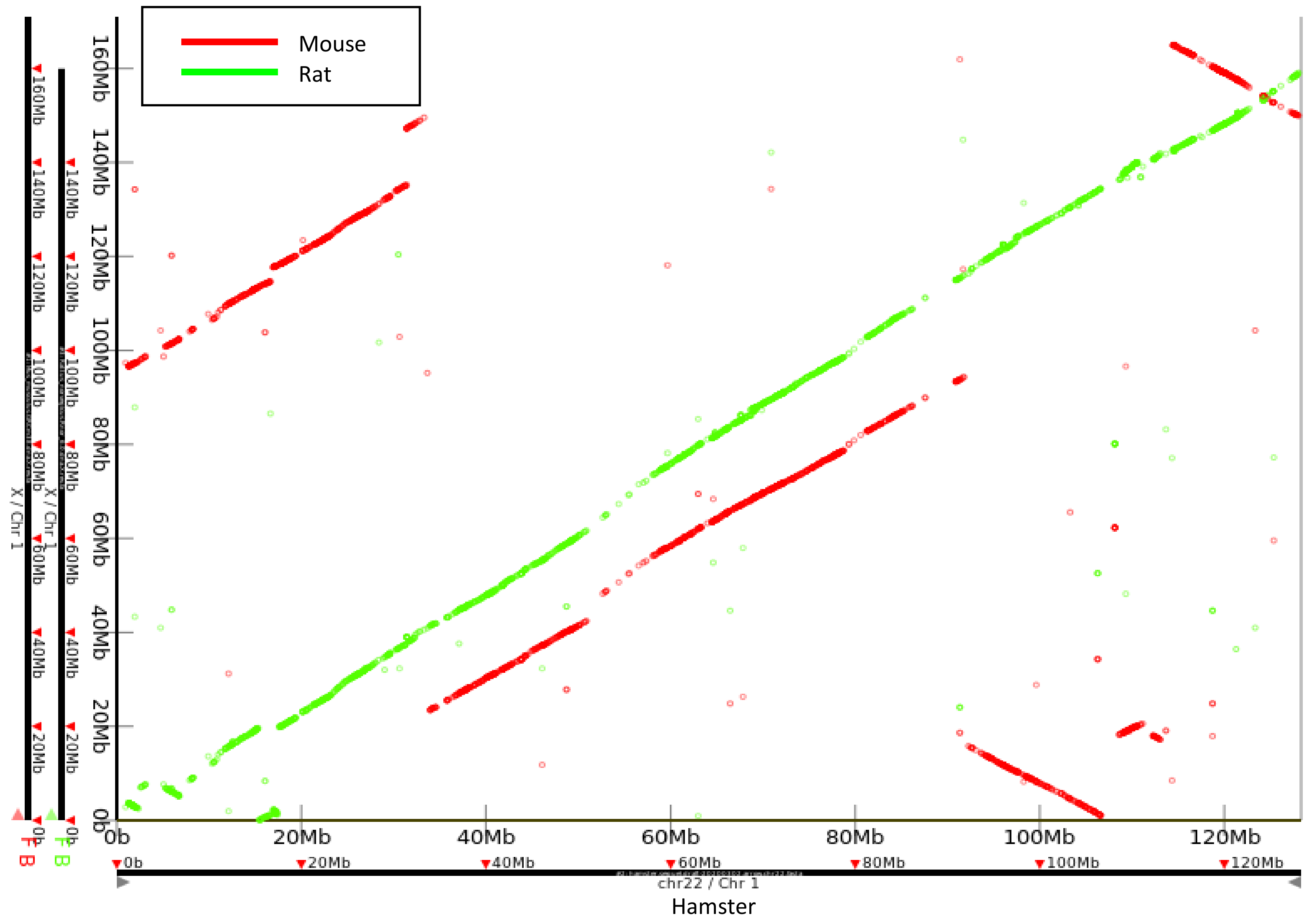

¥
