# Supplementary material for: Hamster PIWI proteins bind to piRNAs with stage-specific size variations during oocyte maturation
Source: Nucleic Acids Res. 2021 Feb 15;49(5):2700–20. doi: 10.1093/nar/gkab059 (PMC7969018; doi:10.1093/nar/gkab059)

Figure S1

Ishino et al.

A

Gene name	Drosophila homolog	Expression level (TPM) Testis	Ovary	Oocyte
Piwil1	Aub	24.24	4.63	20.49
Piwil2	Ago3	14.07	0.00	2.97
Piwil3	-	0.00	16.28	14.60
Piwil4	-	2.55	0.00	0.00
GASZ (Asz1)	GasZ	6.45	2.29	4.20
GTSF1	Asterix/Gtsf1	54.03	2.26	11.10
Henmt1	Hen1	3.25	2.24	18.50
Mael	Mealstrom	389.23	14.05	109.81
MitoPLD (Pld6)	Zucchini	3.83	0.18	12.38
Mov10l1	Armitage	41.34	0.00	12.79
MVH (Ddx4)	Vasa	365.43	27.26	1163.69
Pnldc1	- (Trimmer in silkworm)	12.44	4.94	2.51
SETDB1	Eggless	39.88	26.75	459.95
TDRD1	Vreteno	158.01	19.77	455.42
TDRD2 (Tdrkh)	Papi	12.32	1.42	10.38
TDRD4 (Rnf17)	Qin/Kumo	88.61	5.94	36.19
TDRD5	Tejas	92.64	4.57	3.94
TDRD6	Tudor	15.56	0.00	0.00
TDRD7	Tapas	79.80	10.18	81.32
TDRD9	SpnE	49.70	1.43	0.00
TDRD12	BoYb	0.64	0.54	0.54

0 2 4 6 8 ≧10

B

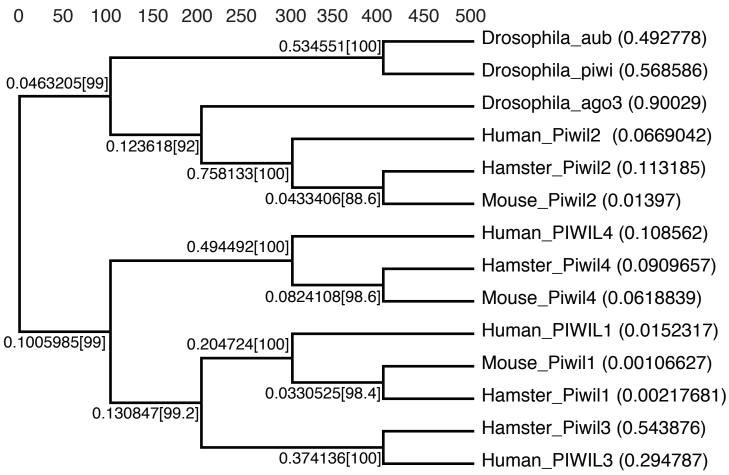

C

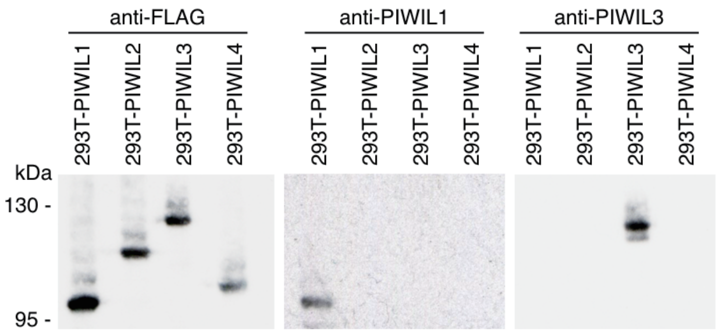

Figure S2

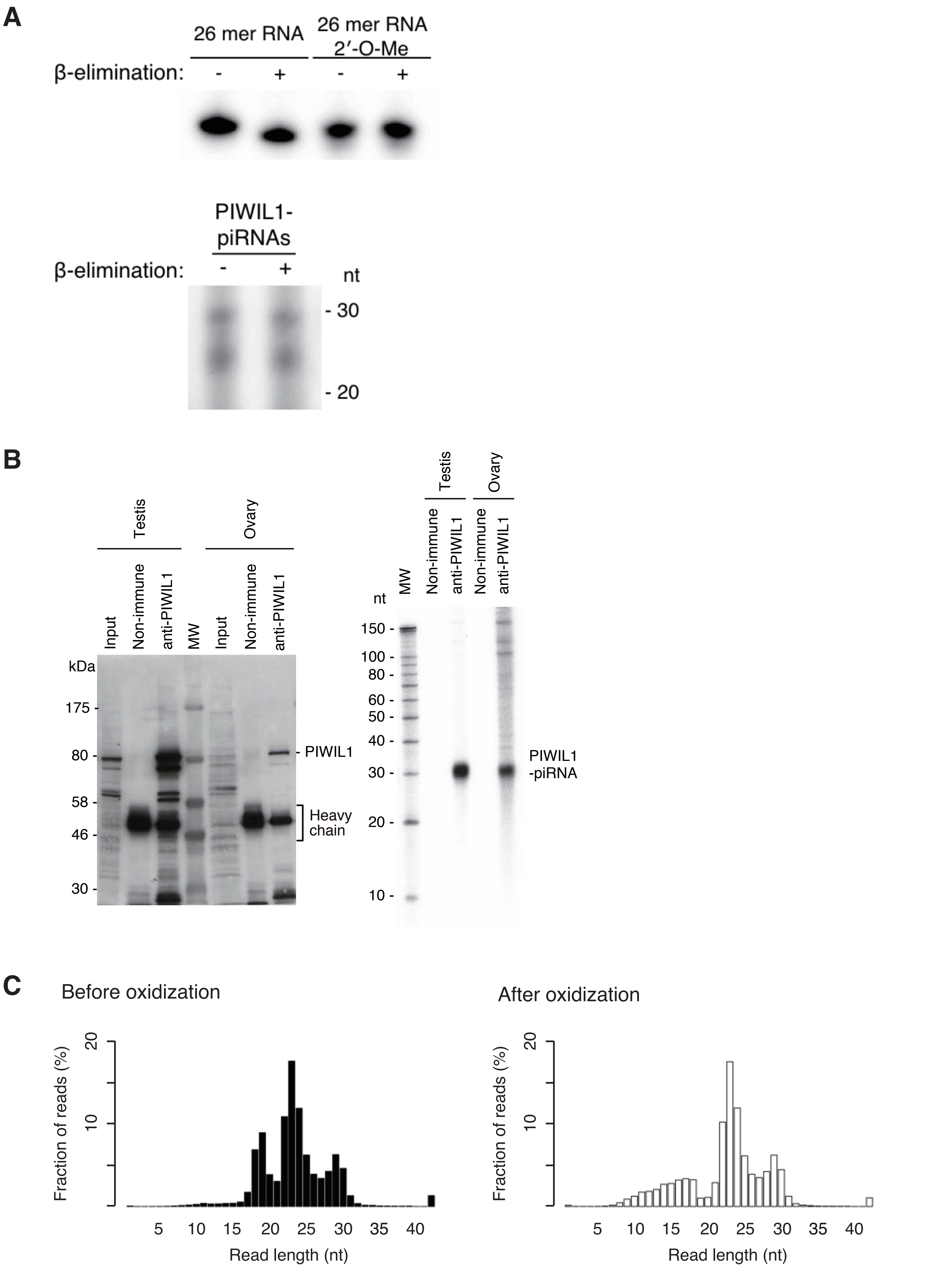
A
26 mer RNA
26 mer RNA 2′-O-Me
β-elimination: - + - +
PIWIL1-piRNAs
β-elimination: - +
nt
- 30
- 20
B
Testis
Ovary
Input
Non-immune
anti-PIWIL1
MW
kDa
175
80
58
46
30
- PIWIL1
Heavy chain
nt
150
100
80
60
50
40
30
20
10
PIWIL1 -piRNA
C
Before oxidization
After oxidization
Fraction of reads (%)
0
10
20
5 10 15 20 25 30 35 40
Read length (nt)

Figure S3

Ishino et al.

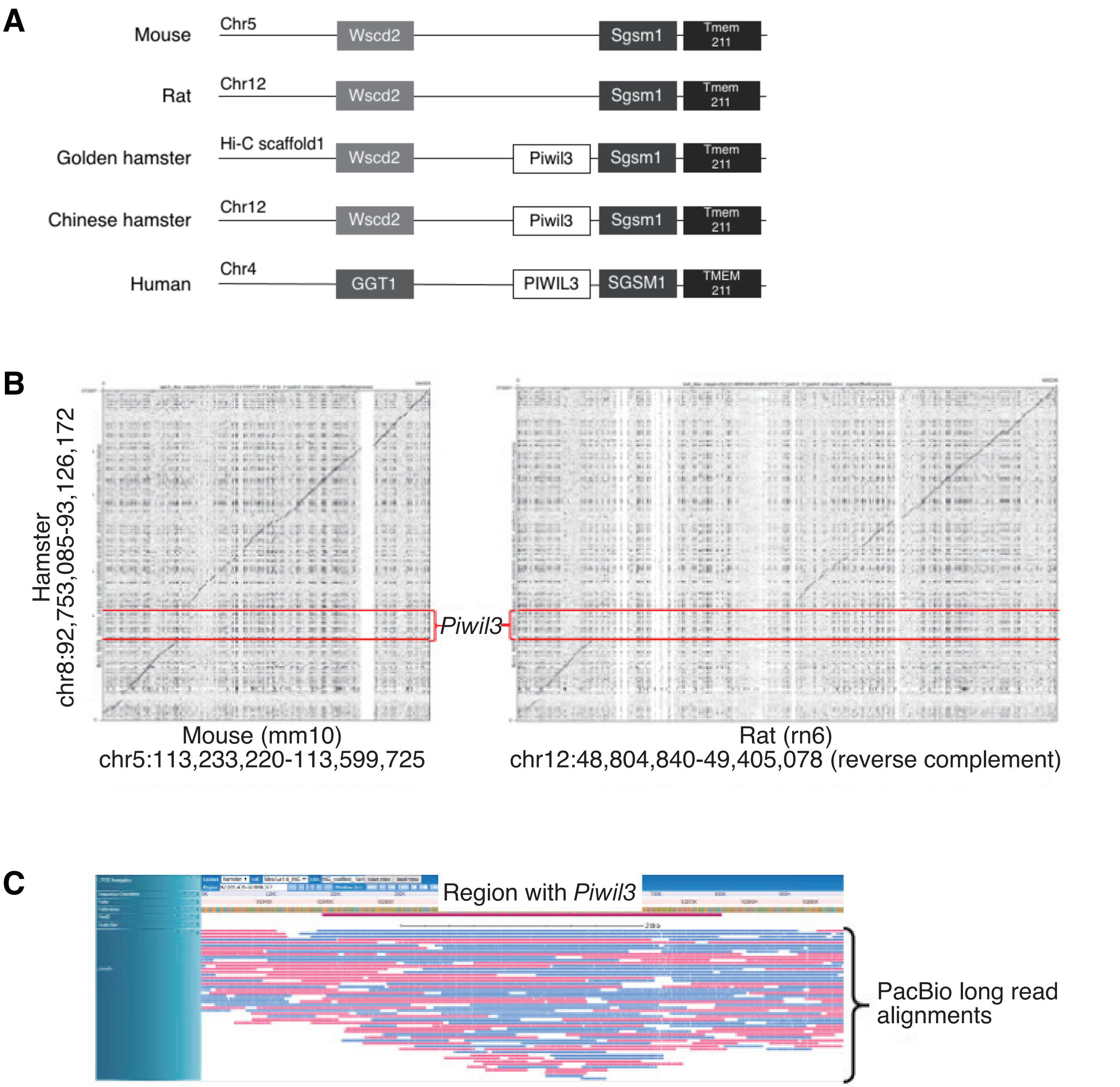

Figure S4

Ishino et al.

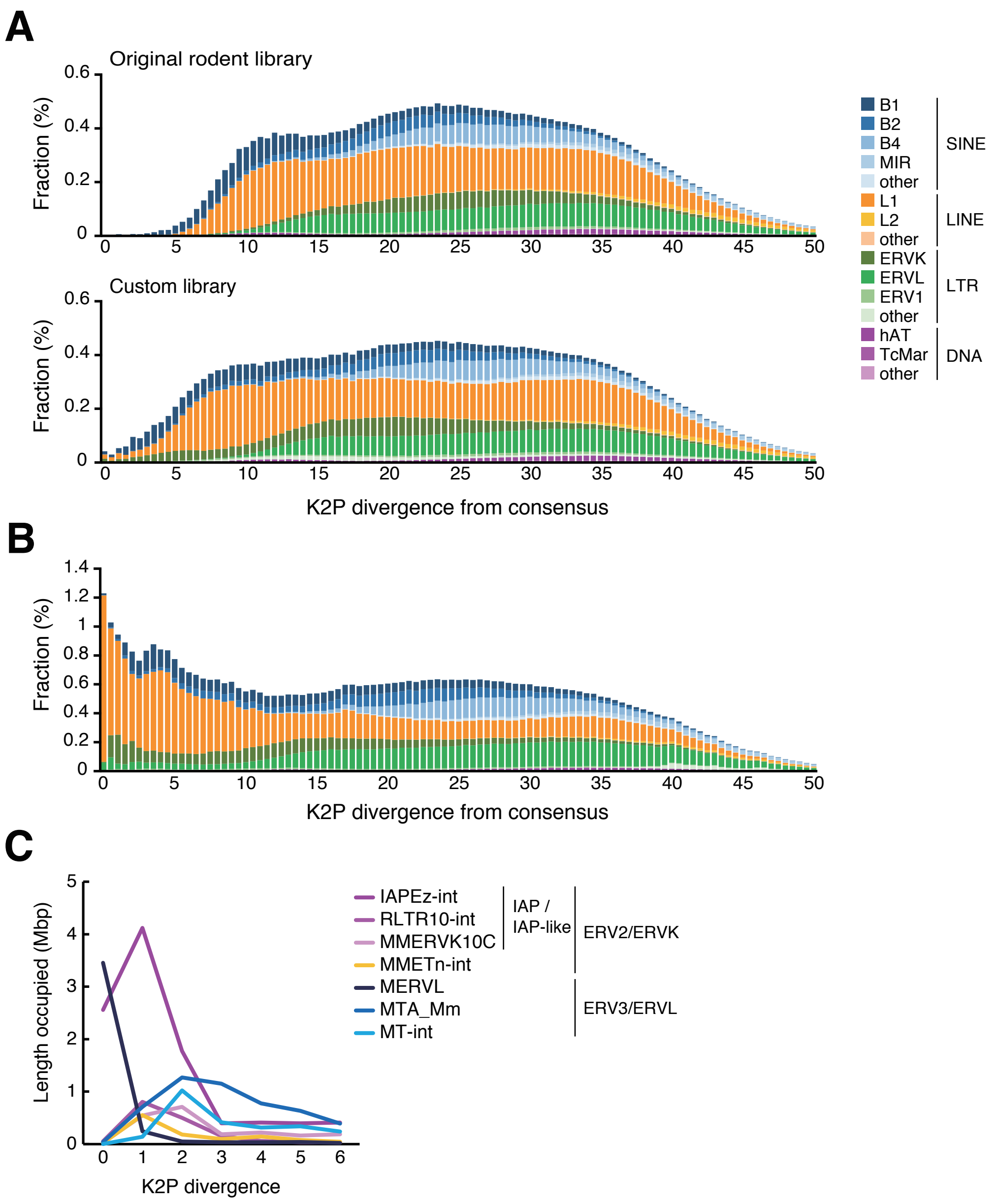

Figure S5

Ishino et al.

Testis_PIWIL1_27_32

Read frequency (%)	piRNA reads	Repeat	Class
4.32	0.02	L2c_3end#LINE/L2	LINE
2.89	76.97	L1-4d_MAu#LINE/L1	LINE
2.88	64.96	L1-3_MAu#LINE/L1	LINE
2.49	67.09	L1-2_CGr#LINE/L1	LINE
2.38	99.84	Lx5c_3end#LINE/L1	LINE
1.32	83.84	L1-4e_MAu#LINE/L1	LINE
1.30	75.02	L1-4a_MAu#LINE/L1	LINE
1.25	83.62	L1-4f_MAu#LINE/L1	LINE
1.16	75.74	L1-4b_MAu#LINE/L1	LINE
1.10	77.18	L1-4c_MAu#LINE/L1	LINE
0.54	73.59	B1b_MAu#SINE/Alu	SINE
0.45	6.13	MIR3#SINE/MIR	SINE
0.44	41.34	B1a_MAu#SINE/Alu	SINE
0.30	99.37	PB1D11#SINE/Alu	SINE
0.25	92.54	B1_Mur4#SINE/Alu	SINE
0.23	93.52	B1_Mur3#SINE/Alu	SINE
0.20	24.31	ID_B1#SINE/B4	SINE
0.17	42.93	B2_MAu#SINE/B2	SINE
0.13	62.59	B2_Rat2#SINE/B2	SINE
0.12	75.59	B1_Mus1#SINE/Alu	SINE
8.44	97.31	MTE2a_MAu#LTR/ERVL-MaLR	LTR
7.13	61.02	IAP1Ea_MAu-I#LTR/ERVK	LTR
3.61	0.18	RMER15#LTR/ERVL	LTR
1.98	13.53	ORR1B2#LTR/ERVL-MaLR	LTR
1.65	7.35	LTR11d_MAu#LTR	LTR
1.63	69.49	ERV2-7a_MAu-I#LTR/ERVK	LTR
1.59	82.80	IAP1D_MAu-I#LTR/ERVK	LTR
1.59	80.09	ERV2-7d_MAu-I#LTR/ERVK	LTR
1.56	6.57	LTR11e_MAu#LTR	LTR
1.35	77.03	ERV2-7c_MAu-I#LTR/ERVK	LTR
0.75	92.46	URR1B#DNA/hAT-Charlie	DNA
0.58	83.76	URR1A_MAu#DNA/hAT-Charlie	DNA
0.50	90.46	URR1A#DNA/hAT-Charlie	DNA
0.03	100.00	Cheshire#DNA/hAT-Charlie	DNA
0.02	100.00	MER58B#DNA/hAT-Charlie	DNA
0.02	100.00	MER112#DNA/hAT-Charlie	DNA
0.01	100.00	Charlie9#DNA/hAT-Charlie	DNA
0.00	0.00	RCHARR1#DNA/hAT-Charlie	DNA
0.00	100.00	Charlie1#DNA/hAT-Charlie	DNA
0.00	100.00	Tigger1#DNA/TcMar-Tigger	DNA

Ovary_PIWIL1_28_31

Read frequency (%)	piRNA reads	Repeat	Class
0.56	20.23	L1-4d_MAu#LINE/L1	LINE
0.49	19.16	L1-4c_MAu#LINE/L1	LINE
0.45	18.33	L1-4a_MAu#LINE/L1	LINE
0.32	42.30	L1-4f_MAu#LINE/L1	LINE
0.30	6.56	L1-4b_MAu#LINE/L1	LINE
0.28	61.78	L1-2_CGr#LINE/L1	LINE
0.25	82.73	L1-4e_MAu#LINE/L1	LINE
0.14	28.95	L1-4g_MAu#LINE/L1	LINE
0.14	90.77	L1-3_MAu#LINE/L1	LINE
0.10	14.05	L1-5b_MAu#LINE/L1	LINE
0.04	30.84	B1b_MAu#SINE/Alu	SINE
0.01	23.08	B2_MAu#SINE/B2	SINE
0.01	20.00	B1a_MAu#SINE/Alu	SINE
0.01	90.91	B3#SINE/B2	SINE
0.01	0.00	B2_Rat2#SINE/B2	SINE
0.01	44.44	B1_Mm#SINE/Alu	SINE
0.01	94.44	ID_B1#SINE/B4	SINE
0.00	0.00	7SLRNA_short_#SINE/Alu	SINE
0.00	75.00	B2_Rat1#SINE/B2	SINE
0.00	83.33	B2_Rat4#SINE/B2	SINE
22.47	95.42	ERV2-5b_MAu-I#LTR/ERVK	LTR
11.82	93.25	ERV2-5a_MAu-I#LTR/ERVK	LTR
9.45	97.12	ERV2-11_MAu-I#LTR/ERVK	LTR
5.70	99.88	ERV2-14a_MAu-LTR#LTR/ERVK	LTR
4.76	99.90	ERV2-17b_MAu-I#LTR/ERVK	LTR
4.50	79.22	ERV2-7a_MAu-I#LTR/ERVK	LTR
4.26	78.00	ERV2-7d_MAu-I#LTR/ERVK	LTR
4.08	99.78	ERV2-4_CGr-I#LTR/ERVK	LTR
4.01	99.92	ERV2-17a_MAu-I#LTR/ERVK	LTR
3.78	99.93	ERV2-17c_MAu-I#LTR/ERVK	LTR
0.03	85.29	URR1A_MAu#DNA/hAT-Charlie	DNA
0.03	93.48	URR1A#DNA/hAT-Charlie	DNA
0.02	92.75	URR1B#DNA/hAT-Charlie	DNA
0.00	0.00	MER53#DNA/hAT-Blackjack	DNA
0.00	100.00	MER5B#DNA/hAT-Charlie	DNA

Oocyte_PIWIL3_18_20

Read frequency (%)	piRNA reads	Repeat	Class
2.63	0.59	L3#LINE/CR1	LINE
2.18	0.89	L1-4f_MAu#LINE/L1	LINE
1.72	1.01	L1-4d_MAu#LINE/L1	LINE
0.78	100.00	Lx5b_3end#LINE/L1	LINE
0.56	9.59	L1M8_5end#LINE/L1	LINE
0.51	0.12	L1MdMus_I_orf2#LINE/L1	LINE
0.47	15.36	L1-2_Dor#LINE/L1	LINE
0.41	0.41	L1_Mur1_5end#LINE/L1	LINE
0.34	86.38	HAL1-3A_Cpo#LINE/L1	LINE
0.34	97.98	L1_Rat3_orf2#LINE/L1	LINE
0.83	0.78	B1_Mm#SINE/Alu	SINE
0.19	100.00	B1_Rn#SINE/Alu	SINE
0.09	5.61	B3A#SINE/B2	SINE
0.06	91.24	ID_B1#SINE/B4	SINE
0.03	23.08	B3#SINE/B2	SINE
0.03	1.59	B2_MAu#SINE/B2	SINE
0.02	14.94	B1a_MAu#SINE/Alu	SINE
0.02	89.52	SINEB1_Mu#SINE/Alu	SINE
0.00	6.12	B2_Rat2#SINE/B2	SINE
0.00	0.00	DIPODE3#SINE/7SL	SINE
15.34	38.13	ERV2-5b_MAu-I#LTR/ERVK	LTR
5.22	0.26	RodERV21#LTR/ERV1	LTR
4.61	98.84	ERV2-3_CGr-I#LTR/ERVK	LTR
4.19	95.22	ERV2-14b_MAu-LTR#LTR/ERVK	LTR
3.34	99.96	LTR8B_Cpo#LTR/ERV1	LTR
3.18	1.81	MYSERV_Rn#LTR/ERVK	LTR
2.42	0.15	RLTR14-int#LTR/ERV1	LTR
2.37	91.97	ERV2-11_MAu-I#LTR/ERVK	LTR
1.97	94.15	LTR13_MAu#LTR	LTR
1.65	99.26	MMERGLN-int#LTR/ERV1	LTR
0.21	3.70	AmnHarb1#DNA/PIF-Harbinger	DNA
0.17	73.29	Charlie7#DNA/hAT-Charlie	DNA
0.17	98.21	Chap1a_Mam#DNA/hAT-Charlie	DNA
0.16	92.56	Charlie16#DNA/hAT-Charlie	DNA
0.15	52.51	OldhAT1#DNA/hAT-Ac	DNA
0.15	4.66	Charlie10#DNA/hAT-Charlie	DNA
0.13	93.26	Zaphod3#DNA/hAT-Tip100	DNA
0.13	98.78	Cheshire#DNA/hAT-Charlie	DNA
0.10	21.63	Zaphod#DNA/hAT-Tip100	DNA
0.10	11.99	Arthur1#DNA/hAT-Tip100	DNA

OoL_PIWIL1_28_31

Read frequency (%)	piRNA reads	Repeat	Class
1.25	27.77	L1-4d_MAu#LINE/L1	LINE
0.87	38.28	L1-4c_MAu#LINE/L1	LINE
0.83	41.29	L1-4a_MAu#LINE/L1	LINE
0.37	5.82	L1-4b_MAu#LINE/L1	LINE
0.34	33.71	L1-4f_MAu#LINE/L1	LINE
0.29	32.48	L1-4e_MAu#LINE/L1	LINE
0.17	57.63	L1-4g_MAu#LINE/L1	LINE
0.09	35.56	L1-2_CGr#LINE/L1	LINE
0.06	100.00	L1_Rod2_orf2#LINE/L1	LINE
0.05	100.00	Lx11_3end#LINE/L1	LINE
0.01	0.00	7SLRNA_short_#SINE/Alu	SINE
0.01	0.00	P7SL_Cpo#SINE/7SL	SINE
0.01	84.21	B1_Mus1#SINE/Alu	SINE
0.01	94.44	B1_Rn#SINE/Alu	SINE
0.01	94.12	B1_Mur3#SINE/Alu	SINE
0.01	100.00	B1_Mur4#SINE/Alu	SINE
0.01	0.00	7SLRNA#SINE/Alu	SINE
0.01	100.00	B1_Mus2#SINE/Alu	SINE
0.00	16.67	B2_MAu#SINE/B2	SINE
0.00	0.00	B1b_MAu#SINE/Alu	SINE
17.45	93.86	ERV2-5a_MAu-I#LTR/ERVK	LTR
13.18	94.91	ERV2-5b_MAu-I#LTR/ERVK	LTR
8.74	99.98	ERV2-17b_MAu-I#LTR/ERVK	LTR
7.97	100.00	ERV2-17a_MAu-I#LTR/ERVK	LTR
6.42	100.00	ERV2-17c_MAu-I#LTR/ERVK	LTR
4.08	63.05	ERV2-7d_MAu-I#LTR/ERVK	LTR
3.72	0.64	IAP1Ea_MAu-I#LTR/ERVK	LTR
3.33	91.91	ERV2-11_MAu-I#LTR/ERVK	LTR
3.14	49.46	ERV2-7a_MAu-I#LTR/ERVK	LTR
3.00	53.72	ERV2-7c_MAu-I#LTR/ERVK	LTR
0.00	0.00	URR1B#DNA/hAT-Charlie	DNA

OoS_PIWIL1_21_27

Read frequency (%)	piRNA reads	Repeat	Class
1.07	11.34	L1-4d_MAu#LINE/L1	LINE
0.85	7.30	L1-4a_MAu#LINE/L1	LINE
0.57	29.32	L1-2_CGr#LINE/L1	LINE
0.54	10.38	L1-4c_MAu#LINE/L1	LINE
0.53	7.51	L1-4b_MAu#LINE/L1	LINE
0.52	52.53	L1-4f_MAu#LINE/L1	LINE
0.39	63.53	L1-4e_MAu#LINE/L1	LINE
0.30	69.68	L1-4g_MAu#LINE/L1	LINE
0.14	61.47	L1-5b_MAu#LINE/L1	LINE
0.13	59.34	L1-5a_MAu#LINE/L1	LINE
0.02	10.83	B1_Mm#SINE/Alu	SINE
0.01	3.92	ID_B1#SINE/B4	SINE
0.01	0.00	ID2#SINE/ID	SINE
0.01	0.00	ID4#SINE/ID	SINE
0.01	0.00	ID4_v#SINE/ID	SINE
0.01	0.00	P7SL_Cpo#SINE/7SL	SINE
0.01	0.00	7SLRNA_short_#SINE/Alu	SINE
0.01	0.00	7SLRNA#SINE/Alu	SINE
0.00	0.00	B2_MAu#SINE/B2	SINE
0.00	0.00	SINEB2_Mu#SINE/B2	SINE
10.37	59.83	ERV2-5b_MAu-I#LTR/ERVK	LTR
8.57	20.31	ERV2-3_CGr-I#LTR/ERVK	LTR
6.26	82.61	ERV2-5a_MAu-I#LTR/ERVK	LTR
4.93	100.00	ERV2-17b_MAu-I#LTR/ERVK	LTR
4.40	99.99	ERV2-17a_MAu-I#LTR/ERVK	LTR
4.33	59.77	ERV2-7d_MAu-I#LTR/ERVK	LTR
4.03	99.98	ERV2-17c_MAu-I#LTR/ERVK	LTR
3.61	53.28	ERV2-7a_MAu-I#LTR/ERVK	LTR
3.50	99.41	ERV2-4_CGr-I#LTR/ERVK	LTR
3.38	52.72	ERV2-7c_MAu-I#LTR/ERVK	LTR
0.01	100.00	X34_DNA#DNA	DNA
0.01	100.00	X24_DNA#DNA	DNA
0.00	0.00	OldhAT1#DNA/hAT-Ac	DNA
0.00	66.67	URR1B#DNA/hAT-Charlie	DNA
0.00	46.15	URR1A#DNA/hAT-Charlie	DNA
0.00	0.00	Tigger22N1#DNA/TcMar-Tigger	DNA
0.00	40.00	URR1B_MAu#DNA/hAT-Charlie	DNA
0.00	100.00	Zaphod3#DNA/hAT-Tip100	DNA
0.00	50.00	MER96B#DNA/hAT-Tip100	DNA
0.00	0.00	Zaphod2#DNA/hAT-Tip100	DNA

2C_PIWIL1_20_25

Read frequency (%)	piRNA reads	Repeat	Class
1.05	21.76	L1-4d_MAu#LINE/L1	LINE
0.86	44.67	L1-2_CGr#LINE/L1	LINE
0.82	55.96	L1-4f_MAu#LINE/L1	LINE
0.78	14.75	L1-4a_MAu#LINE/L1	LINE
0.66	70.15	L1-4e_MAu#LINE/L1	LINE
0.64	16.44	L1-4c_MAu#LINE/L1	LINE
0.64	10.43	L1-4b_MAu#LINE/L1	LINE
0.56	69.31	L1-4g_MAu#LINE/L1	LINE
0.24	57.42	L1-5a_MAu#LINE/L1	LINE
0.23	63.31	L1-5b_MAu#LINE/L1	LINE
0.04	4.55	B1_Mm#SINE/Alu	SINE
0.02	0.00	B3A#SINE/B2	SINE
0.02	18.37	ID_B1#SINE/B4	SINE
0.01	0.00	P7SL_Cpo#SINE/7SL	SINE
0.01	75.86	B3#SINE/B2	SINE
0.01	0.00	B2_MAu#SINE/B2	SINE
0.01	0.00	7SLRNA_short_#SINE/Alu	SINE
0.01	0.00	ID4_v#SINE/ID	SINE
0.01	0.00	7SLRNA#SINE/Alu	SINE
0.01	0.00	ID2#SINE/ID	SINE
10.40	68.96	ERV2-5b_MAu-I#LTR/ERVK	LTR
6.37	99.97	ERV2-17b_MAu-I#LTR/ERVK	LTR
5.65	99.84	ERV2-17a_MAu-I#LTR/ERVK	LTR
5.27	100.00	ERV2-17c_MAu-I#LTR/ERVK	LTR
5.10	68.66	ERV2-5a_MAu-I#LTR/ERVK	LTR
4.46	82.47	ERV2-11_MAu-I#LTR/ERVK	LTR
4.34	67.43	ERV2-7d_MAu-I#LTR/ERVK	LTR
4.01	1.20	IAP1Ea_MAu-I#LTR/ERVK	LTR
3.68	61.13	ERV2-7a_MAu-I#LTR/ERVK	LTR
3.31	65.29	ERV2-7c_MAu-I#LTR/ERVK	LTR
0.05	100.00	X34_DNA#DNA	DNA
0.03	97.83	Zaphod3#DNA/hAT-Tip100	DNA
0.02	0.00	OldhAT1#DNA/hAT-Ac	DNA
0.02	1.92	Arthur2#DNA/hAT-Tip100	DNA
0.01	100.00	X24_DNA#DNA	DNA
0.01	54.17	URR1A_MAu#DNA/hAT-Charlie	DNA
0.01	100.00	Eulor5A#DNA/Crypton-A	DNA
0.01	73.68	URR1B#DNA/hAT-Charlie	DNA
0.01	16.67	Tigger22N1#DNA/TcMar-Tigger	DNA
0.01	100.00	Charlie7#DNA/hAT-Charlie	DNA

Sense — Antisense

Figure S6

A

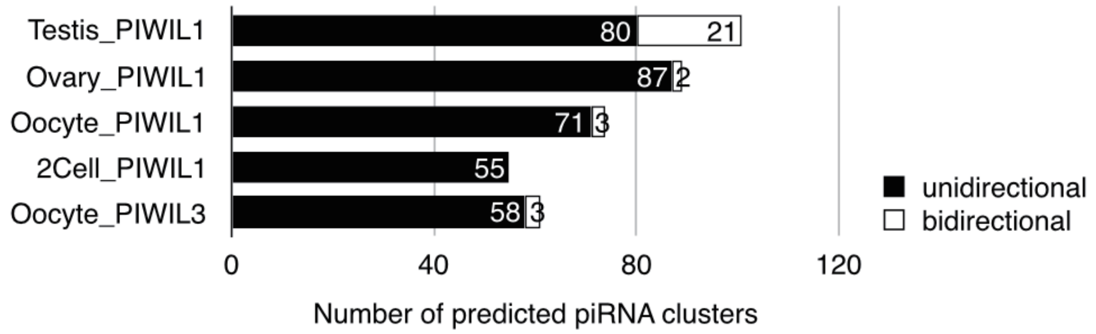

B

Testis PIWIL1 bidirectional piRNA cluster

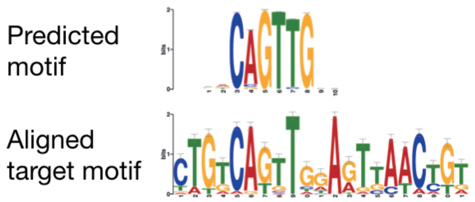

Motif name	p-value
A-Myb	6.67e-04

C

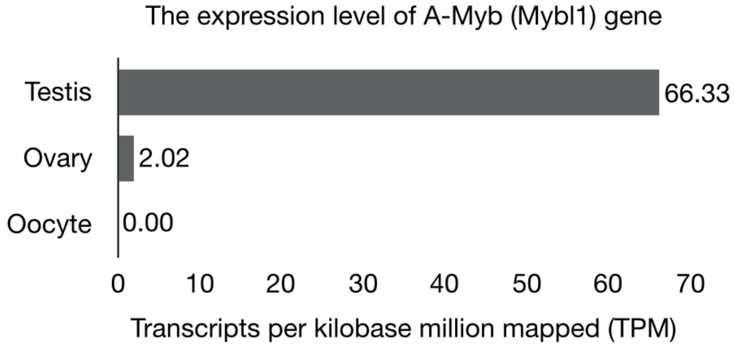

Ishino et al.

Figure S7

Ishino et al.

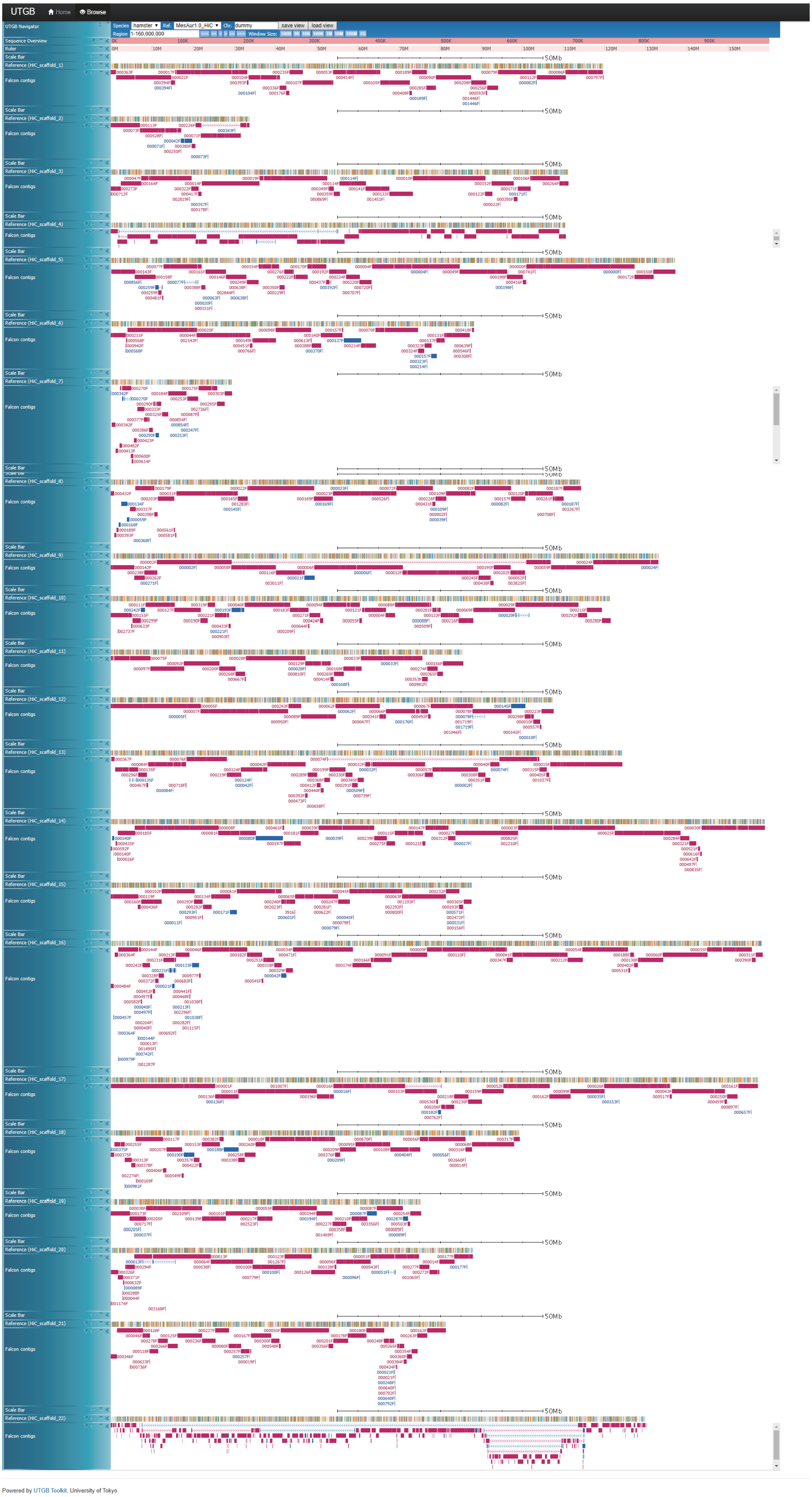

Supplement: gkab059_Supplemental_Files [file gkab059_supplemental_files.zip › 201215_ManuscriptFigure_Ishino_NAR_SupFigs.pdf]
