# Supplementary material for: Hamster PIWI proteins bind to piRNAs with stage-specific size variations during oocyte maturation
Source: Nucleic Acids Res. 2021 Feb 15;49(5):2700–20. doi: 10.1093/nar/gkab059 (PMC7969018; doi:10.1093/nar/gkab059)
Supplement: gkab059_Supplemental_Files [file gkab059_supplemental_files.zip › Supplemental Figures.docx]

**Supplemental Figures:**

**Figure S1. Related to Figure 1.**

(A) Expression levels of homolog genes in hamster testis, ovary, and MII oocyte, which is known to correspond to the PIWI-piRNA pathway in mouse and *Drosophila.* Expression levels are normalized by transcripts per kilobase million mapped (TPM).

(B) A phylogenetic tree of PIWI genes in hamster, human, mouse, and *Drosophila*. Their evolutionary distances were calculated using the maximum likelihood method.

(C) Western blotting was performed on 293T cells, which were transfected with *Piwil1* and *Piwil3* genes, respectively. The results show that anti-MARWI (marmoset PIWIL1) and hamster PIWIL3 antibodies specifically recognize their targets.

**Figure S2. Related to Figure 2.**

(A) PIWIL1-piRNAs were subjected to periodate oxidation and β-elimination treatment. Synthetic RNAs with or without 2′-*O*-methyl modification were used as controls (upper panel). The unchanged PIWIL1-piRNA signals indicate that the 3′-termini of PIWIL1-piRNAs is 2′-*O*-methylated, as the characteristic of piRNAs (lower panel).

(B) Western blotting (left panel) and ^32^P labeling (right panel) were performed from PIWIL1 immunoprecipitates in hamster testis and ovary. Non-immune means negative control, using an anti-mouse IgG antibody.

(C) Composition of small RNAs in the hamster oocytes before and after NaIO4 oxidization. The average result of three biological replicates is shown.

**Figure S3. Related to Figure 3.**

(A) The synteny of the *Piwil3* gene. The synteny of *Wscd2* and *Sgsm1* genes was conserved between hamsters, mice, and rats. Otherwise, a synteny block of *Piwil3*, *Sgsm1*, and *Tmem1* was conserved between hamsters and humans.

(B) From our hamster genome and the reference genomes of mouse (mm10) and rat (rn6), we retrieved and compared the three genomic regions between *Wscd2* and *Sgsm1*. The *Piwil3* encoding region is present in the focal region of the hamster genome but is absent in the mouse and rat genomes. The *Piwil3* region is deleted in the mouse genome and is replaced with another sequencing in the rat genome.

(C) We aligned to PacBio long reads to the assembled contig with *Piwil3* (the red-colored region). In the lower portion, the respective blue and red colored lines show the alignments of reads in the plus and minus strands. We observed that each base position was covered by an ample number (ten or more) of long reads, and the read coverage was nearly even, thereby confirming the accuracy of the assembled contig.

**Figure S4. Related to Figure 4.**

Age distribution of TEs in another hamster assembly (DNA ZOO MesAur1.0_HiC) and mouse genome. The proportion of TEs is shown for 0.5 bins of K2P distance (CpG-corrected) from each consensus sequence.

(A) TEs in the MesAur1.0_HiC assembly was analyzed with the original (top) and customized (bottom) repeat libraries.

(B) TEs in the mouse genome was analyzed using the mouse repeat library. The color code is the same as in (A).

(C) Representative young LTR retrotransposons in the mouse genome.

**Figure S5. Related to Figure 6.**

Heat maps show strand bias of transposon-derived piRNAs corresponding to each PIWIs in hamster testis, ovary, MII oocyte and 2-cell embryo. Transposons are grouped into LINEs, SINEs, LTRs, and DNA transposons. Color intensities indicate the degree of strand bias: (blue) sense; (yellow) antisense; (white) unbiased. The frequencies of piRNAs mapped to each TE subfamily over the total TE-mapped piRNAs are shown in the bar graph.

**Figure S6. Related to Figure 8.**

(A) The number of predicted piRNA clusters in hamster testis, ovary, MII oocyte and 2-cell embryo. The black bar shows the unidirectional piRNA clusters and white shows the bidirectional piRNA clutsters, respectively.

(B) A motif search was performed for each unidirectional and bidirectional piRNA cluster in the hamster testis using MEME. The results suggest that the A-Myb binding site is significantly represented in the bidirectional piRNA clusters in testis transcriptional start site surrounding regions.

(C) Expression levels of transcription factor candidates detected using MEME. The expression pattern of the A-Myb gene in male and female hamsters. RNA-seq data were obtained using Illumina HiSeq2000. The x-axis suggests the normalized expression level (TPM).

**Figure S7. Contigs aligned to the reference assembly.**

The 22 panels show the alignments of our assembled contigs to the 22 Hi-C scaffolds of the DNA Zoo Hi-C assembly. Thick red and blue lines show alignments of contigs in the plus and minus strands, respectively. Labels beside each contig line indicate the contig identifier.

**Figure S8. Map of conserved synteny between the hamster, mouse, and rat genomes.**

(A) Each chromosome in the hamster (top), mouse (middle), and rat (bottom) genomes are associated with a two-column box. The left and right columns show chromosomes in the genomes of mouse and rat (top row), hamster and rat (middle row), and hamster and rat (bottom row), respectively. The same color-coding of chromosomes shown in the color pallet at the bottom was used for the three species.

(B) The upper portion is identical to Figure 1B. To facilitate the identification of synteny blocks, dot plots of the hamster (x-axis) and mouse (y-axis) genomes (bottom left), and the hamster (x-axis) and rat (y-axis) genomes (bottom right) are shown.

(C) Left panel, color-coded lines showing reciprocally best-matching pairs of positions in the hamster (middle) and mouse (top) or rat chromosomes (bottom) for each of the 22 hamster chromosomes. Right panel: synteny blocks between the hamster genome on the x-axis and the mouse (red) and rat (green) genomes on the y-axis.

**Table S1. Related to Table 1.**

Statistics of raw and “polished” long genome sequencing reads from the golden hamster sample using the PacBio Sequel system.

**Table S2. Related to Table 1.**

Series of connected contigs and error-corrected reads. For each chromosomal position, the first and penultimate columns list primary contigs obtained using the FALCON genome assembler (yellow), error-corrected reads (light blue), and remaining gaps (white). The fourth and fifth columns show the start and end positions for each contig, respectively.

**Table S3. Related to Table 1.**

Mismatch and indel ratios of our golden hamster genome assembly before and after polishing using PacBio long reads.

**Table S4. Related to Table 2**.

Proportion (%) of transposable elements in the MesAur1.0_HiC assembly.
